# Reviving Dormant Immunity: Millimeter Waves Reprogram the Immunosuppressive Microenvironment to Potentiate Immunotherapy without Obvious Side Effects

**DOI:** 10.34133/cbsystems.0468

**Published:** 2025-12-10

**Authors:** Zhenqi Jiang, Rui Jing, Ozioma Udochukwu Akakuru, Keyi Li, Xiaoying Tang

**Affiliations:** ^1^ School of Medical Technology, Beijing Institute of Technology Zhengzhou Academy of Intelligent Technology, Beijing Institute of Technology, Beijing 100081, China.; ^2^ Department of Chemical and Petroleum Engineering, Schulich School of Engineering, University of Calgary, Calgary, AB T2N 1N4, Canada.

## Abstract

Addressing the variability in cancer immunotherapeutic outcomes among patients and the challenge of devising safe strategies to overcome immune evasion in solid tumors are crucial in advancing cancer therapy. This study investigated the anti-tumor effect of millimeter waves (MMWs) alone and in combination with the anti-programmed cell death-ligand 1 (α-PD-L1) antibody in a 4T1 “cold tumor” model. The results show that MMWs not only inhibit tumor growth but also improve tumor metabolism and the immune microenvironment and enhance anti-tumor immune responses by inducing conformational changes of key immune proteins. Further experiments conducted on cellular and animal models demonstrated that the anti-tumor efficacy of MMWs, which plays a pivotal role, was substantially enhanced with the aid of α-PD-L1. This collaboration resulted in a synergistic effect that not only inhibited tumor progression but also promoted a sustained immune response and prevented recurrence. The additional CT26 “cold tumor” model validates the applicability of this strategy across other “cold tumor” types, particularly in reprogramming the immunosuppressed state of “cold tumor”. These findings underscore the unique potential of MMWs as a nonionizing, nonthermal therapeutic tool that complements cancer immunotherapy, offering a novel approach for the precision treatment of solid tumors.

## Introduction

Overcoming tumor immune escape mechanisms, in which malignant cells evade immune surveillance through molecular adaptations, represents a critical barrier to achieving sustained tumor-free survival following cancer immunotherapy [1–3]. The incorporation of immune checkpoint inhibitors has the potential to reactivate the body’s inherent anti-tumor immune response [4]. However, clinical data indicate that most solid tumors still exhibit a limited response rate to immune checkpoint therapy [5]. These “cold tumors” often lack sufficient infiltrating lymphocytes, and their immune microenvironment is not conducive to the activation and recruitment of immune cells, resulting in a poor response to immunotherapy [6,7]. In contrast, even “hot tumors” often produce a subset of tumor cell clones that either do not respond to immunotherapy or develop resistance to it [8]. The underlying cause of this phenomenon is that tumor cells evade and ultimately suppress the activation and recruitment of cytotoxic immune cells in their microenvironment through various molecular mechanisms [9]. Among these mechanisms, CD47 plays a crucial role [10]. CD47 is widely expressed on the surface of tumor cells and serves as a major mediator of “nonphagocytic” signals. When it binds to the Sirp-alpha-3p84 (SIRPα) receptor on the surface of macrophages, it effectively inhibits macrophage phagocytosis [11,12].

Several novel antibody therapies targeting this axis have undergone clinical trials in recent years and are expected to play a pivotal role in the next generation of immunotherapy [13–15]. Currently, immune checkpoint inhibitors have garnered attention for tumor immunotherapy. These therapies can suppress tumor cells by exploiting immune checkpoint molecules to inhibit effector T cells and restore the body’s cytotoxic immune response [16,17]. However, monotherapy faces numerous challenges, including low response rates in a majority of patients, the development of drug resistance in tumor cells, and the limitations of broad-spectrum treatments [18,19]. Therefore, new synergistic approaches are urgently needed to enhance therapeutic efficacy [20]. In recent years, the field of “physical biomedical science” has garnered global attention [21,22]. This interdisciplinary domain investigates the biological effects of physical stimuli (e.g., sound, light, electricity, magnetism, force, and heat) to develop innovative nondrug therapeutic modalities [23]. Currently, both single and synergistic treatments utilizing various physical factors (e.g., radiation therapy) are widely employed in clinical practice. These approaches have proven effective in activating the immunogenicity of tumor cells and enhancing the body’s immune response [24]. However, most of these physical therapies rely on high-energy ionizing radiation, which inevitably damages normal tissues and results in severe systemic toxic side effects [25–27]. Therefore, there is an urgent need for nonthermal, nonionizing physical therapies that offer potential safety advantages [28,29].

As an emerging nonthermal and nonionizing physical factor, millimeter waves (MMWs) have shown unique advantages in tumor immunotherapy [30–33]. According to the theory of resonance absorption, macromolecules such as DNA, RNA, proteins, and biological membranes in organisms possess specific oscillating frequencies that fall within the MMW range [34–37]. When MMWs irradiate an organism at their resonance frequency, the macromolecules and membrane structures undergo resonance absorption, amplifying and dispersing the initially weak wave energy within the body and triggering a series of biological effects [38–40]. The exact mechanisms remain under investigation. It is important to note that MMWs are distinct from general electromagnetic radiation due to their effects on macromolecule structures, primarily inducing conformational changes and modulating activity through nonthermal mechanisms. Furthermore, due to the extremely low energy density of MMW therapy (≤10 mW/cm^2^), it does not cause a temperature increase in biological tissues. Consequently, MMW therapy is regarded as a nonthermal and nonionizing form of physical therapy [41,42]. This distinctive nonthermal and nonionizing characteristic enables precise modulation of key biological processes within the tumor microenvironment while minimizing common systemic toxicity and side effects [43–45]. There is growing evidence that MMWs can regulate gene expression, protein conformation, and cellular metabolic activities, which is anticipated to serve as a powerful complement to immunotherapy [39,46].

Previous studies have established the potential of MMWs in oncology. For instance, Radzievsky et al. [42] demonstrated that 61.22-GHz MMWs inhibit B16F10 melanoma growth through opioid receptor signaling pathways, revealing neuro-immune interactions in tumor suppression. Additionally, 35.2-GHz MMWs were shown to induce apoptosis in A375 melanoma cells via caspase-3/8 activation [47], accompanied by immunogenic cell death marker release, which may enhance antigen exposure in cold tumors. Furthermore, MMWs have been reported to modulate immune responses, such as enhancing natural killer (NK) cell cytotoxicity [48] and promoting macrophage polarization toward a pro-inflammatory phenotype [49], indicating their role in reshaping the tumor immune microenvironment. However, these studies often focus on isolated effects, and a comprehensive investigation into how MMWs remodel the immunosuppressive landscape of cold tumors is lacking. Our study builds on this foundation by systematically evaluating MMWs alone and in combination with anti-programmed cell death-ligand 1 (α-PD-L1) therapy in cold tumor models, aiming to provide a holistic understanding of its translational potential.

**Fig. 1.**
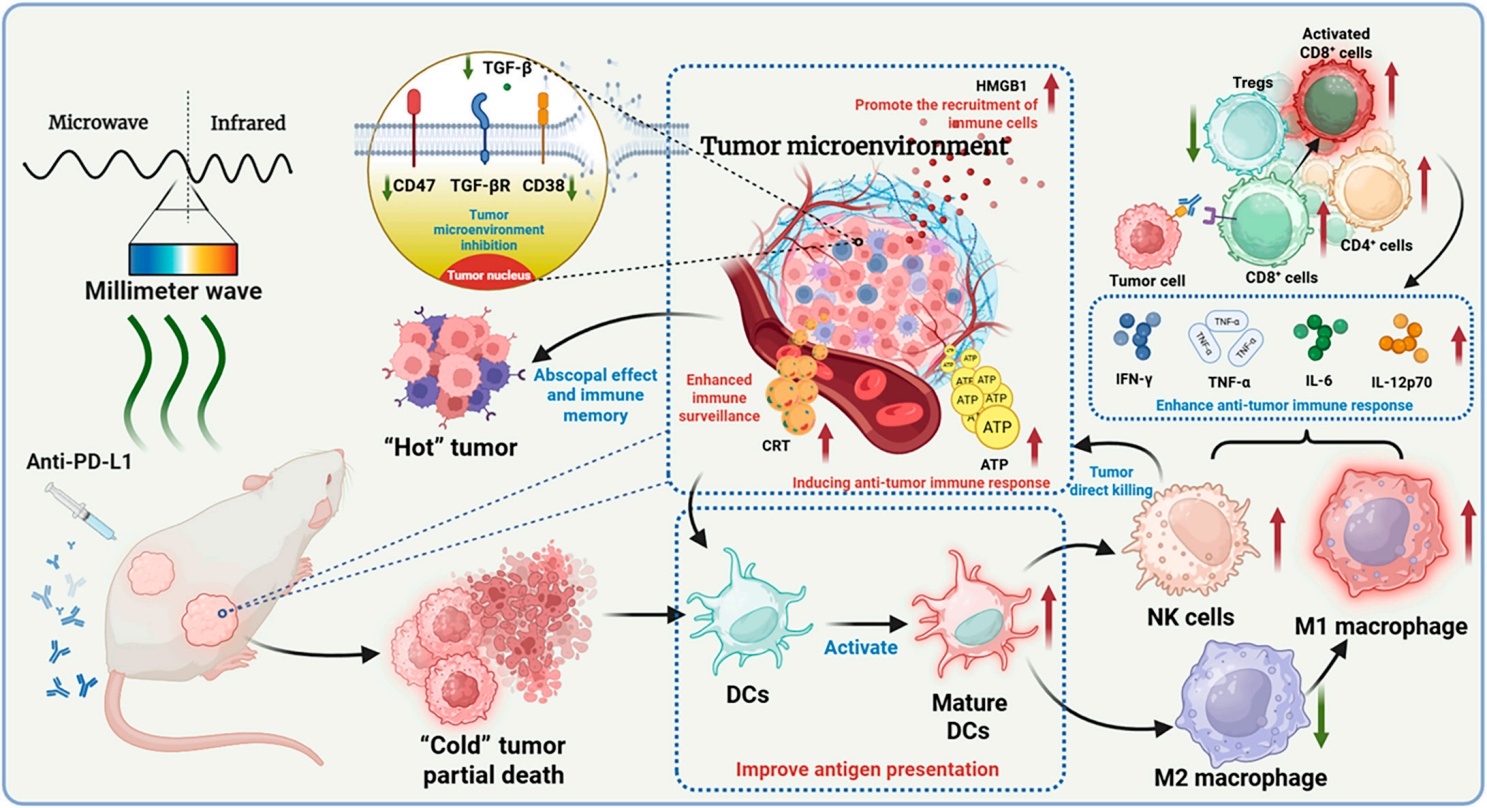
Schematic of millimeter wave (MMW)-driven tumor microenvironment remodeling illustrating MMW therapy modifying the tumor microenvironment to enhance immune response, facilitating the transition from “cold” to “hot” tumors. (This figure is created in BioRender. Jing, R. (2025) https://BioRender.com/uvdmeuv.) PD-L1, programmed cell death-ligand 1; TGF-β, transforming growth factor-β; TGF-βR, TGF-β receptor; HMGB1, high mobility group box-1 protein; CRT, calreticulin; ATP, adenosine triphosphate; DCs, dendritic cells; Tregs, regulatory T cells; IFN-γ, interferon-γ; TNF-α, tumor necrosis factor-α; IL-6, interleukin-6; NK, natural killer.

This study systematically investigated the anti-tumor effects of mono-MMW therapy (35 GHz, 10 mW/cm^2^, close-contact irradiation), both alone and in combination with the immune checkpoint inhibitor α-PD-L1 in 4T1 and CT26 “cold tumors”. The results demonstrated that MMW irradiation alone promotes positive modulation of anti-tumor metabolism and enhances immune-related proteins, thereby remodeling the tumor immune microenvironment. Molecular dynamics simulations revealed how MMWs precisely modulate the conformation and activity of key molecules (e.g., CD47, CD38, and transforming growth factor-β [TGF-β]), interfering with their functions, directly damaging cancer cells, and promoting immunogenicity. Notably, the combination therapy not only significantly inhibits the growth of the primary tumor (with a tumor suppression rate of up to 90%) but also effectively delays the metastatic process, triggers the body’s anti-tumor immune memory, aids in preventing tumor recurrence, and demonstrates a high degree of generalizability (Fig. 1). Furthermore, neither monotherapy nor combination therapy resulted in significant weight loss in mice, indicating its safety. This study not only establishes a theoretical foundation for the application of MMWs in tumor immunotherapy but also introduces new strategies for enhancing the efficacy of immune checkpoint inhibitors and prolonging patient survival. It is plausible to hypothesize that this innovative MMW–immune synergistic therapeutic strategy could represent a promising approach for overcoming tumor immune escape and potentially improving the precision treatment of solid tumors, meriting further investigation.

## Materials and Methods

Information about the materials and methods used in this work is available in the Supplementary Materials.

## Results

### Inhibition of 4T1 breast cancer tumor growth in mice by MMW irradiation and a preliminary investigation of its mechanism

**Fig. 2.**
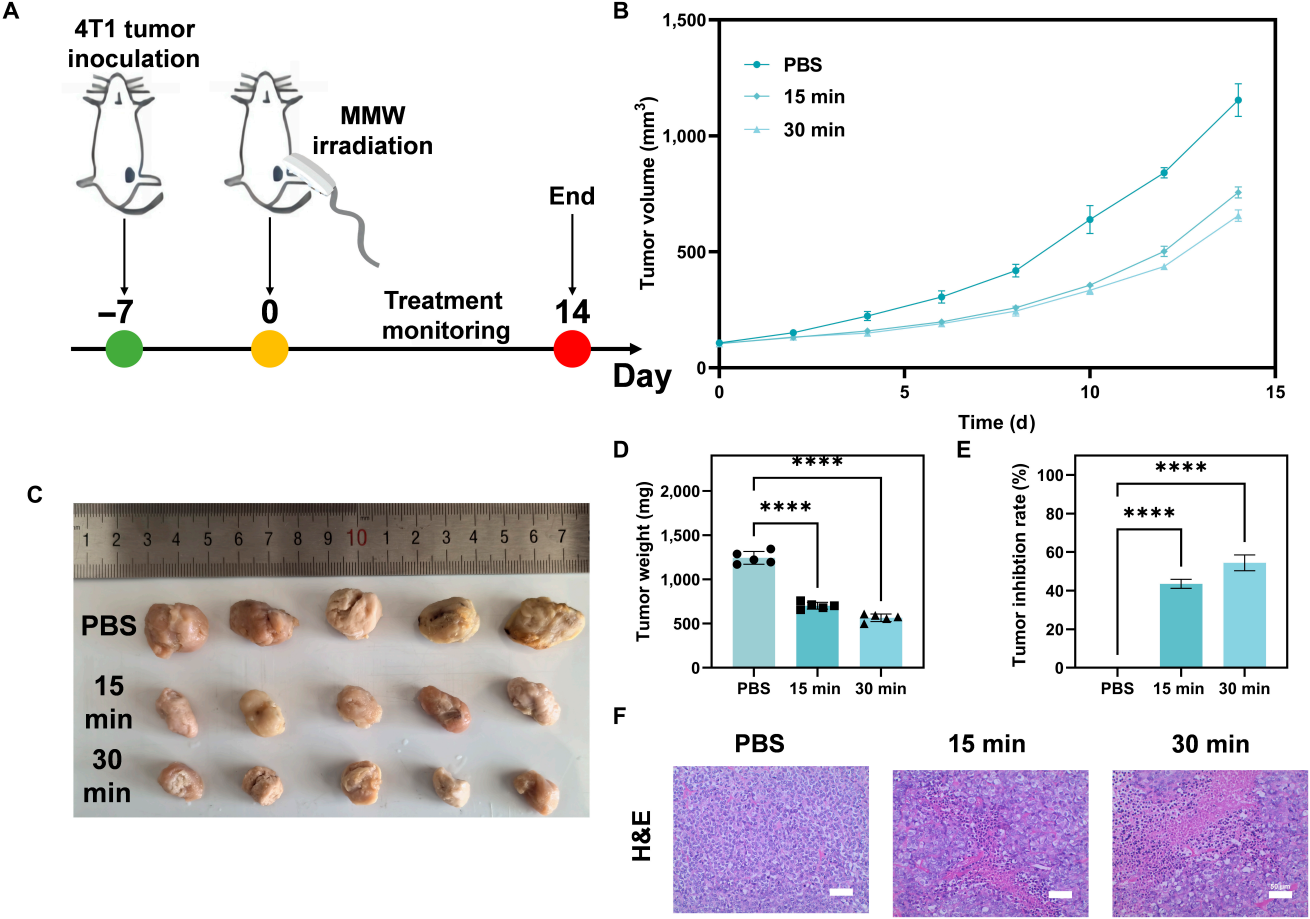
MMWs inhibit 4T1 “cold tumor” growth. (A) Construction of the mouse 4T1 breast cancer model and timeline of MMW irradiation treatment. (B) Relative tumor volume changes in BALB/c mice with early-stage 4T1 tumors after phosphate-buffered saline (PBS) treatment or 15- or 30-min MMW irradiation. (C) Photographs of tumor size in different groups of mice after 15 d of treatment. (D) Tumor weight at the end of the experiment. (E) Tumor growth inhibition rate of different groups. (F) Hematoxylin and eosin (H&E) staining results of tumor tissues in different groups of mice after 15 d of treatment (scale bar: 50 μm). Statistical significance was set as ****P < 0.0001.

To assess the impact of MMW irradiation on primary tumor progression, a 4T1 breast cancer model was established in BALB/c mice. The tumor-bearing mice were then randomized into a control faction (receiving phosphate-buffered saline [PBS] treatment) and 2 experimental groups undergoing MMW irradiation for either 15 or 30 min, as illustrated in Fig. 2A. Throughout the irradiation process, the output MMW power was monitored and remained within an acceptable range of fluctuation (Fig. S1), indicating stable and reproducible irradiation conditions. After 30 min of tightly confined irradiation of the PBS solution using the experimental MMW parameters, only a relatively low temperature increase was observed (Fig. S2). This suggests that the anti-tumor effects observed in vivo were primarily mediated by nonthermal mechanisms.

Tumor volume was measured regularly throughout the study in all groups. Compared to the PBS control group, MMW irradiation inhibited tumor growth in a duration-dependent manner, with the 30-min group showing greater suppression than the 15-min group (Fig. 2B).

The macroscopic anti-tumor effects of MMW treatment were evident. After 14 d of treatment, the tumor volume of mice in the MMW group was significantly smaller than that in the PBS group (Fig. 2C). Quantitative analysis at the endpoint confirmed these findings, demonstrating a significant reduction in tumor weight (Fig. 2D) and an increase in the tumor inhibition rate (Fig. 2E) compared to those of the control group, with both showing clear duration-dependent responses. Notably, no significant differences in body weight were observed among the groups during the treatment period (Fig. S3), suggesting the absence of overt systemic toxicity under the current experimental conditions.

Histopathological examination of harvested tumor tissues was performed. Hematoxylin and eosin (H&E) staining revealed a lower density of viable tumor cells in the MMW-treated groups compared to that in the control group (Fig. 2F).

Collectively, these results demonstrate that noncontact MMW irradiation significantly inhibits the growth of the 4T1 breast cancer model (a “cold tumor”) in mice, primarily through nonthermal mechanisms, with no evident systemic toxicity observed.

### MMWs improve tumor metabolism and the immune microenvironment

Our results demonstrate that MMW irradiation can effectively inhibit the primary tumor growth of 4T1 mammary carcinoma in mice without observed significant systemic toxicity. In order to deepen the understanding of its mechanism of action, a combination of metabolomics, proteomics, Western blot, and flow cytometry analyses was employed​ to assess the molecular changes in the tumor tissues. This multifaceted approach allowed us to systematically evaluate alterations in the tumor microenvironment, thus providing insights into the mechanisms behind the tumor-suppressive effects of MMW irradiation.

**Fig. 3.**
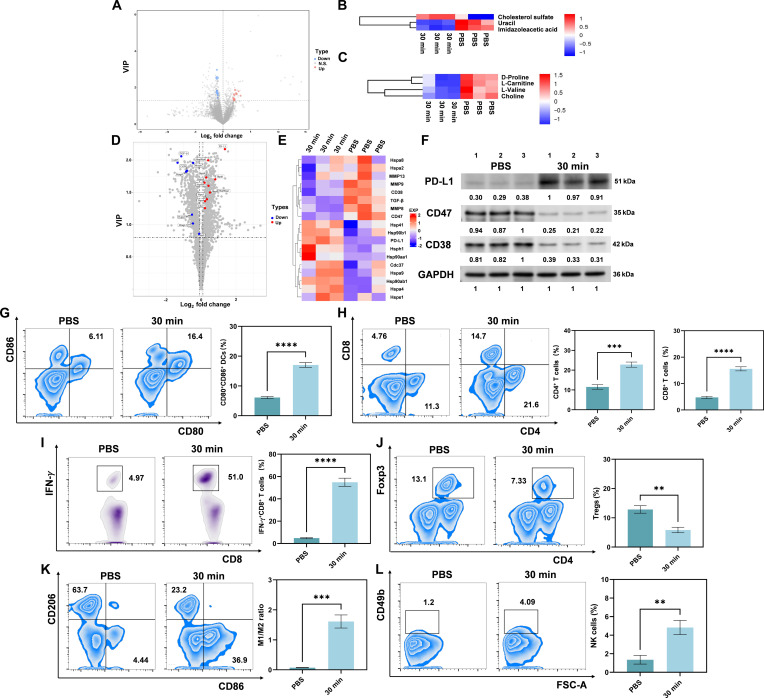
MMW remodeling of the tumor immune microenvironment. (A) Positive electro-metabolomic volcano plots of the MMW and control groups at 30 min (see Fig. S4 for negative electro-metabolomic volcano plots). (B) Heat map of the clustering analysis of the metabolite profiling systems of the 2 groups with negative ions and (C) with positive ions. (D) Volcano plot of the proteome analysis of the MMW group and the control group at 30 min. (E) Heat map of systematic cluster analysis of protein profiles of the 2 groups. (F) Western blotting plot of key proteins. (G) Proportion of mature DCs (CD45^+^CD11b^+^MHCII^+^CD80^+^CD86^+^) in the draining lymph nodes of mouse tumors detected by flow cytometry between the control and MMW-irradiated groups and statistical analysis. (H) Proportion of CD4^+^ T cells (CD45^+^CD3^+^CD4^+^) and CD8^+^ T cells (CD45^+^CD3^+^CD8^+^) in tumor tissues and statistical analysis. (I) Proportion of activated CD8^+^ T cells (CD45^+^CD3^+^CD8^+^IFN-γ^+^) in tumor tissues and statistical analysis. (J) Proportion of Tregs (CD45^+^CD3^+^CD4^+^Foxp3^+^) in 4T1 tumors and statistical analysis. (K) Proportion of M1-type (CD45^+^CD11b^+^F4/80^+^CD86^+^) and M2-type (CD45^+^CD11b^+^F4/80^+^CD206^+^) macrophages in tumor tissues and statistical analysis. (L) Proportion of NK cells (CD45^+^CD3^−^CD49b^+^) in tumor tissues and statistical analysis. Statistical significance was set as **P < 0.01, ***P < 0.001, and ****P < 0.0001. VIP, variable importance in projection; N.S., not significant; EXP, expression level; GAPDH, glyceraldehyde-3-phosphate dehydrogenase; Fox3p, forkhead box P3.

Metabolomic analysis showed that the levels of various amino acids and organic nitrogen compounds, including choline and carnitine, were significantly reduced in the tumor tissues of the 30-min MMW irradiation group compared to those in the control group (Fig. 3A to C and Fig. S4). Choline is an important component of phospholipids in the tumor cell membrane, and carnitine serves as a key carrier of long-chain fatty acids across the mitochondrial membrane. The reduction in these metabolites indicates an abnormal metabolic state within the tumors [50]. This finding suggests a potential mechanism by which MMWs may restrict tumor nutrient access. Furthermore, proteomic analysis showed that the expression levels of several proteins related to immune regulation were changed in tumor cells after MMW irradiation, with 8 related proteins up-regulated and 10 related proteins down-regulated (Fig. 3D and E). Among them, a decrease in the matrix metalloproteinase (MMP) family may impair tumor cell migration and invasion [51], while an increase in heat shock proteins (Hsp90ab1, Hsp90aa1, etc.) might promote the ability of tumor cells to resist various pressures, making them more vulnerable to MMW radiation therapy [52]. The Western blot results demonstrated that MMW induced the up-regulation of programmed cell death-ligand 1 (PD-L1) expression and the down-regulation of CD47 and CD38 expression (Fig. 3F). It is noteworthy that this study is among the first to report that MMW irradiation can significantly alter the tumor metabolic profile and immune microenvironment. These findings imply that MMW irradiation may remodel the local tumor immune microenvironment through multiple pathways, potentially leading to anti-tumor immune activation [30].

In further pursuit of validating this hypothesis, alterations in various immune effector cell populations within tumor tissues were assessed. Specifically, MMW irradiation significantly increased the proportion of mature dendritic cells (DCs) (Fig. 3G), suggesting a potential enhancement in antigen-presenting capacity that could contribute to the activation of tumor-specific T cells. Meanwhile, the infiltration of CD8^+^ T cells (Fig. 3H) and interferon-γ (IFN-γ)^+^CD8^+^ T cells (Fig. 3I), which are crucial tumor-killing effector cells, was also substantially increased. Moreover, a marked reduction in immunosuppressive regulatory T cells (Tregs) (Fig. 3J) and M2-type macrophages (Fig. 3K) was observed in the MMW-irradiated group, while the proportion of NK cells was increased (Fig. 3L). This series of changes implies that MMW irradiation can enhance the tumor immune microenvironment both quantitatively and qualitatively. Terminal deoxynucleotidyl transferase mediated dUTP nick-end labeling (TUNEL) and immunohistochemistry experiments further confirmed that under MMW irradiation, the apoptosis of cancer cells in the tumor microenvironment was increased, while their proliferation was inhibited (Fig. S5). Additionally, the activation status of CD4^+^ and CD8^+^ cells in tumor tissue was significantly enhanced (Fig. S6). This indicates that MMW irradiation may facilitate the immune-mediated elimination of tumor cells by activating immune cells within the tumor microenvironment. The results showed that 30-min MMW irradiation led to a significant remodeling of the tumor immune microenvironment. Collectively, these changes suggest that MMW irradiation can foster a less immunosuppressive and more immunostimulatory tumor microenvironment, which is associated with the observed inhibition of tumor growth.

The aforementioned results demonstrate that MMW irradiation induces significant remodeling of the tumor microenvironment through dual metabolic and immunological modulation. This is achieved via (a) alteration of tumor metabolism, as evidenced by reduced metabolite levels; (b) increased infiltration of anti-tumor immune effector cells; and (c) reduction of immunosuppressive cell populations.

### Interaction between MMWs and key immune proteins from the perspective of molecular dynamics

Studies above have demonstrated that MMW irradiation can down-regulate the levels of CD47, CD38, and TGF-β in the tumor microenvironment. This novel finding suggests that MMWs may remodel the tumor immune microenvironment, potentially by influencing the structure and function of these proteins. However, the molecular mechanism by which MMWs affect these proteins has not been elucidated [53]. To address this gap, a suite of molecular dynamics simulations were executed to elucidate the impact of MMW radiation on the structural integrity and functional characteristics of these 3 proteins.

**Fig. 4.**
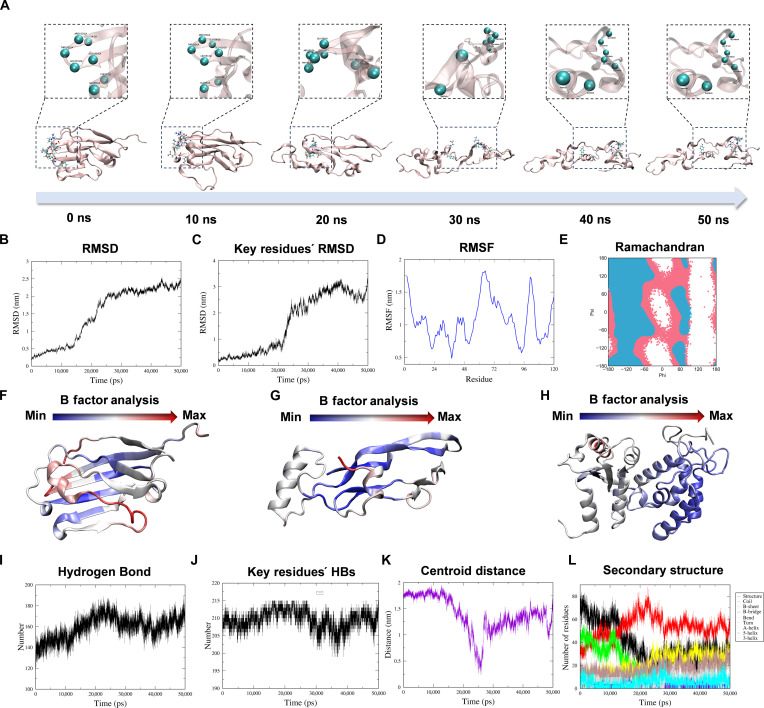
Molecular dynamics for simulating the 50-ns MMW irradiation of key proteins. (A) Molecular dynamics trajectory of the CD47 monomer in 50-ns simulation time. (B) CD47 monomer’s root mean square deviation (RMSD) within 50 ns. (C) RMSD of the key residues of the CD47 monomer. (D) Root mean square fluctuation (RMSF) values of all residues of the CD47 chain during the simulation. (E) Ramachandran diagram of the CD47 monomer. (F) Schematic diagram of CD47 protein B factor (blue: part with large structural change; red: part with small structural change). (G) Schematic diagram of TGF-β protein B factor. (H) Schematic diagram of CD38 protein B factor. (I) The number of hydrogen bonds in the CD47 monomer changes with the simulation time. (J) The number of hydrogen bonds between key residues and other residues in the CD47 monomer changes with the simulation time. (K) The distance between the centroid of the key residue in the CD47 monomer and the centroid of protein changes with time. (L) Secondary structure diagram of the CD47 monomer. HBs, hydrogen bonds.

Initial investigations focused on the CD47 protein monomer. The simulation results showed that MMW radiation induced significant conformational changes in CD47 (Fig. 4A), and the root mean square deviation (RMSD) curve exhibited a continuous upward trend (Fig. 4B), indicating that the overall conformation of CD47 gradually deviated from the initial state. Specifically, the RMSD trends of key residues (Glu35, Tyr37, Leu101, Thr102, and Arg103) were similar to those of protein RMSD but exhibited more pronounced fluctuations in the 20- to 30- and 40- to 50-ns periods (Fig. 4C). Root mean square fluctuation analysis further revealed that, in addition to the above key residues, the regions of residues 96 to 108 and 115 to 120 also exhibited significant fluctuations. This suggests that MMW radiation induced the substantial conformational changes throughout the CD47 protein (Fig. 4D). The Ramachandran diagram (Fig. 4E) demonstrated that the distribution of dihedral angles phi (*φ*) and psi (*ψ*) of CD47 protein changed markedly under MMW radiation. The B factor image showed that the regions containing the key residues Glu35, Tyr37, Leu101, Thr102, and Arg103 showed large structural fluctuations (Fig. 4F), suggesting that the structural dynamics of these key “pockets” involved in the interaction of CD47 with the antibodies and the SIRPα receptor were significantly altered [54].

Similar molecular dynamics simulations were conducted on TGF-β and CD38. TGF-β also showed conformational changes (Fig. 4G) upon MMW radiation (Fig. S7). Notably, the key H3 helix region underwent significant structural displacement or unraveling. This alteration is likely to hinder the dimerization of TGF-β, a process critical for its biological activity [55,56]. For the CD38 protein, MMW radiation resulted in significant alterations to its overall structure, particularly affecting crucial residues such as Glu226, Trp125, Trp189, Lys129, and Glu146. This structural instability may provide a molecular-level explanation for the prior observations of reduced CD38 expression following MMW exposure (Fig. 4H and Fig. S8) [57,58].

Again, upon in-depth scrutiny of these pivotal CD47 residues, it was revealed that the number of hydrogen bonds in the CD47 monomer (Fig. 4I), the hydrogen bonds between the key residues and other residues (Fig. 4J), the distance between the centers of the key residues and the center of the protein (Fig. 4K), and the secondary structure (Fig. 4L) were significantly altered, indicating the loss of structural stability and integrity within the key binding pockets. This may substantially reduce the binding affinity of CD47 to antibodies and SIRPα, which is consistent with the down-regulation of CD47 expression [59].

Together, these simulation results suggest a potential molecular mechanism by which MMW radiation could contribute to blocking tumor immune escape: through inducing conformational changes that destabilize critical immune-related proteins such as CD47, CD38, and TGF-β, potentially impairing their functions.

### MMW irradiation induces tumor cell damage and up-regulates immunogenicity

At the physiological level, MMWs have been shown to inhibit the growth and metastasis of solid tumors. To explore their direct cellular mechanism, we conducted cell culture experiments, which demonstrated that MMW irradiation could also directly affect tumor cells, inducing cellular damage and alterations in immunogenicity.

**Fig. 5.**
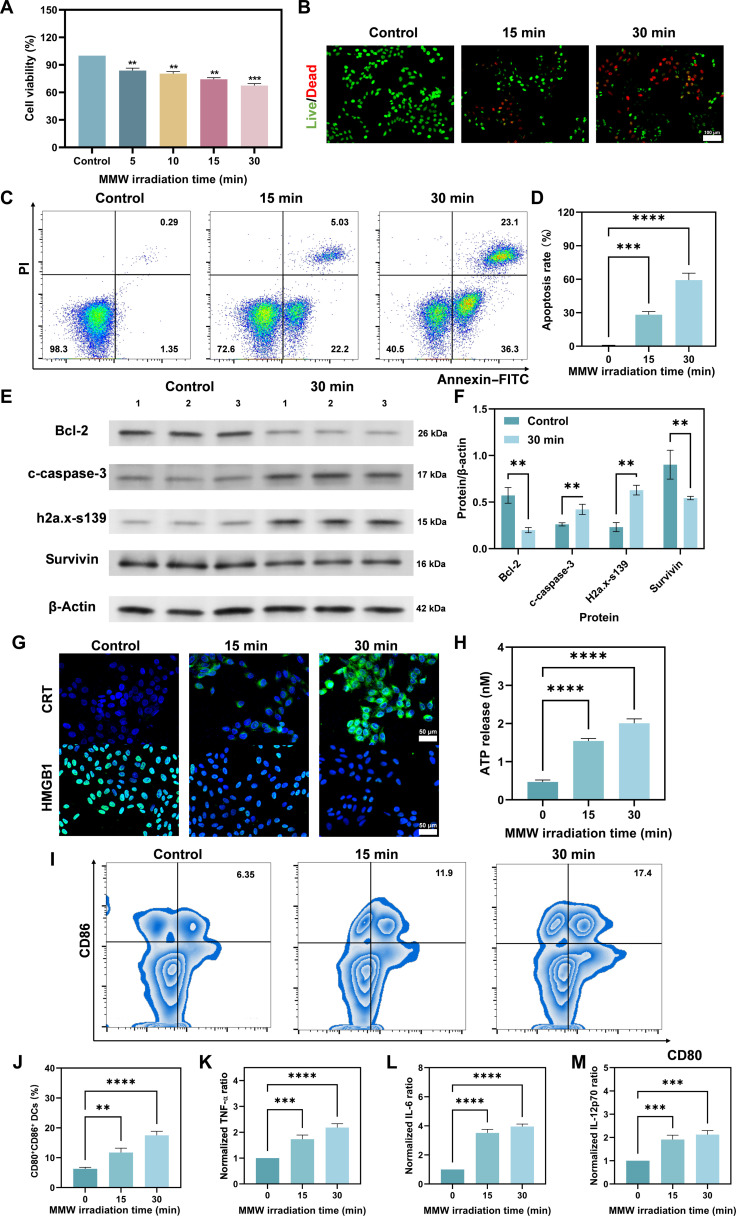
MMWs induce tumor cell damage and up-regulate immunogenicity. (A) Cell viability of 4T1 cells irradiated with MMWs. (B) Viability of 4T1 cells following MMW irradiation assessed by Live/Dead staining. Live cells (green, calcein-AM) and dead cells (red, propidium iodide [PI]) were visualized by laser confocal microscopy (scale bar: 100 μm). (C) Annexin V–fluorescein isothiocyanate (FITC)/PI detection of apoptosis results of 4T1 cells after 0-, 15-, and 30-min irradiation. (D) Quantitative analysis of (C). (E) Representative Western blot images of key proteins during MMW irradiation. (F) Quantitative analysis of (E). (G) The effects of different MMW irradiation times on the expression of CRT on the tumor surface and HMGB1 within tumor nuclei were detected using laser confocal microscopy (scale bar: 50 μm). (H) ATP fluorescence determination of the effect of different MMW irradiation times on ATP release from cells. (I) Flow cytometry of the proportion of mature DCs (CD80^+^CD86^+^) after stimulation with 4T1 tumor-conditioned media. (J) The effect of different MMW irradiation times on the percentage of mature DCs stimulated with 4T1 tumor-conditioned media detected by flow cytometry. (K) Enzyme-linked immunosorbent assay (ELISA) to detect the effects of different MMW irradiation times in supernatant on the release and expression of TNF-α, (L) IL-6, and (M) IL-12p70 content. Statistical significance was set as **P < 0.01, ***P < 0.001, and ****P < 0.0001.

Cell viability analysis showed a time-dependent decrease in the survival of 4T1 cells after MMW irradiation for different durations compared to that of the control group (Fig. 5A). Specifically, 30 min of irradiation reduced cell viability by approximately 40% (*P* < 0.001), indicating that MMWs may inhibit tumor cell proliferation [47]. It is worth noting that under the same irradiation conditions, the survival rate of healthy cells did not show significant changes (Fig. S9), suggesting that the inhibitory effect is selective for tumor cells over this specific normal cell type. In addition, laser confocal microscopy revealed a significant increase in cancer cell apoptosis with extended irradiation time (Fig. 5B and Fig. S10), suggesting that MMWs could induce programmed tumor cell death [60]. To further validate this finding, apoptosis was assessed via annexin V–fluorescein isothiocyanate/propidium iodide staining and flow cytometry. The results showed that 15- and 30-min irradiation increased the early apoptosis rate by nearly 18-fold and 55-fold, respectively (Fig. 5C and D). MMW irradiation prominently induced apoptosis in 4T1 tumor cells, suggesting potential alterations in apoptotic signaling pathways [61]. To test this, we examined the expression of anti-apoptotic proteins (Bcl-2 and survivin) and pro-apoptotic markers (cleaved caspase-3 and γ-h2a.x-s139) (Fig. 5E and F). The results showed that 30 min of MMW irradiation significantly down-regulated the expression of Bcl-2 and survivin while up-regulating the levels of c-caspase-3 and h2a.x-s139, compared to those of the control group. Among them, Bcl-2 family proteins inhibited programmed cell death in the mitochondrial pathway [62], while survivin, an inhibitor of apoptotic proteins, also inhibited caspase-induced apoptosis [63], and the down-regulation of these 2 proteins implied that 4T1 cells were weakened in their function to inhibit apoptosis. In contrast, activated c-caspase-3 is a key executor in initiating apoptosis [64], the production of h2a.x-s139 marks DNA damage and repair [65], and the up-regulation of these 2 proteins suggests that the DNA of 4T1 cells is damaged and that the cellular regulatory program is activated. In addition, modifications in the expression profiles of multiple MMPs, implicated in tumor invasion and metastasis, were discerned (Fig. S11) [66]. The results showed that MMW irradiation suppressed the expression of MMP-8, MMP-9, and MMP-13 to different degrees. This suggests that MMWs can directly induce tumor apoptosis and reduce its metastatic and invasive capabilities to some extent (Figs. S12 and S13).

Beyond the direct eradication of tumor cells, it has been discovered that MMW radiation enhances the immunogenicity of tumor cells through various mechanisms. With extended irradiation time, the surface exposure of calreticulin (CRT) on tumor cells and the release of high mobility group box-1 protein (HMGB1) into the culture supernatant were significantly increased (Fig. 5G and Figs. S14 to S16). CRT serves as a crucial phagocytic signal, promoting the phagocytosis of tumor cell cadavers by DCs [67]. HMGB1 is released following the death of tumor cells, which stimulates an inflammatory response and facilitates antigen presentation, thereby enhancing the tumor recognition capability of the immune system [68,69]. Consequently, MMWs regulate the release of these 2 proteins, increasing the possibility of tumor cells being recognized and eliminated by the immune system. Furthermore, a sustained elevation in adenosine triphosphate (ATP) release from tumor cells in correlation with extended periods of irradiation was observed (Fig. 5H). As extracellular ATP acts as a chemoattractant for DCs and other immune cells [70], this result suggests that MMW treatment may promote the recruitment of immune cells in vivo. To further investigate immunogenic effects, we co-cultured bone-marrow-derived DCs with irradiated 4T1 cells. Flow cytometry analysis showed that MMW irradiation significantly enhanced the maturation of DCs in this co-culture system compared to that in the control group (Fig. 5I and J). MMW irradiation also induced tumor cells to secrete higher levels of immune-activating factors, such as tumor necrosis factor-α (TNF-α), interleukin-6 (IL-6), and interleukin-12 (IL-12) (Fig. 5K to M), which could enhance the immune response of the body and thus clear the residual tumor more effectively [71].

Collectively, these in vitro results demonstrate that MMW irradiation directly induces immunogenic cell death in 4T1 cells, as characterized by the release of CRT, HMGB1, ATP, and pro-inflammatory cytokines. These changes significantly enhance the ability of tumor cells to activate DCs in a co-culture setting. Our findings suggest that these direct effects on tumor cells may contribute to the observed in vivo anti-tumor immune responses, ultimately improving immune-mediated tumor clearance.

### MMWs activate macrophages to produce an anti-tumor phenotype

**Fig. 6.**
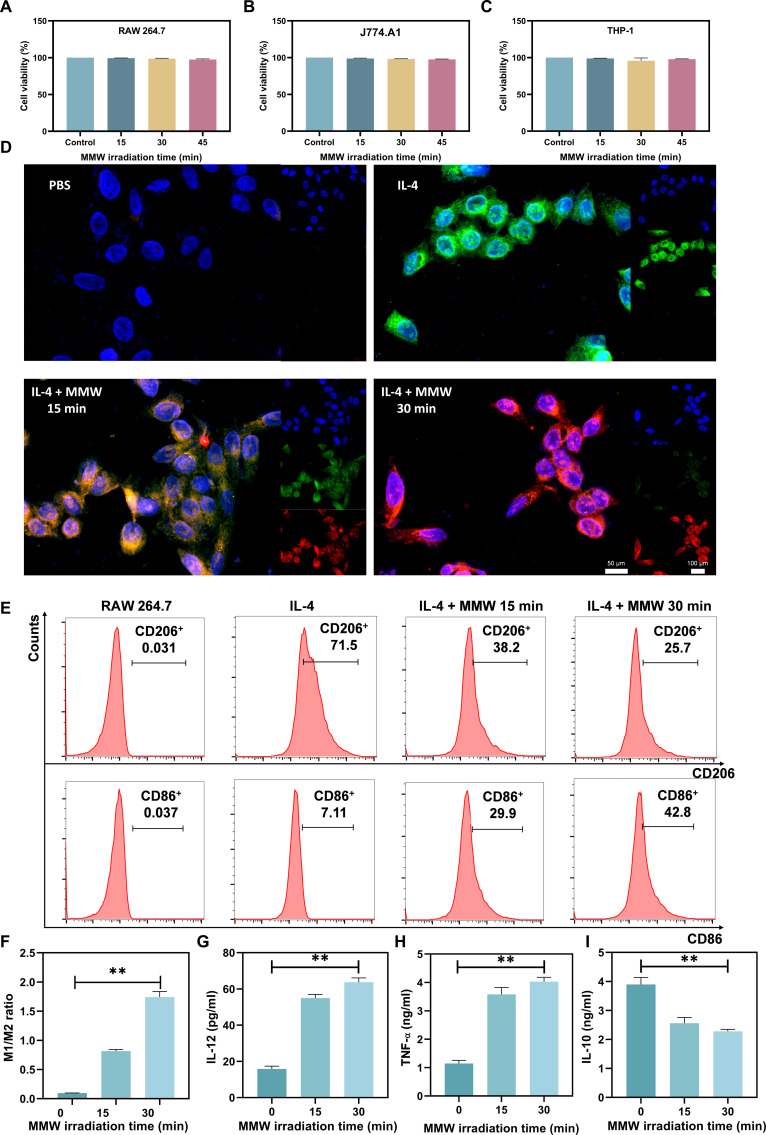
MMWs activate macrophages to produce an anti-tumor phenotype. Cellular activity of (A) RAW 264.7, (B) J774.A1, and (C) THP-1 macrophages irradiated by MMWs for different times. (D) The effects of IL-4 and different MMW irradiation time treatments on the expression of fluorescent-labeled protein CD206 and CD86 in RAW 264.7 macrophages detected by laser confocal scanning (scale bar: 50 or 100 μm). (E) The effects of IL-4 and different MMW irradiation treatments on the expression of CD86 and CD206 in RAW 264.7 macrophages detected by flow cytometry. (F) The effect of different MMW irradiation times on the M1/M2 differentiation ratio of mouse macrophages examined by flow cytometry analysis of specific surface markers. (G) ELISA to detect the effects of different MMW irradiation times in mouse macrophages on IL-12 secretion, (H) TNF-α secretion, and (I) IL-10 secretion. Statistical significance was set as **P < 0.01.

MMW irradiation has been proved to have an inhibitory effect on 4T1 tumors and tumor cells. To clarify the effects of MMWs on macrophage function, the initial step involved examining the viability of 3 macrophage cell lines: mouse RAW 264.7, J774.A1, and human THP-1. These cell lines were subjected to varying exposure times (0, 15, 30, and 45 min) of MMW radiation (Fig. 6A to C). The results showed that MMW irradiation did not significantly affect the survival of these macrophage cell lines under the experimental conditions, suggesting that MMWs do not produce significant toxic effects on macrophages.

Next, the impact of MMWs on the regulation of macrophage differentiation and function by interleukin-4 (IL-4) was investigated. IL-4 promotes macrophage differentiation toward the M2 type while inhibiting M1 differentiation [72,73]. The results of laser confocal scanning (Fig. 6D) and flow cytometry (Fig. 6E) consistently showed that MMW irradiation attenuated the effect of IL-4, inhibited the up-regulation of the M2 surface marker CD206, and promoted the expression of the M1 marker CD86. This polarization-shifting effect was further enhanced with prolonged MMW exposure. M2-type macrophages are key contributors to tumor progression and treatment resistance, as they promote tumor growth, inhibit immune responses, and diminish the effectiveness of immunotherapy [74,75]. These findings suggest that MMW irradiation induces a shift in macrophage polarization from the IL-4-induced M2 phenotype toward the M1 phenotype (Fig. 6F). We hypothesize that this shift may be mediated through the activation of M1-associated signaling pathways, such as nuclear factor kappa-B [44], potentially via alterations in cellular properties like membrane permeability. This MMW-induced polarization shift likely enhances the anti-tumor efficacy of macrophages.

Notably, the secretion of immune factors varies in accordance with the polarization shift of macrophages. Experimental results illustrate that prolonged MMW exposure promoted the secretion of pro-inflammatory cytokines IL-12 (Fig. 6G) and TNF-α (Fig. 6H) while inhibiting the secretion of the anti-inflammatory factor IL-10 (Fig. 6I). This cytokine profile is consistent with an enhanced ability to kill tumor cells. IL-12 and TNF-α, secreted by M1-type macrophages, are typical pro-inflammatory factors that activate and recruit additional immune cells to participate in tumor killing [76,77], while IL-10, secreted by M2-type macrophages, inhibits the inflammatory response and attenuates immune function [78]. Therefore, the MMW-induced alteration in the cytokine secretion profile (increased IL-12/TNF-α and decreased IL-10) likely contributes to the enhancement of macrophage anti-tumor function.

Collectively, the results indicate that MMW irradiation can directly act on macrophages, promoting their polarization toward an M1-like phenotype and altering their secretory profile. This suggests a potential mechanism through which MMW treatment might contribute to the reshaping of the tumor microenvironment and activation of anti-tumor immunity in vivo.

### MMW combined with α-PD-L1 treatment significantly inhibits tumor growth

The efficacy of monotherapy is often limited because tumor cells express immunosuppressive molecules, such as PD-L1, which allow them to evade immune surveillance and destruction. The proteomic analysis indicated that the level of PD-L1 increased following MMW irradiation, which would prevent further immunotherapy [79,80]. Therefore, we hypothesize that the combination of MMW irradiation and α-PD-L1 treatment may produce a synergistic effect.

**Fig. 7.**
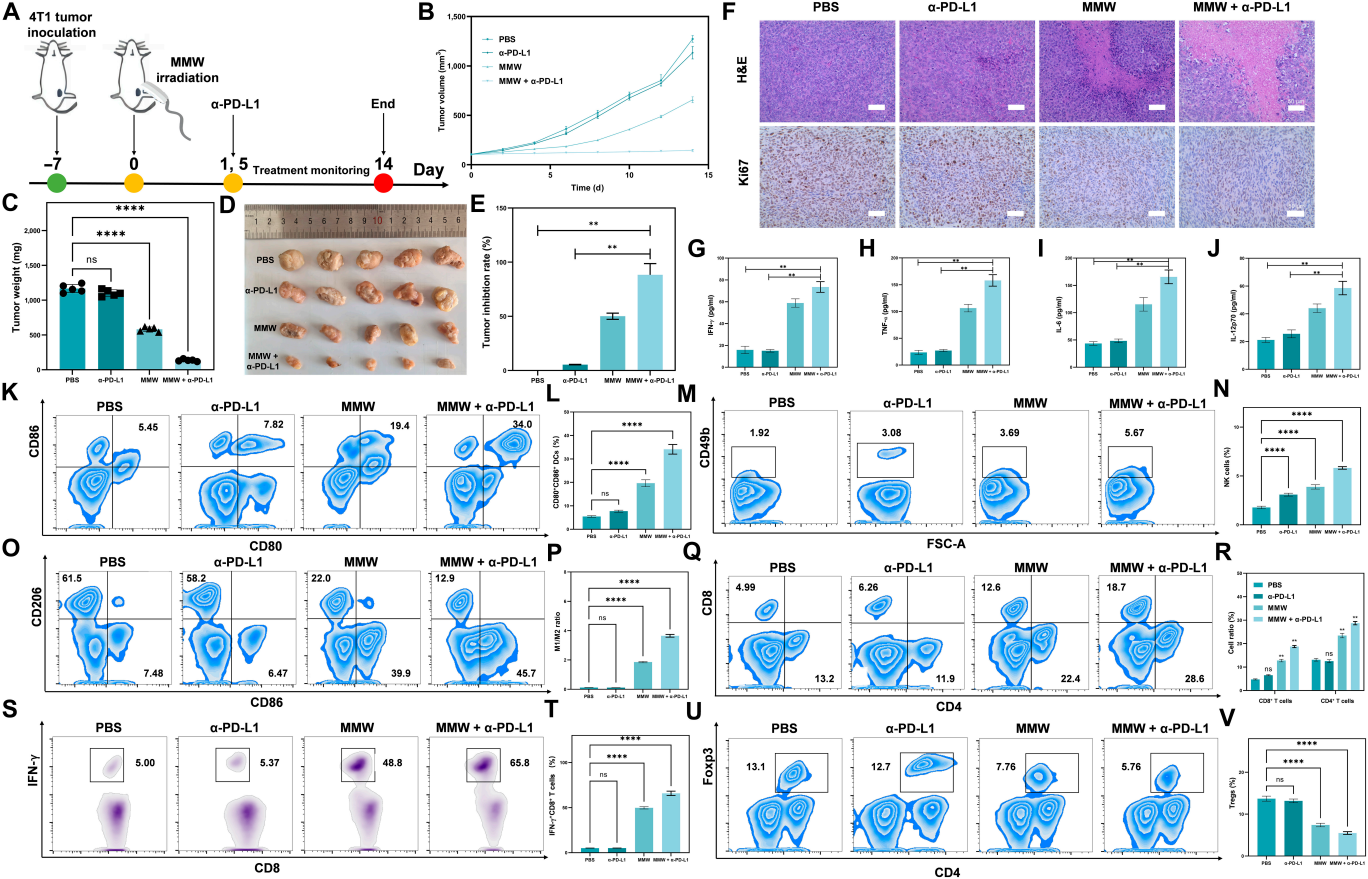
Effects of MMW therapy alone or in combination on the tumor growth and immune microenvironment of 4T1. (A) Schematic diagram of the treatment protocol involving the addition of anti-programmed cell death-ligand 1 (α-PD-L1) and MMWs after 4T1 tumor transplantation. (B) Changes in tumor volume in BALB/c mice bearing tumor (treated with PBS, α-PD-L1, MMWs, and MMW + α-PD-L1, respectively). (C) Tumor weight at the end of the experiment. (D) Photograph of tumor size change. (E) Tumor inhibition rate. (F) H&E and Ki67 staining of 4T1 tumor (scale bar: 50 μm). (G) Cytokine contents of IFN-γ, (H) TNF-α, (I) IL-6, and (J) IL-12p70 in 4T1 tumor-bearing BALB/c mice (treated with PBS, α-PD-L1, MMW and MMW + α-PD-L1, respectively). (K) Flow cytometry of the proportion of DCs (CD45^+^CD11b^+^MHCII^+^CD80^+^CD86^+^) in tumor drainage lymph nodes extracted from mice after PBS, α-PD-L1, MMW, and MMW + α-PD-L1 treatments (from left to right). (L) Histogram of the proportion of CD80^+^ and CD86^+^ double-positive DCs. (M) Flow cytometry analysis showed the proportion of NK cells (CD45^+^CD3^−^CD49b^+^) in 4T1 tumor tissue after treatment with PBS, α-PD-L1, MMW, and MMW + α-PD-L1, respectively (from left to right). (N) Proportional histogram of NK cells. (O) Flow cytometry of M1-type macrophages (CD45^+^CD11b^+^F4/80^+^CD86^+^) and M2-type macrophages (CD45^+^CD11b^+^F4/80^+^) isolated from 4T1 tumor tissue after PBS, α-PD-L1, MMW, and MMW + α-PD-L1 treatments, respectively. (P) Histogram of cell proportion in the M1/M2 phase. (Q) Flow cytometry analysis showing the proportion of CD8^+^ T cells (CD45^+^CD3^+^CD8^+^) and CD4^+^ T cells (CD45^+^CD3^+^CD4^+^) isolated from 4T1 tumor tissue after PBS, α-PD-L1, MMW, and MMW + α-PD-L1 treatments, respectively (from left to right). (R) Histogram of CD8^+^ or CD4^+^ single-positive cells. (S) Flow cytometry of the proportion of activated CD8^+^ T cells (CD45^+^CD3^+^CD8^+^IFN-γ^+^) isolated from 4T1 tumor tissue after PBS, α-PD-L1, MMW, and MMW + α-PD-L1 treatments, respectively. (T) Histogram of IFN-γ^+^ and CD8^+^ double-positive cells. (U) Flow cytometry of the proportion of Tregs (CD45^+^CD3^+^CD4^+^Foxp3^+^) isolated from 4T1 tumor tissue after PBS, α-PD-L1, MMW, and MMW + α-PD-L1 treatments (from left to right). (V) Proportional histogram of Tregs. Statistical significance was set as ns, no significant difference; **P < 0.01; and ****P < 0.0001.

To investigate the potential synergistic effect of MMW combined with α-PD-L1 treatment, a 4T1 breast cancer mouse model was employed to assess the combined anti-tumor efficacy of MMW irradiation and α-PD-L1 treatment (Fig. 7A). Initially, the in vivo safety of MMW irradiation was evaluated. Notably, MMW irradiation for 30 min did not impact the serum indices of liver and kidney function (Fig. S17) or the histomorphology of major organs, including the heart, liver, spleen, lungs, kidneys, and brain (Fig. S18) in normal mice.

The results showed that the combined therapy group exhibited the slowest tumor growth rate, the lowest tumor weight, and a tumor inhibition rate as high as 85% (Fig. 7B to E). Histopathological analysis, including TUNEL, H&E, and Ki67 staining results (Fig. S19 and Fig. 7F), further corroborated the effectiveness of the combined therapy in reducing proliferating cells and inhibiting tumor growth. Compared to the control group and the single-treatment groups, the combined treatment did not significantly reduce the body weight of the mice (Fig. S20). These results preliminarily suggest that the combination of MMW and α-PD-L1 is safe and demonstrates a strong anti-tumor effect, potentially indicative of a synergistic interaction.

To thoroughly investigate the synergistic mechanisms of the 2 therapies, we scrutinized key molecular and cellular alterations within the tumor microenvironment. Inflammatory factors such as IFN-γ, TNF-α, IL-6, and IL-12 have been shown to enhance the body’s anti-tumor response. The assay revealed that the expression levels of these cytokines were significantly elevated in the combination therapy group (Fig. 7G to J). This finding suggests that MMW combined with α-PD-L1 treatment may exert anti-tumor effects by activating immune molecules within the tumor microenvironment. Further analysis indicated that the proportion of DCs, especially mature DCs (CD80^+^CD86^+^), was significantly increased in the combination treatment group (Fig. 7K and L). As antigen-presenting cells, their increase is likely to stimulate an adaptive immune response against the tumor [81]. Concurrently, the number of NK cells, which directly target and kill tumor cells, also increased significantly (Fig. 7M and N). Additionally, the proportion of pro-inflammatory M1-type macrophages rose (Fig. 7O and P), contributing to the remodeling of the tumor microenvironment and fostering a local immune state that is more conducive to tumor eradication [82].

At the tumor T cell level, there was a significant increase in CD4^+^ cells and CD8^+^ cells (Fig. 7Q and R), as well as activated IFN-γ^+^CD8^+^ T cells (Fig. 7S and T) in the combination therapy group. These cells are directly involved in the specific elimination of tumor cells. Notably, the proportion of immunosuppressive Tregs (CD4^+^Foxp3^+^), on the other hand, decreased significantly (Fig. 7U and V), which is beneficial for enhancing overall anti-tumor immunity. In addition, the results of immunofluorescence staining (Fig. S21) showed that CD4^+^ and CD8^+^ T cells in tumor tissues from the MMW treatment groups, particularly in the combined treatment group, were significantly elevated. This suggests that the combination treatment can effectively activate the anti-tumor cellular immune response and enhance tumor cell destruction in this model.

The results confirm that MMW therapy can markedly remodel the tumor microenvironment by activating various immune effector cells while inhibiting immunosuppressive cells. Additionally, the combined therapy produced a greater effect than either monotherapy.

### MMWs induce systemic anti-tumor immune response and inhibits distal metastasis

**Fig. 8.**
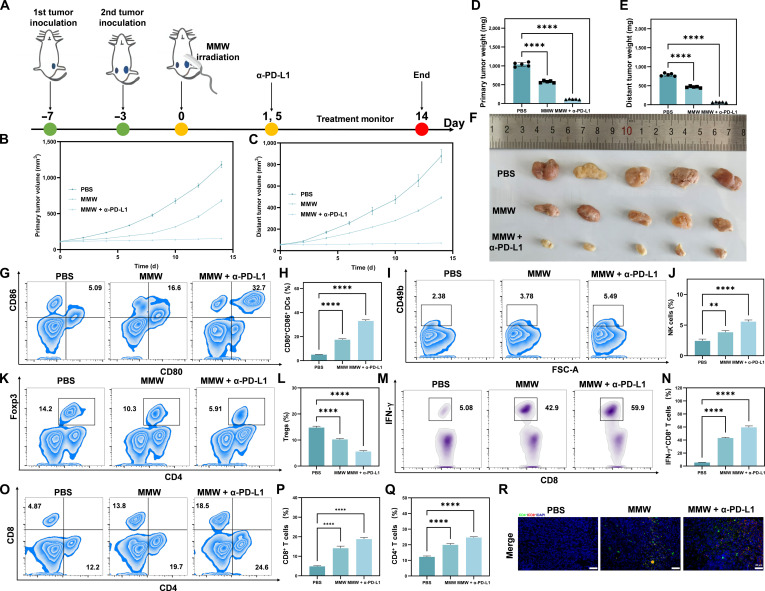
Bilateral 4T1 tumor response to MMW and α-PD-L1 treatment and immune cell analysis. (A) Schematic diagram of the treatment time of adding α-PD-L1 and MMWs after bilateral transplantation of 4T1 tumor. (B) Changes in primary tumor volume in 4T1 tumor-bearing BALB/c mice (treated with PBS, MMW, and MMW + α-PD-L1, respectively). (C) Changes in distant tumor volume in 4T1 tumor-bearing BALB/c mice (treated with PBS, MMW, and MMW + α-PD-L1, respectively). (D) Primary tumor weight at the end of the experiment. (E) Distant tumor weight at the end of the experiment. (F) Photograph of distant tumor size change. (G) Flow cytometry of the proportion of DCs (CD45^+^CD11b^+^MHCII^+^CD80^+^CD86^+^) in distant tumor-draining lymph nodes of mice treated with PBS, MMW, and MMW + α-PD-L1 (from left to right). (H) Histogram of the proportion of CD80^+^ and CD86^+^ double-positive DCs. (I) Flow cytometry of the proportion of NK cells (CD45^+^CD3^−^CD49b^+^) in distant 4T1 tumor tissues (from left to right) after treatment with PBS, MMW, and MMW + α-PD-L1, respectively. (J) Histogram of the proportion of NK cells. (K) Flow cytometry of the proportion of Tregs (CD45^+^CD3^+^CD4^+^Foxp3^+^) isolated from distant 4T1 tumor tissues after PBS, MMW, and MMW + α-PD-L1 treatments (from left to right). (L) Histogram of the proportion of Tregs. (M) Flow cytometry of the proportion of activated CD8^+^ T cells (CD45^+^CD3^+^CD8^+^IFN-γ^+^) isolated from distant 4T1 tumor tissues after treatment with PBS, MMW, and MMW + α-PD-L1, respectively. (N) Histogram of IFN-γ^+^ and CD8^+^ double-positive cells. (O) Flow cytometry of the proportions of CD8^+^ T cells (CD45^+^CD3^+^CD8^+^) and CD4^+^ T cells (CD45^+^CD3^+^CD4^+^) isolated from distant 4T1 tumor tissues (from left to right) after PBS, MMW, and MMW + α-PD-L1 treatments, respectively. (P) Histogram of CD8^+^ single-positive T cells. (Q) Histogram of CD4^+^ single-positive T cells. (R) The immunofluorescence staining results of CD4^+^, CD8^+^, and 4′,6-diamidino-2-phenylindole (DAPI) in the distant 4T1 tumor tissue of mice show the Merge diagram (scale bar: 20 μm). Statistical significance was set as **P < 0.01 and ****P < 0.0001.

As elaborated in the preceding section, our results demonstrate that MMW irradiation can synergize with α-PD-L1 antibody therapy to significantly enhance the inhibitory effect on primary tumors. The potential of MMW irradiation, in combination with this therapy, to extend its effects to distal metastases and elicit a systemic anti-tumor immune response was investigated. In this study, a bilateral 4T1 breast cancer mouse model was established. As shown in Fig. 8A, after the bilateral subcutaneous transplantation of tumor cells, the primary tumors on one flank were treated with PBS, MMW irradiation, or a combination of MMW and α-PD-L1.

The results showed that MMW irradiation alone significantly inhibited the growth of both primary and distal tumors simultaneously. When combined with α-PD-L1, the tumor-suppressive effect was further enhanced. This enhancement was evident not only in the substantial reduction of tumor volume (Fig. 8B and C) and tumor weight (Fig. 8D and E) but also in the photographs from the experimental recordings (Fig. 8F). Statistical analysis revealed that the bilateral tumor inhibition rate for MMWs alone was approximately 50%, which was significantly lower than around 95% for the combined treatment (Fig. S22).

To explore the potential mechanism underlying MMW-induced distal tumor suppression, a comprehensive examination of diverse immune cell populations within the tumor microenvironment was conducted. The results showed that MMW irradiation, both alone and in combination with other treatments, significantly increased the proportion of DCs in the distal tumor-draining lymph nodes (Fig. 8G and H). This increase suggests enhanced potential for presenting tumor-associated antigens to T cells and activating a specific anti-tumor immune response of the body, as supported by previous literature [83]. Concurrently, the infiltration of NK cells in the distal tumor tissues was markedly enhanced (Fig. 8I and J), which is consistent with an enhancement of direct cytotoxicity against tumor cells. Regarding T cells, MMW treatment selectively reduced the proportion of Tregs in distal tumors (Fig. 8K and L). Furthermore, MMW irradiation, whether administered alone or as part of combination therapy, promoted the activation of CD8^+^ T cells (Fig. 8M and N) and enhanced the infiltration of CD8^+^ cytotoxic T cells and CD4^+^ helper T cells into distal tumors (Fig. 8O to Q). Consistent with these findings, immunostaining analysis produced similar results (Fig. 8R and Fig. S23), collectively indicating that MMWs modulate the tumor immune microenvironment and elicit a robust anti-tumor immune response. Together, these data suggest that MMWs may remodel the immune landscape within the tumor microenvironment toward a phenotype more favorable for tumor eradication, which could contribute to the observed systemic anti-tumor immune response and inhibition of distal metastasis growth.

Further histopathological analysis corroborated the previously mentioned findings. Following MMW irradiation or combined treatment, the number of Ki67-positive proliferating cells was significantly reduced in distal tumor tissues, while the number of TUNEL-positive apoptotic cells was significantly increased (Fig. S24), indicating that tumor growth was inhibited. Simultaneously, the absence of significant body weight loss in the mice (Fig. S25) suggests the absence of major systemic toxicity under these treatment conditions.

This discovery reveals the mechanism by which MMWs can induce a systemic anti-tumor immune response and inhibit the proliferation of distant metastases. Furthermore, our data support the rationale for combining MMWs with immune checkpoint inhibitors, such as α-PD-L1, to further enhance this anti-tumor immune response.

### MMW irradiation coupled with α-PD-L1 treatment induces anti-tumor immune memory and suppresses tumor relapse

Tumor recurrence remains a significant clinical challenge across various treatment strategies for tumor control [84]. While immunotherapy can inhibit tumor recurrence by activating tumor-specific T cells, T cell exhaustion caused by the immunosuppressive tumor microenvironment severely hinders the differentiation of effector T cells into memory phenotypes, thus obstructing the establishment of long-term immunological memory [85]. Based on these considerations, we hypothesized that MMW irradiation, particularly in combination with α-PD-L1 treatment, could induce a robust anti-tumor immune memory to suppress tumor recurrence.

**Fig. 9.**
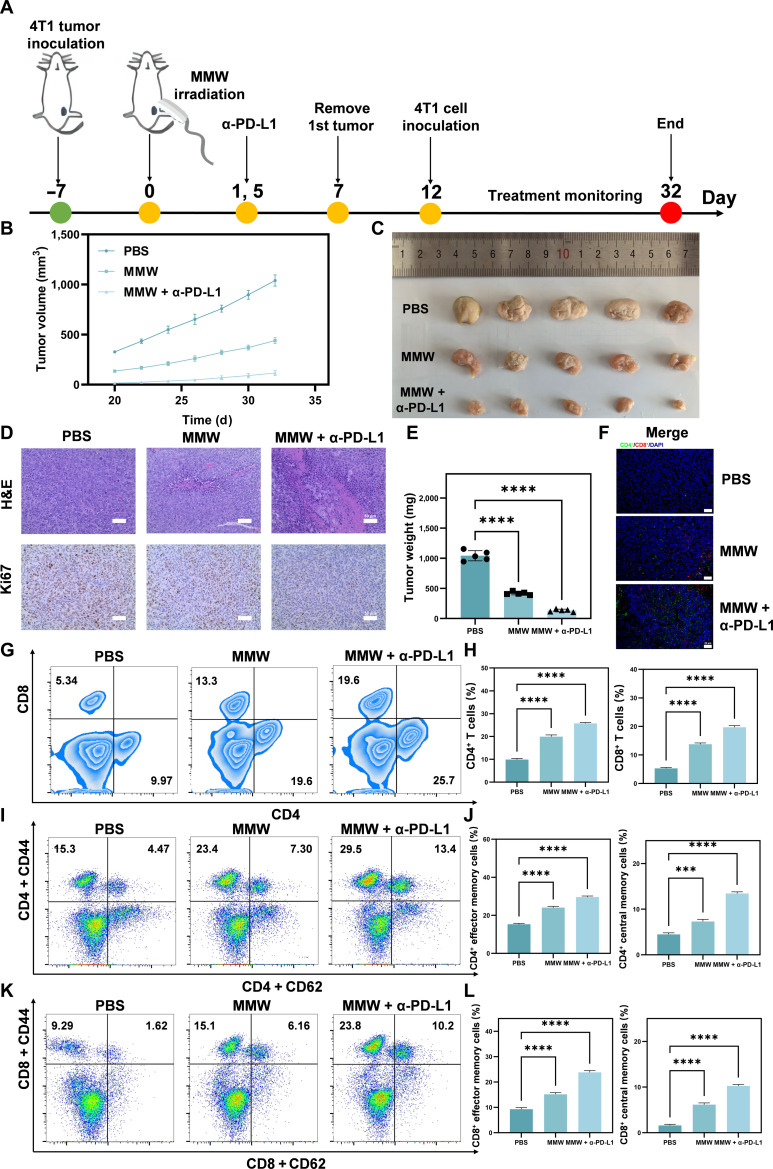
Secondary 4T1 tumor growth and immune memory response to MMW and α-PD-L1 treatment. (A) BALB/c mice inoculated with 4T1 tumors were treated with α-PD-L1 and MMWs, followed by 4T1 cells’ reinoculation after tumor resection. (B) Changes in tumor volume in BALB/c mice inoculated with 4T1 cells for the second time (treated with PBS, MMW, and MMW + α-PD-L1, respectively). (C) Photos of changes in tumor size of the second inoculation. (D) The H&E and Ki67 staining patterns of the 4T1 tumor for the second inoculation (scale bar: 50 μm). (E) The weight of the second tumor inoculation at the end of the experiment. (F) Merge diagram in the immunofluorescence staining results for CD4^+^, CD8^+^, and DAPI in 4T1 tumor tissue from the second inoculation (scale bar: 20 μm). (G) The ratio of CD8^+^ T cells and CD4^+^ T cells in mouse spleen T lymphocytes under different treatment conditions (PBS, MMW, and MMW + α-PD-L1) detected by flow cytometry. (H) Histogram of CD4^+^ and CD8^+^ T cells. (I) The proportion of effector memory T (TEM) (CD45^+^CD3^+^CD4^+^CD44^+^CD62^−^) and central memory T (TCM) cells (CD45^+^CD3^+^CD4^+^CD44^+^CD62^+^) in CD4^+^ T cells in mouse spleen T lymphocytes under different treatment conditions (PBS, MMW, and MMW + α-PD-L1) detected by flow cytometry. (J) Histogram of the percentage of TEM and TCM cells in CD4^+^ T cells. (K) Flow cytometry indicating the ratio of TEM (CD45^+^CD3^+^CD8^+^CD44^+^CD62^−^) and TCM cells (CD45^+^CD3^+^CD8^+^CD44^+^CD62^+^) in CD8^+^ T cells in mouse spleen T lymphocytes under different treatment conditions (PBS, MMW, and MMW + α-PD-L1). (L) Histogram of the percentage of TEM and TCM cells in CD8^+^ T cells. Statistical significance was set as ***P < 0.001 and ****P < 0.0001.

To replicate the clinical scenario in which tumors recur after surgical removal, an in vivo model was established that involved tumor resection followed by secondary tumor inoculation (Fig. 9A). The results shows that MMW treatment significantly delayed the growth of secondary tumors (Fig. 9B and C). Histological analyses demonstrated a reduction in tumor cell density and proliferation rate (Fig. 9D, H&E and Ki67 staining), along with increased apoptosis (Fig. S26, TUNEL staining) in the relapsed tumors from the MMW and MMW + α-PD-L1 treatment groups compared to those in the controls. Together, these findings, including the reduced tumor weight (Fig. 9E), demonstrate the efficacy of MMW in suppressing tumor relapse.

Immune memory primarily depends on the activation and proliferation of central and effector T lymphocytes [86,87]. To elucidate the mechanisms underlying MMW-induced immune memory, this investigation focused on 2 crucial populations of memory cells in treated mice. First, the CD4^+^ and CD8^+^ T lymphocytes were analyzed, as these effector cells mediate anti-tumor cellular immunity. Immunostaining analysis (Fig. 9F and Fig. S27) and flow cytometry (Fig. 9G and H) demonstrated that the number and activity of CD4^+^ and CD8^+^ T lymphocytes increased in the MMW group, with a particularly significant increase observed in the combined treatment group. Their elevated numbers and functionality are associated with an enhanced ability of the host to mount an anti-tumor immune response [88,89]. Additionally, an evaluation was conducted on effector memory T cells and central memory T cells (Fig. 9I to L), 2 key subsets that underpin immunological memory. Effector memory T cells are critical effectors that execute anti-tumor functions, while central memory T cells are long-lived memory cells capable of rapidly reactivating and expanding upon tumor recurrence, serving as the central hub of anti-tumor immune memory [90]. Notably, both effector and central memory populations were significantly increased in the MMW + α-PD-L1 and MMW treatment groups, indicating enhanced activation and proliferation of these essential anti-tumor immune cells. This enhanced activation is consistent with the formation of sustained immune memory and is likely a contributing factor to the observed suppression of tumor relapse.

These results demonstrate that MMW irradiation treatment can impede tumor recurrence by inducing tumor-specific T cell immune memory. Furthermore, the combination with the immune checkpoint inhibitor α-PD-L1 has the potential to significantly enhance these effects.

### MMW irradiation and the α-PD-L1 immune checkpoint inhibitor synergize to inhibit tumor growth and induce persistent immune memory in the CT26 colon cancer model

It has been demonstrated that MMW irradiation can produce synergistic anti-tumor effects when combined with α-PD-L1 immune checkpoint inhibitors. To further investigate the clinical applicability of this combined approach in different solid tumor contexts, we shifted our focus to an in-depth examination of the CT26 colon cancer mouse xenograft model. This effort aimed to evaluate the therapeutic efficacy of this novel strategy in other “cold tumor” environments.

**Fig. 10.**
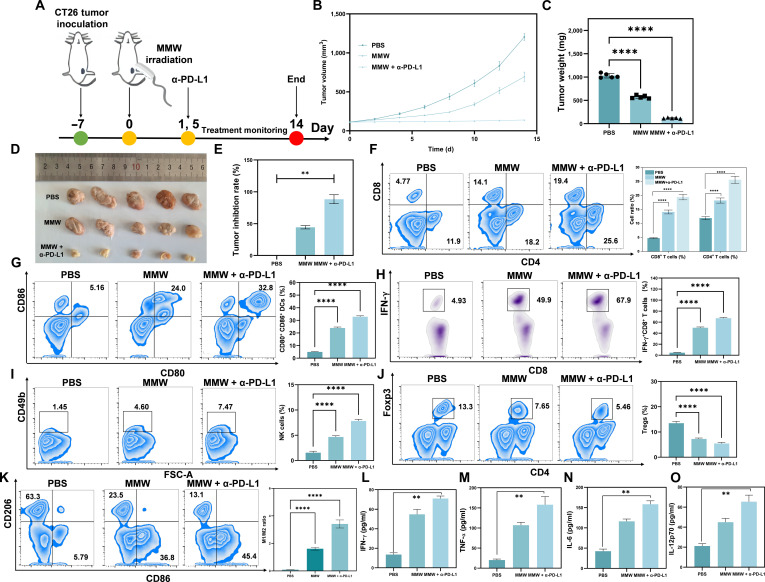
Effects of MMW therapy alone or in combination on tumor growth and immune indices of mice with CT26 “cold tumor”. (A) Schematic diagram of treatment time with α-PD-L1 and MMWs after CT26 tumor transplantation. (B) Changes in tumor volume in BALB/c mice bearing CT26 (treated with PBS, MMW, and MMW + α-PD-L1, respectively) and (C) tumor weight at the end of the experiment. (D) Photograph of tumor size change and (E) tumor inhibition rate. (F) Flow cytometry analysis showed the proportion of CD8^+^ T cells (CD45^+^CD3^+^CD8^+^) and CD4^+^ T cells (CD45^+^CD3^+^CD4^+^) isolated from tumor tissues of mice with statistical analysis. (G) The proportion of DCs (CD45^+^CD11b^+^MHCII^+^CD80^+^CD86^+^) in tumor drainage lymph nodes extracted from mice after PBS treatment (left), MMW irradiation (middle), and MMW + α-PD-L1 treatment (right) with statistical analysis. (H) The proportion of activated CD8^+^ T cells (CD45^+^CD3^+^CD8^+^IFN-γ^+^) isolated from CT26 tumor tissue and statistical analysis. (I) The proportion of NK cells (CD45^+^CD3^−^CD49b^+^) in CT26 tumor tissue and statistical analysis. (J) The proportion of Tregs (CD45^+^CD3^+^CD4^+^Foxp3^+^) in CT26 tumors and statistical analysis. (K) The ratio of M1-type macrophages (CD45^+^CD11b^+^F4/80^+^CD86^+^) and M2-type macrophages (CD45^+^CD11b^+^F4/80^+^CD206^+^) isolated from CT26 tumor tissue and statistical analysis. (L) Expressions in CT26 tumors of IFN-γ, (M) TNF-α, (N) IL-6, and (O) IL-12p70 obtained by ELISA. Statistical significance was set as **P < 0.01 and ****P< 0.0001.

The CT26 colon cancer mouse transplantation tumor model was established, and various treatments were administered to the PBS control group, the MMW irradiation group (MMW alone), and the MMW combined with α-PD-L1 treatment group (MMW + α-PD-L1) according to the timeline illustrated in Fig. 10A. The tumor growth curves (Fig. 10B) indicate that compared to the control group, the MMW group significantly inhibited tumor enlargement, with an even greater inhibition observed in the MMW + α-PD-L1 group. This finding was further corroborated by the final tumor weight (Fig. 10C) and volume data (Fig. 10D). Additional analysis revealed that the tumor inhibition rate of the MMW + α-PD-L1 group was 90% (Fig. 10E), which was significantly higher than those of the other groups.

To elucidate the underlying molecular mechanisms underlying the anti-tumor effects, a comprehensive cellular-level analysis was conducted. The in vitro results demonstrated that MMW irradiation could directly inhibit the activity of CT26 cells (Fig. S28). Further histological analysis revealed a significant number of apoptotic cells present in the CT26 tumor tissues after MMW treatment. Notably, the degree of apoptosis was more pronounced in the MMW + α-PD-L1 group, and the number of proliferating tumor cells (Ki67-positive) were significantly reduced (Fig. S29). It is important to note that the body weight of the mice remained stable throughout the experimental process (Fig. S30), suggesting that the treatment regimens were well tolerated.

Given that immune remodeling is a critical mechanism for tumor inhibition [91], we hypothesized that the observed effects might be mediated by changes in the tumor immune microenvironment. Flow cytometry analysis revealed a significantly enhanced activation of both CD4^+^ and CD8^+^ T cells in tumor tissues from the combination therapy group and the MMW treatment group (Fig. 10F). Similar results were obtained from immunostaining analysis (Fig. S31). MMW irradiation also significantly increased the infiltration of DCs in tumor-draining lymph nodes (Fig. 10G), which was associated with the enhanced activation of both CD8^+^ T cells (Fig. 10H) and NK cells (Fig. 10I), as well as a concurrent reduction in the populations of immunosuppressive Tregs (Fig. 10J). Furthermore, the MMW + α-PD-L1 combination therapy promoted polarization toward anti-tumor M1-type macrophages (Fig. 10K), which secreted elevated levels of effector molecules, including IFN-γ, TNF-α, IL-6, and IL-12p70 (Fig. 10L to O). Collectively, these changes are consistent with the establishment of an immunostimulatory microenvironment conducive to tumor cell clearance. Through these synergistic mechanisms, the MMW + α-PD-L1 group demonstrated the most potent tumor growth inhibition among all treatment regimens.

The results demonstrated that MMW irradiation can effectively inhibit tumor growth in the CT26 “cold tumor” model. Additionally, it can reshape the local and regional anti-tumor immune response, as evidenced by enhanced T cell activation and altered immune cell populations within the tumor microenvironment and draining lymph nodes. Furthermore, the administration of α-PD-L1 will enhance these effects.

## Discussion

This study suggests the potential of MMWs not only as an adjuvant intervention but also as a promising modality for remodeling the tumor microenvironment and synergistically enhancing the efficacy of immunotherapy. Building on our findings, we explore the significance and broader implications of MMW-based immunotherapy below, structured to address key mechanistic insights, clinical relevance, and limitations.

### Mechanistic implications

The selection of MMWs, as opposed to other physical modalities such as microwaves, near-infrared light, or ultrasound, is fundamentally based on their unique biophysical properties and interaction mechanisms with biological systems [92]. Notably, at the low energy densities employed in this study (≤10 mW/cm^2^), the observed effects are consistent with nonthermal, nonionizing mechanisms, as significant heating was not detected. This distinguishes them from modalities like microwaves, which primarily produce thermal effects, and near-infrared light, which often relies on photothermal or photodynamic effects that require photosensitizers, potentially causing collateral damage. Although ultrasound is also nonionizing, it primarily exerts its effects through mechanical (cavitation) and thermal mechanisms. In contrast, the multifaceted mechanism of MMWs warrants further investigation. Beyond directly inducing apoptosis in cancer cells, MMWs convert dying cells into immunogenic antigens and signaling sources, demonstrating both dual direct cytotoxic and indirect immunomodulatory effects. Importantly, our study reveals that MMWs induce conformational modulation of key immunoregulatory proteins (CD47, CD38, and TGF-β), providing a biophysical basis for their immune remodeling capacity. However, rigorous validation through biophysical techniques (e.g., nuclear magnetic resonance and crystallography) is necessary to elucidate atomic-level structural changes. Furthermore, the differential impact of MMWs on immune subpopulations (e.g., DCs and T cells) requires systematic characterization to clarify cellular targeting specificity.

### Clinical translation potential

The observed robust T cell activation and cytokine secretion suggest the potential for MMW-induced durable anti-tumor immune memory, which would represent a cornerstone for long-term disease control and relapse prevention. Importantly, our data demonstrate that MMW monotherapy exhibits significant anti-tumor efficacy and immune remodeling capabilities in “cold tumor” models (4T1 and CT26), surpassing the response observed with α-PD-L1 monotherapy in these settings. Furthermore, the synergistic combination of MMWs with α-PD-L1 achieved high tumor suppression rates (up to 90%) and durable immune memory compared to either modality alone, effectively overcoming the primary limitations of immune checkpoint inhibitor monotherapy (e.g., poor response in “cold tumors” and acquired resistance). This potentiation effect positions MMWs as a highly promising complementary modality with the potential to enhance the efficacy of established immunotherapies like α-PD-L1. This effect, combined with their favorable safety profile, underscores their clinical promise. Future work should prioritize (a) spatiotemporal mapping of immune cell training dynamics post-MMW exposure [93] and (b) strategic combinations with emerging immunotherapies (e.g., chimeric antigen receptor T-cell and neoantigen vaccines) to amplify synergistic efficacy.

### Limitations and future directions

The current study has several limitations that should be addressed in future work: the need for in-depth validation of protein conformational changes through structural biology approaches, the requirement for comprehensive parameterization scans to identify the optimal MMW frequency and power density for maximizing therapeutic efficacy while ensuring safety, unresolved heterogeneity in immune cell subtype responses, and pending in-human safety and efficacy trials. Addressing these issues will accelerate clinical deployment and optimize combinatorial regimens.

## Conclusion

In this study, we systematically evaluated the anti-tumor effects of MMWs as a monotherapy and in combination with the immune checkpoint inhibitor α-PD-L1. The results showed that MMWs induced apoptosis in cancer cells and was associated with a robust anti-tumor immune response, suggesting enhanced tumor immunogenicity. Mechanistic analysis indicated that MMW irradiation was associated with alterations in tumor cell metabolism, protein, and immune-related pathways, which correlated with the inhibition of tumor cell growth and a reduction of immunosuppression. In models of 4T1 breast cancer and CT26 colorectal cancer, MMWs, either alone or in combination with α-PD-L1, exhibited significant tumor growth inhibition and induced immune activation profiles consistent with the development of long-term immune memory. Mechanistic analysis indicated that the synergistic anti-tumor effects of MMWs, observed at both primary and metastatic sites, are mediated by MMW-induced remodeling of the tumor immune microenvironment. This remodeling includes enhanced antigen-presenting cell function and activation of cytotoxic T cells and NK cells, which were further potentiated by α-PD-L1 treatment. These findings in 2 distinct “cold tumor” models (4T1 and CT26) support the potential of MMWs as a personalized strategy for tumors unresponsive to immunotherapy while underscoring the need for validation in broader tumor types.

## Ethical Approval

All animal experiments were carried out after approval by the ethical committee for animal care of the Beijing Institute of Technology, China (Permit No. SYXK Jing 2017-0031).

## Data Availability

Data is available upon request.

## Supplementary Materials

Supplementary 1 Graphical Abstract Supplementary Materials and Methods Gromacs Files Information Figs. S1 to S33
