# Supplementary material for: Reviving Dormant Immunity: Millimeter Waves Reprogram the Immunosuppressive Microenvironment to Potentiate Immunotherapy without Obvious Side Effects
Source: Cyborg Bionic Syst. 2025 Dec 10;6:0468. doi: 10.34133/cbsystems.0468 (PMC12695134; doi:10.34133/cbsystems.0468)
Supplement: Supplementary 1 — Graphical Abstract Supplementary Materials and Methods Gromacs Files Information Figs. S1 to S33 [file cbsystems.0468.f1.zip › Supplementary Materials_clean.docx]

**Supplementary Materials**

**Supplementary Figures**


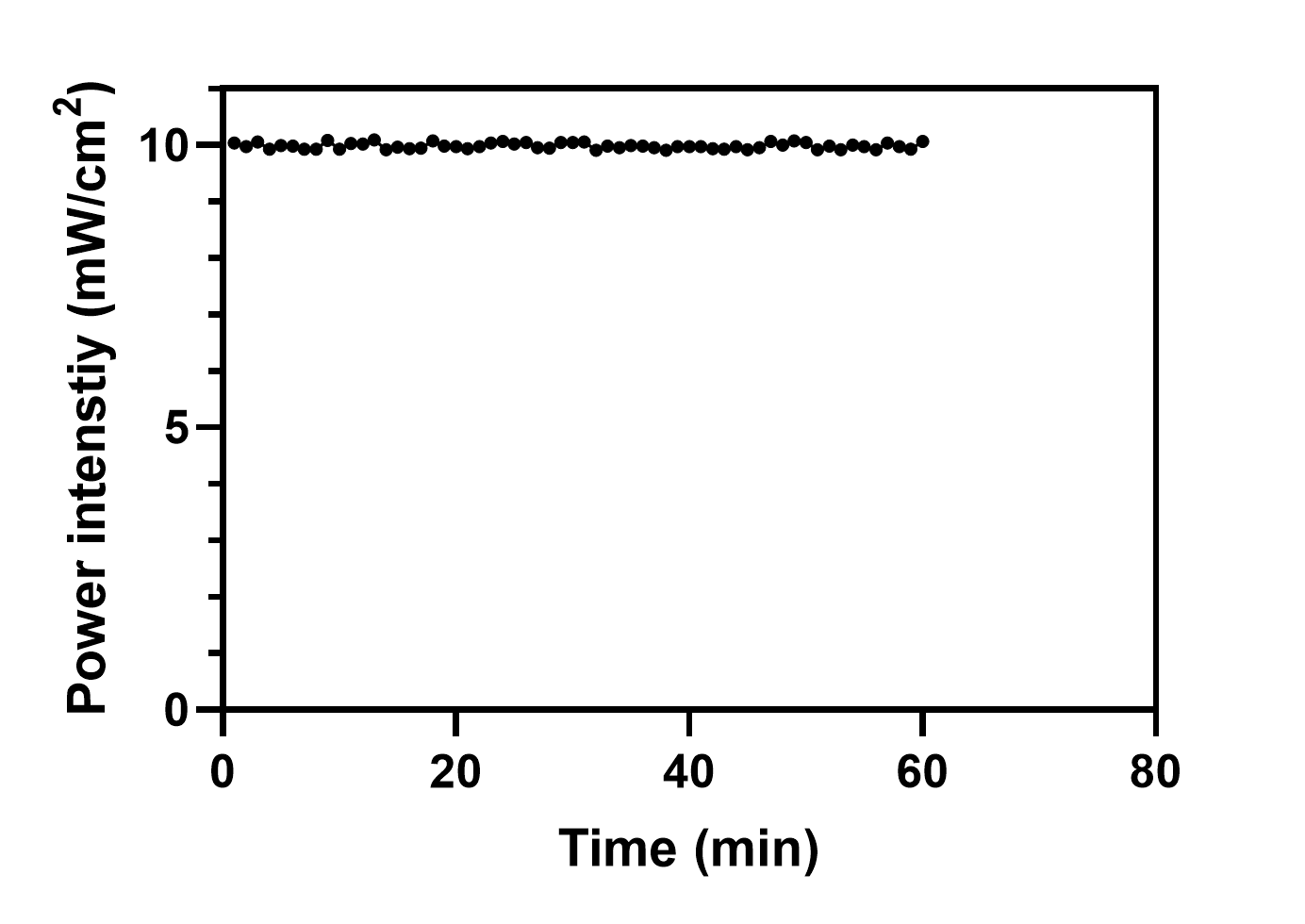


Fig. S1 Stability test of the output energy of the millimeter wave (MMW) irradiator during 60 minutes.


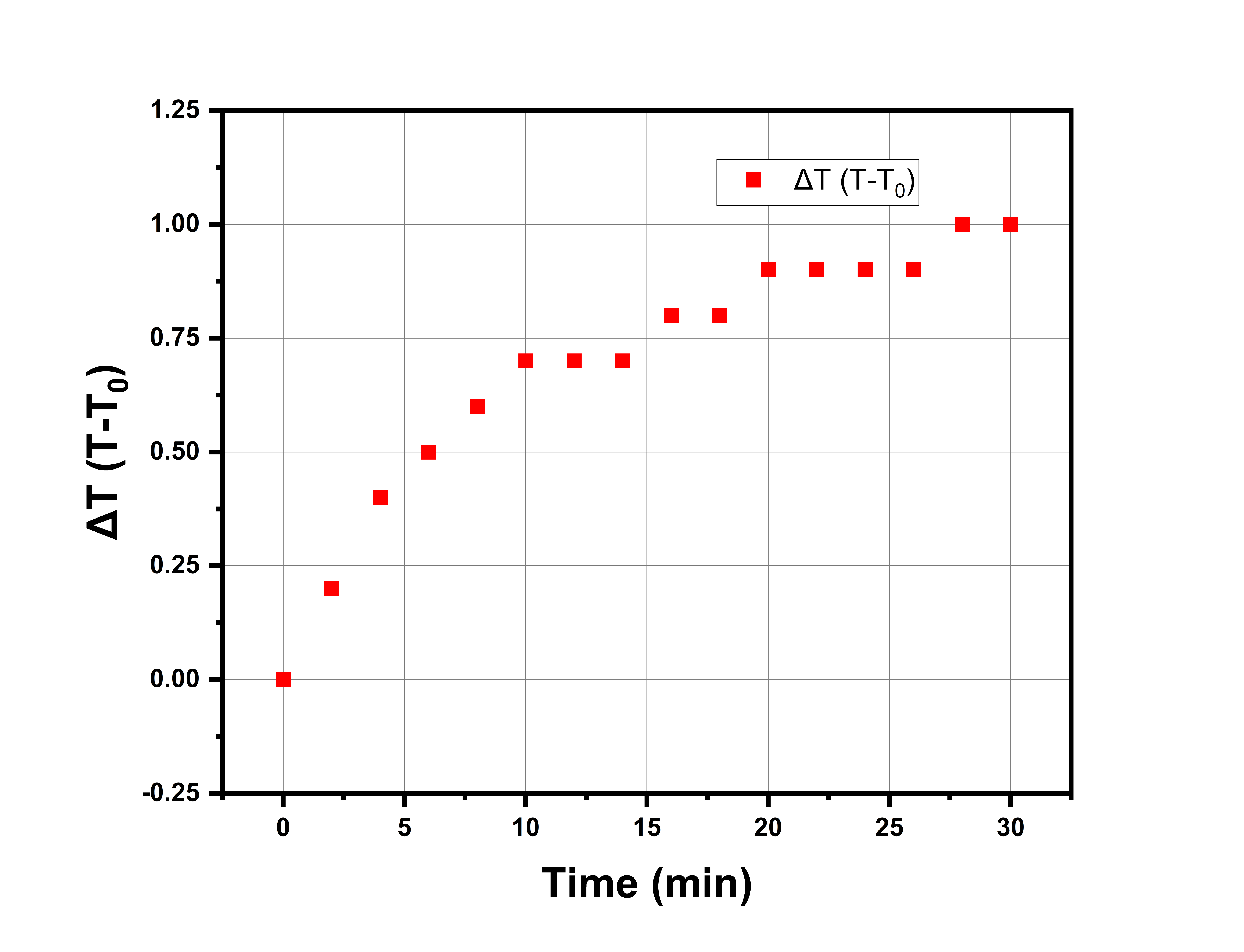


Fig. S2 Curve of temperature rise of PBS solution caused by MMW irradiation at room temperature of 25℃.


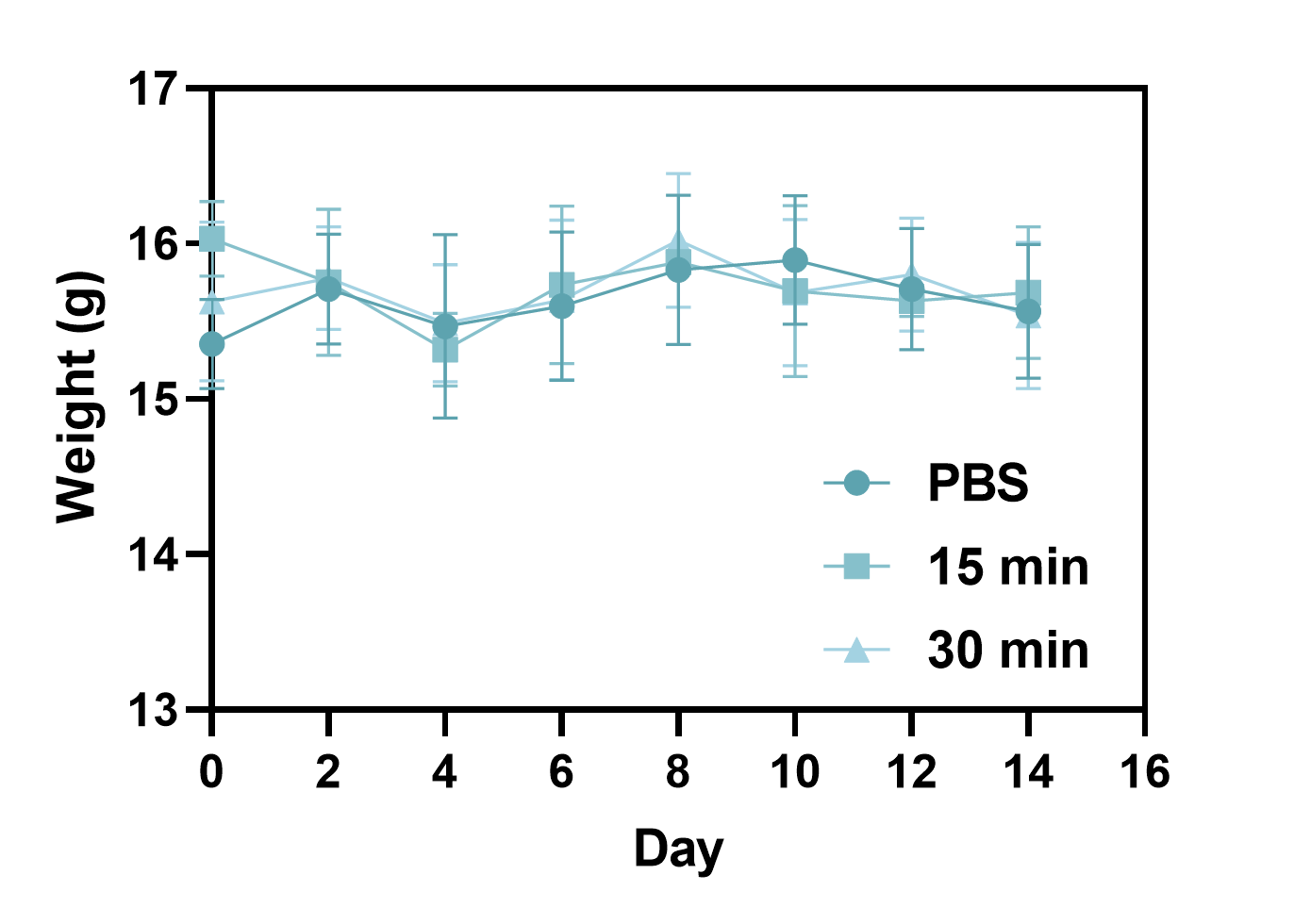


Fig. S3 Changes in body weight of different groups of mice during 15 days of MMW irradiation.


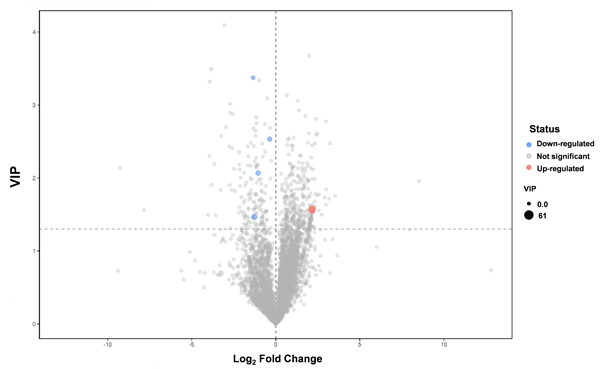


Fig. S4 Volcano plot of clustering analysis of anion metabolite profiling systems in the 30-minute MMW group versus the control group.


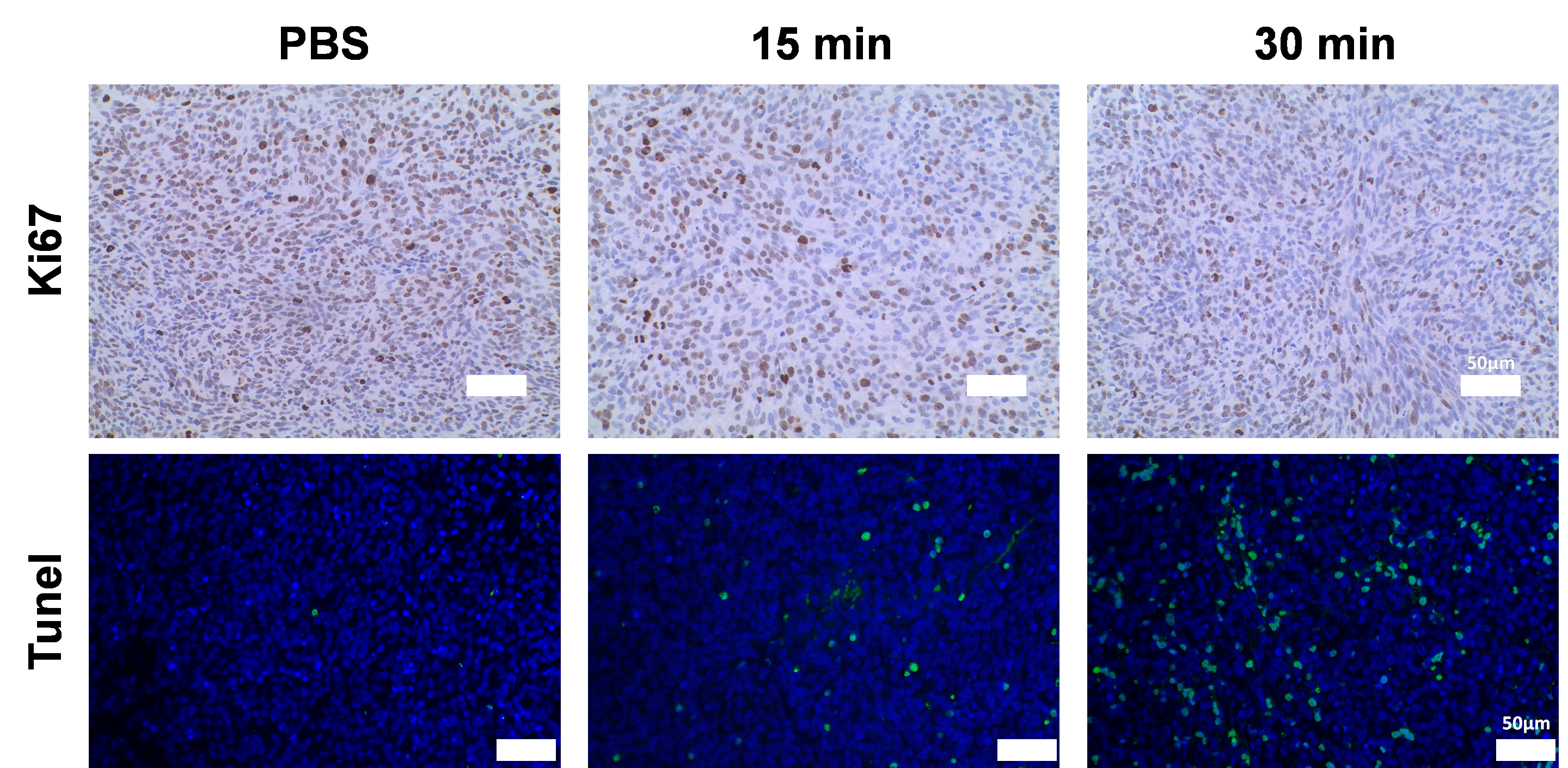


Fig. S5 Immunofluorescence staining results of Ki-67 and Terminal Deoxynucleotidyl Transferase mediated dUTP Nick-End Labeling (TUNEL) in 4T1 tumor tissues of mice after 15 days of different treatments (Scale bar: 50 μm).


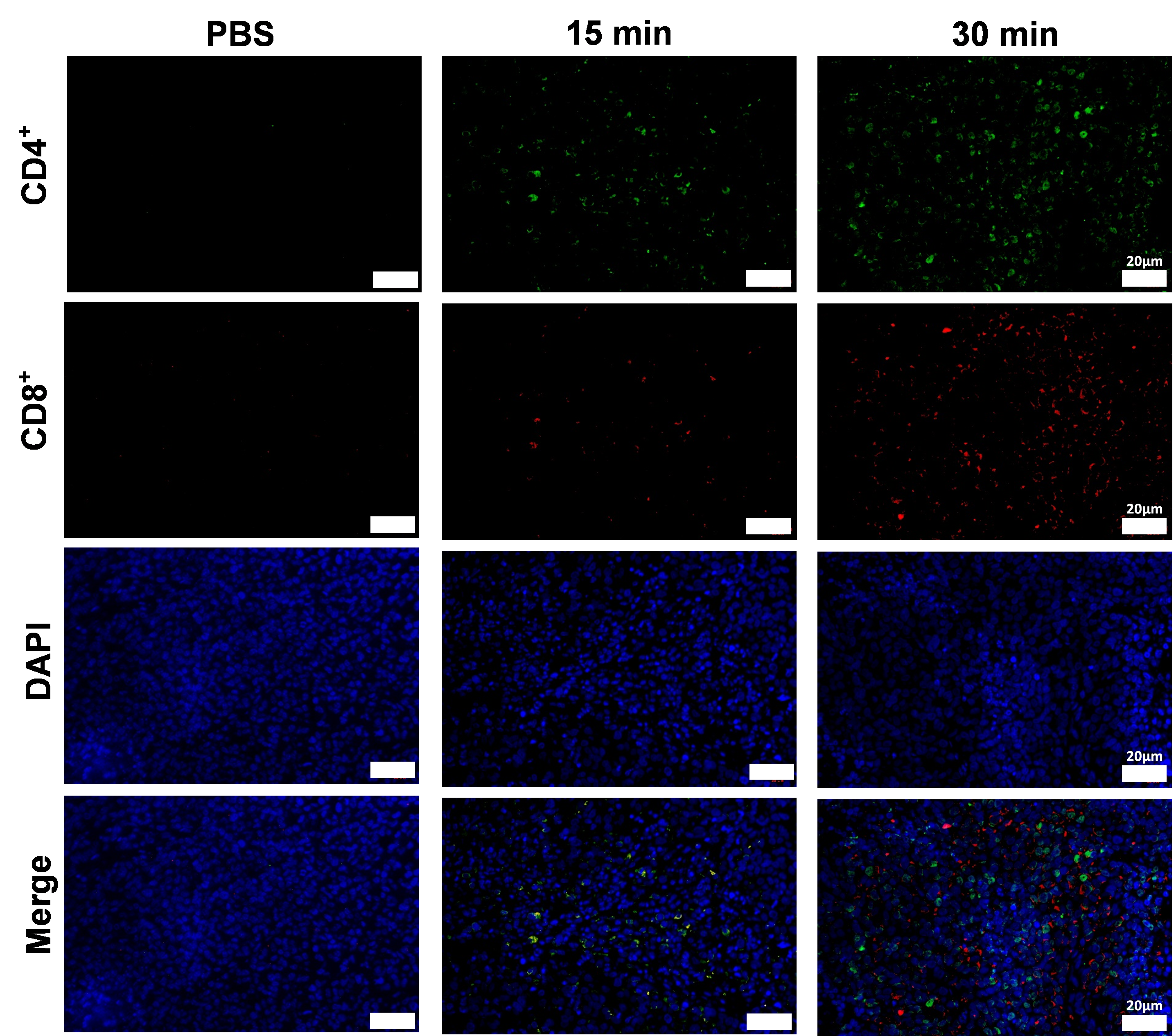


Fig. S6 Immunofluorescence staining results of CD4^+^, CD8^+^, DAPI and Merge in 4T1 tumor tissues of mice after 15 days of different treatments (Scale bar: 20 μm).


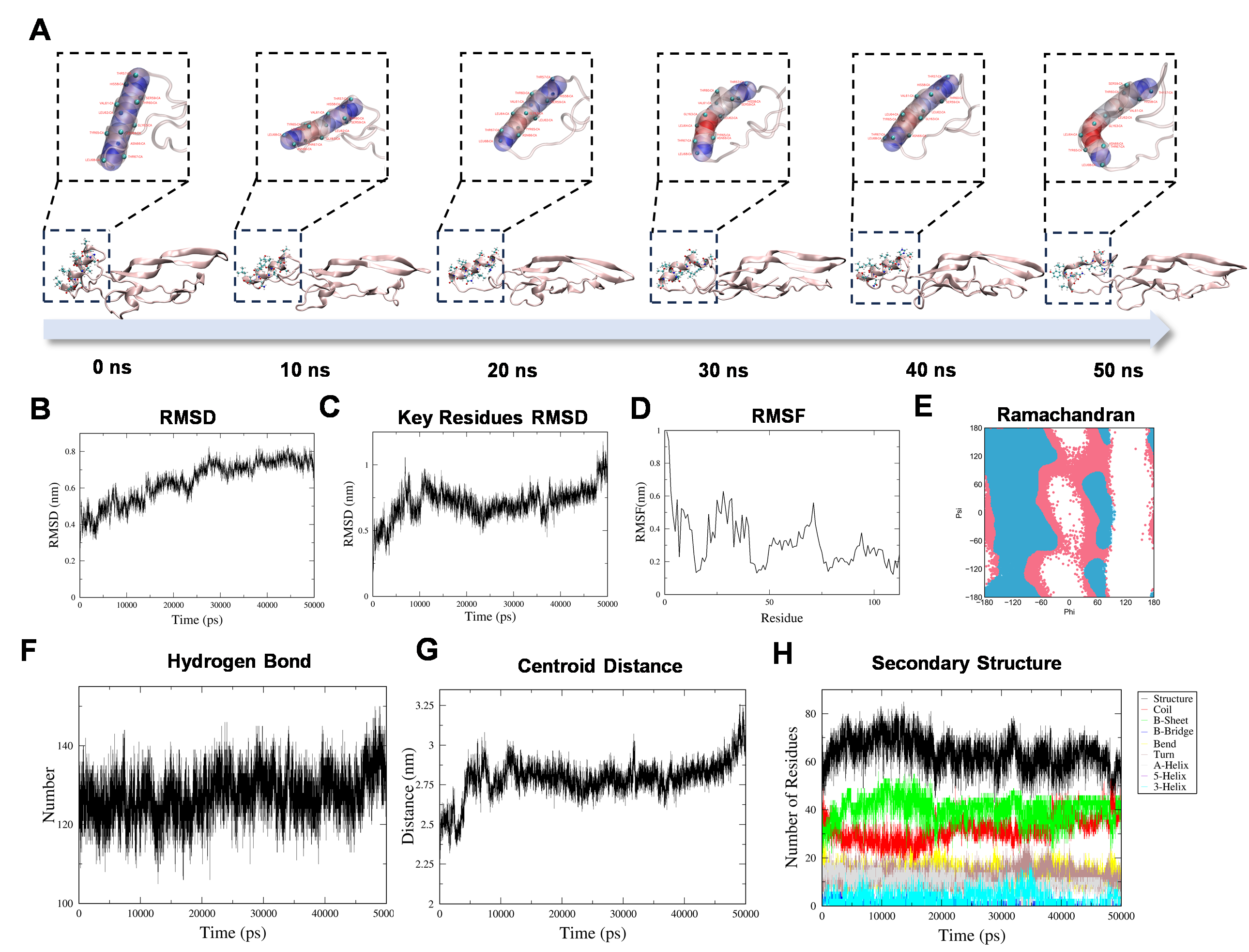


Fig. S7 Molecular dynamics was used to simulate the 50 ns MMW irradiation of TGF-β protein. (A) Molecular dynamics trajectory of TGF-β monomer in 50 ns simulation time. (B) TGF-β monomer RMSD within 50 ns. (C) RMSD of key residues of TGF-β monomer. (D) RMSF values of all residues of TGF-β chain during the simulation. (E) Ramachandran diagram of TGF-β monomer. (F) Number of hydrogen bonds in TGF-β monomer changes with the simulation time. (G) Distance between the centroid of the key residue in TGF-β monomer and the centroid of protein changes with time. (H) Secondary structure diagram of TGF-β monomer.


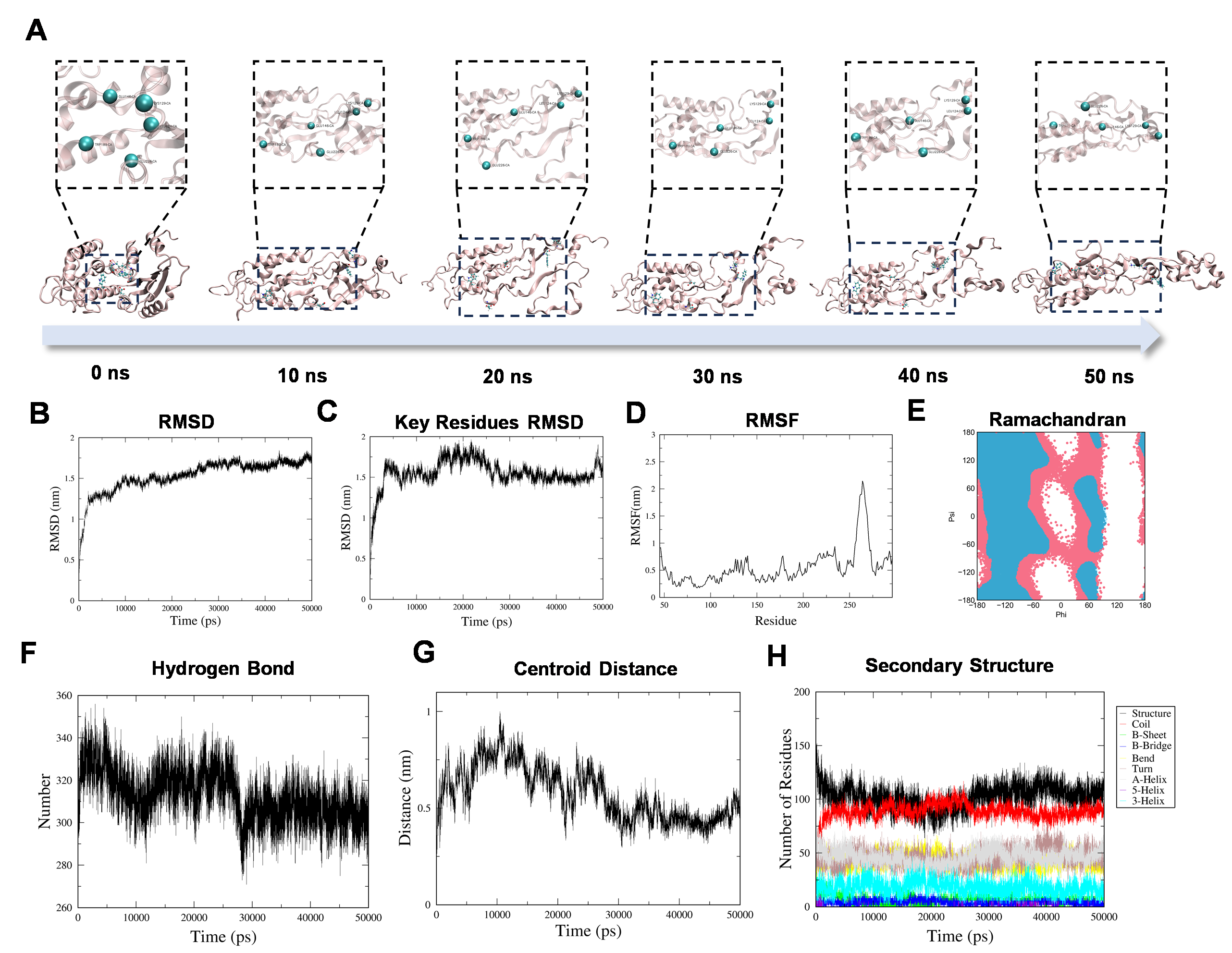


Fig. S8 Molecular dynamics was used to simulate the 50 ns MMW irradiation of CD38 protein. (A) Molecular dynamics trajectory of CD38 monomer in 50 ns simulation time. (B) CD38 monomer RMSD within 50 ns. (C) RMSD of key residues of CD38 monomer. (D) RMSF values of all residues of CD38 chain during the simulation. (E) Ramachandran diagram of CD38 monomer. (F) Number of hydrogen bonds in CD38 monomer changes with the simulation time. (G) Distance between the centroid of the key residue in CD38 monomer and the centroid of protein changes with time. (H) Secondary structure diagram of CD38 monomer.


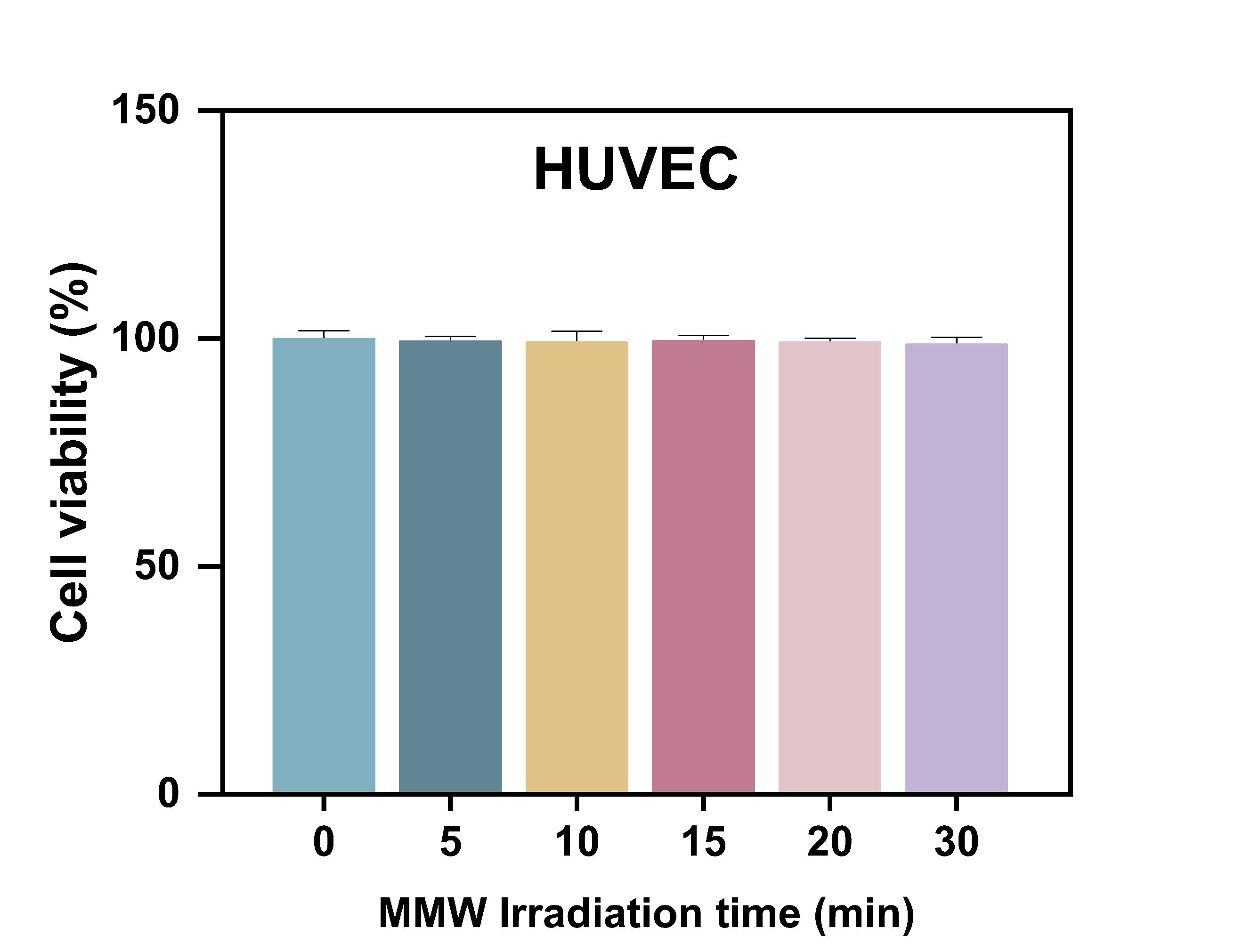


Fig. S9 Cell viability of HUVEC cells irradiated with MMW.


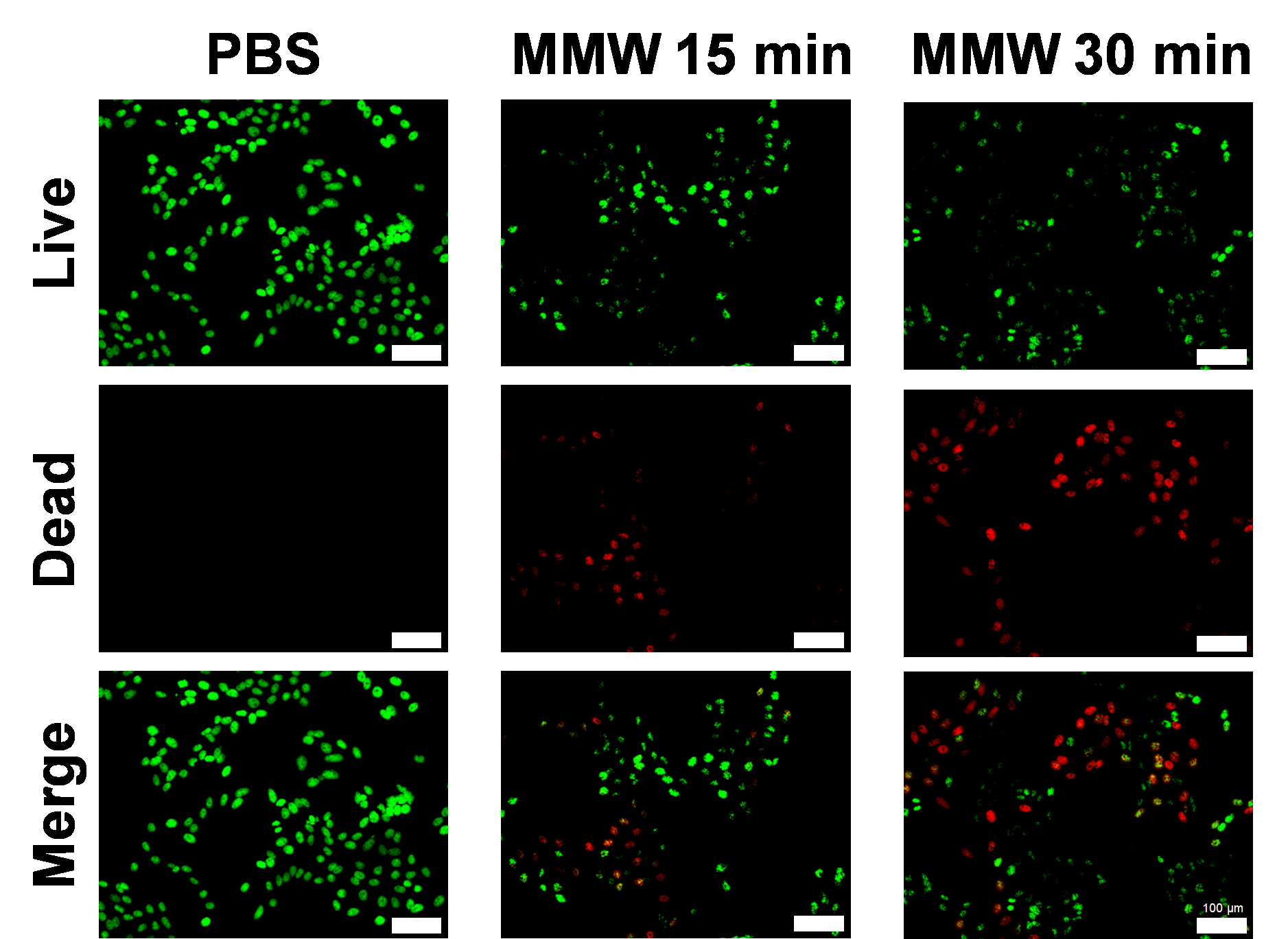


Fig. S10 Confocal microscopic observation of apoptosis in 4T1 cells after PBS, MMW 15 min, and MMW 30 min treatments (Scale bar: 100 μm).


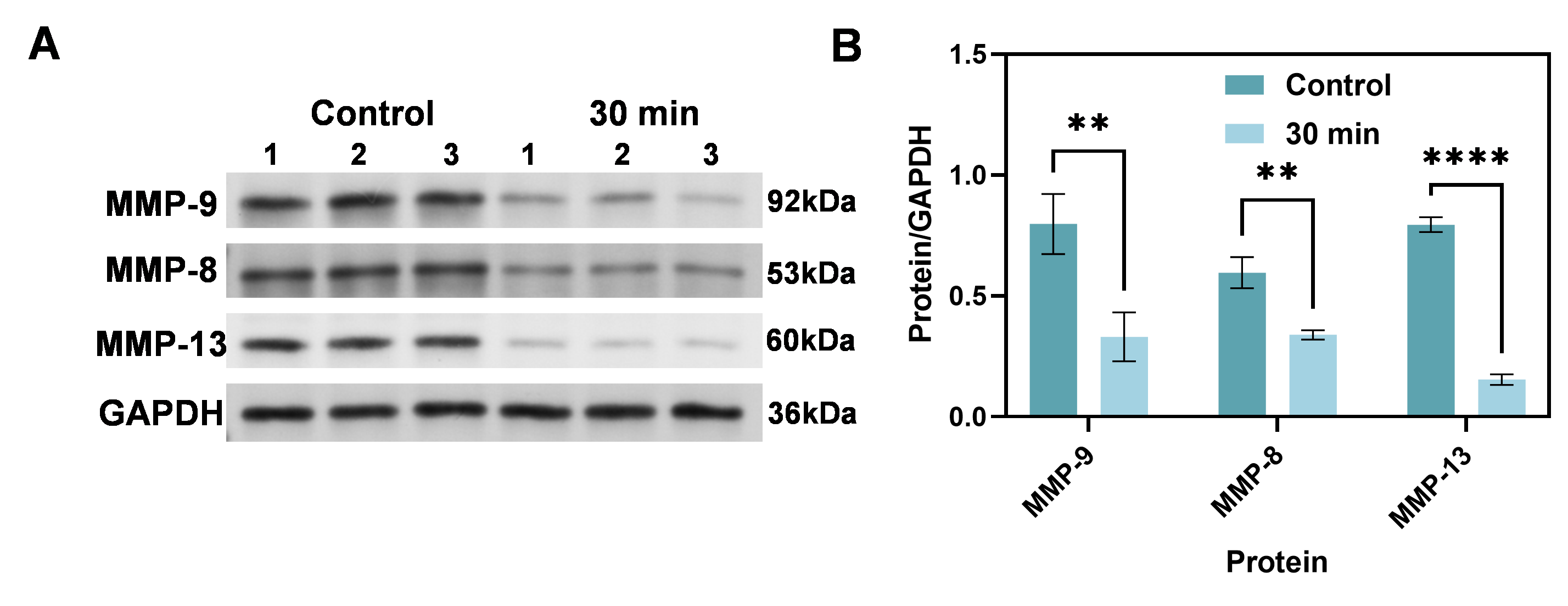


Fig. S11 (A) Effect of MMW 30 min versus control group treatment on matrix metalloproteinase MMP-9, MMP-8, and MMP-13 protein expression by Western Blot protein blotting. (B) Data of (A). Statistically significance was set ***p* < 0.01 and *****p* < 0.0001.


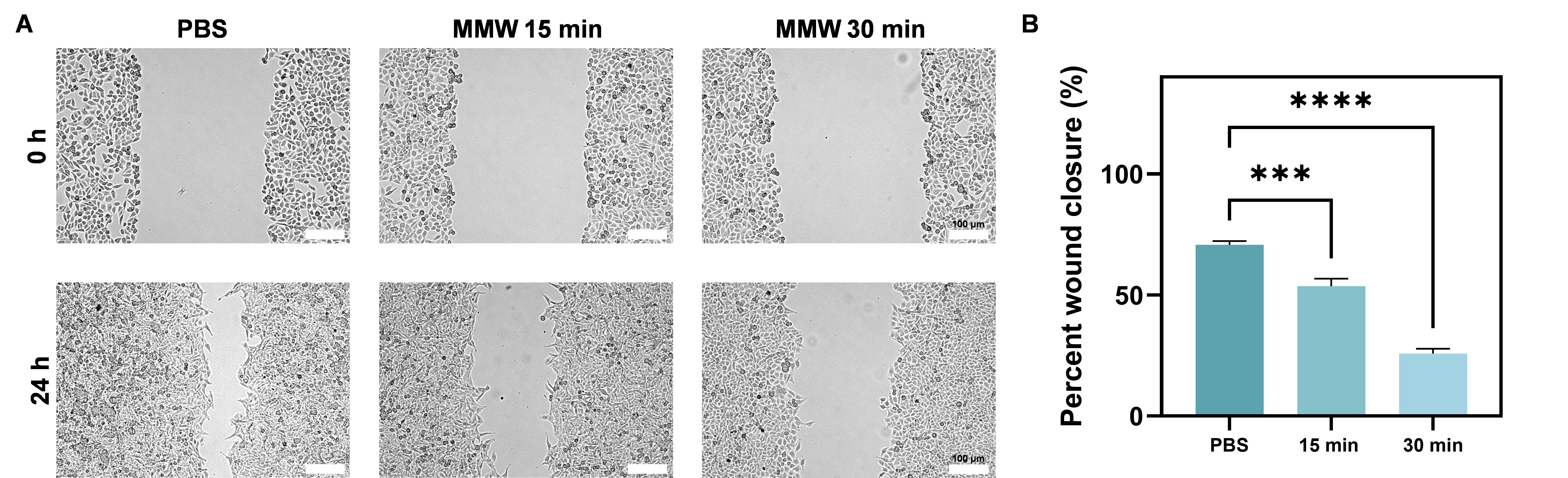


Fig. S12 (A) Using confocal microscope to observe the experimental results of scratch experimental results of 4T1 tumor cells under different treatment conditions (Scale bar:100μm). (B) Statistical analysis results of the percentage of scratch healing under different treatment conditions in (A). Statistically significance was set ****p* < 0.001 and *****p* < 0.0001.


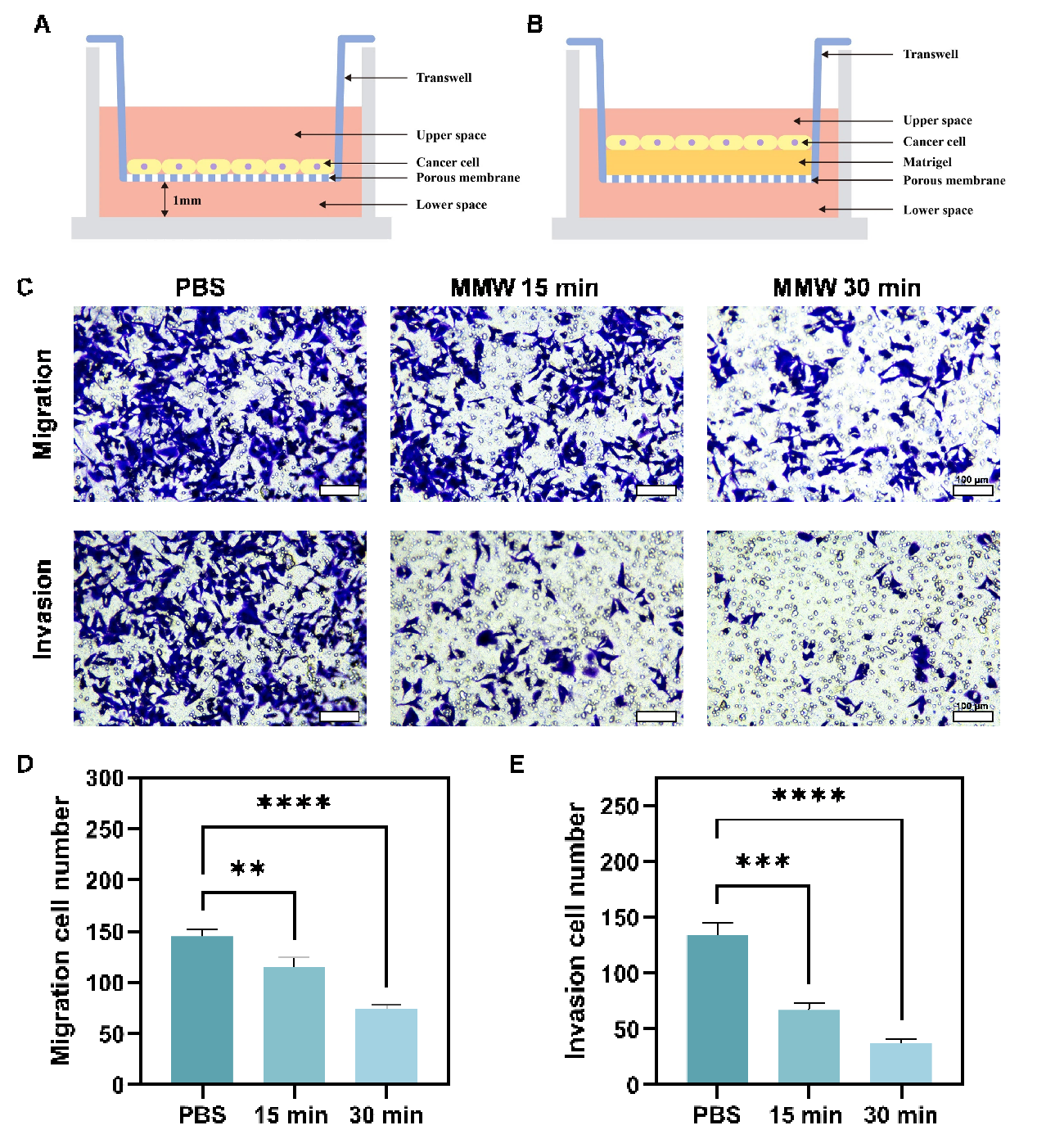


Fig. S13 (A) Installation design of the invasion experiment. The upper part is Transwell membrane, and the lower part is different areas, including upper space, cancer cell layer, porous membrane and lower space. 4T1 tumor cells need to pass through these areas to reach the lower space, which reflects the invasive ability of cells. (B) Device design of transfer experiment. The upper part is still Transwell membrane, and the lower part includes upper space, cancer cell layer, matrix, etc. 4T1 cells need to pass through these areas step by step to reach the lower space, which reflects the migration and metastasis ability of cells. (C) The experimental results of cell migration of 4T1 tumor cells under different treatment conditions were observed by a confocal microscope (Scale bar:100μm). (D) is the statistical analysis result of cell migration quantity under different treatment conditions in (C). (E) is the statistical analysis result of the number of cell invasions under different treatment conditions in (C). Statistically significance was set ***p* < 0.01, ****p* < 0.001 and *****p* < 0.0001.


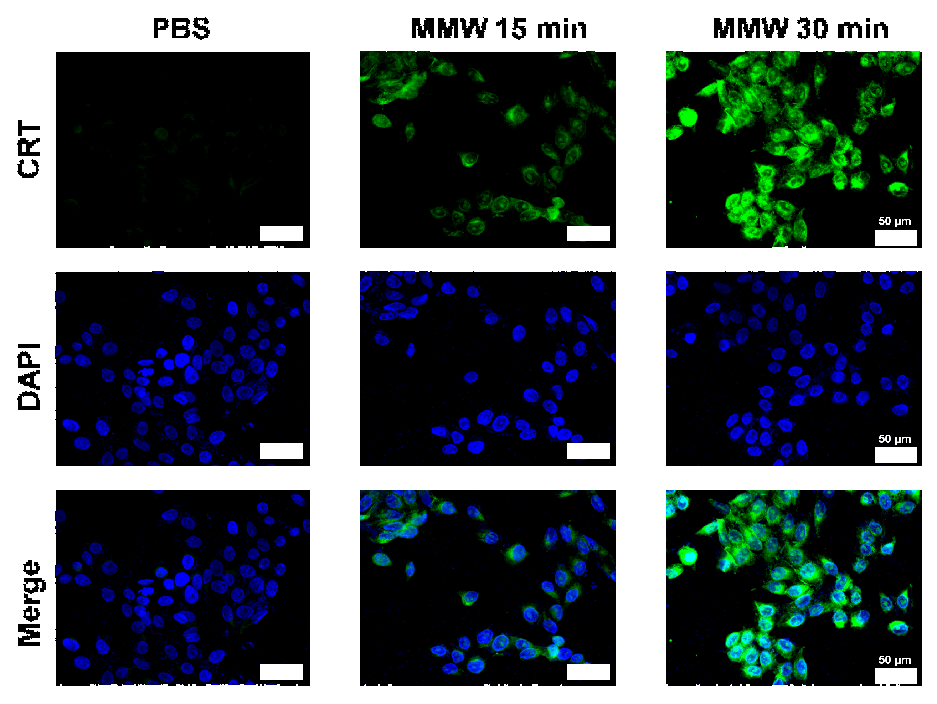


Fig. S14 The effects of PBS, MMW 15 min and MMW 30 min treatments on the expression of calreticulin (CRT) on the tumor surface were observed by confocal microscopy (Scale bar: 50 μm).


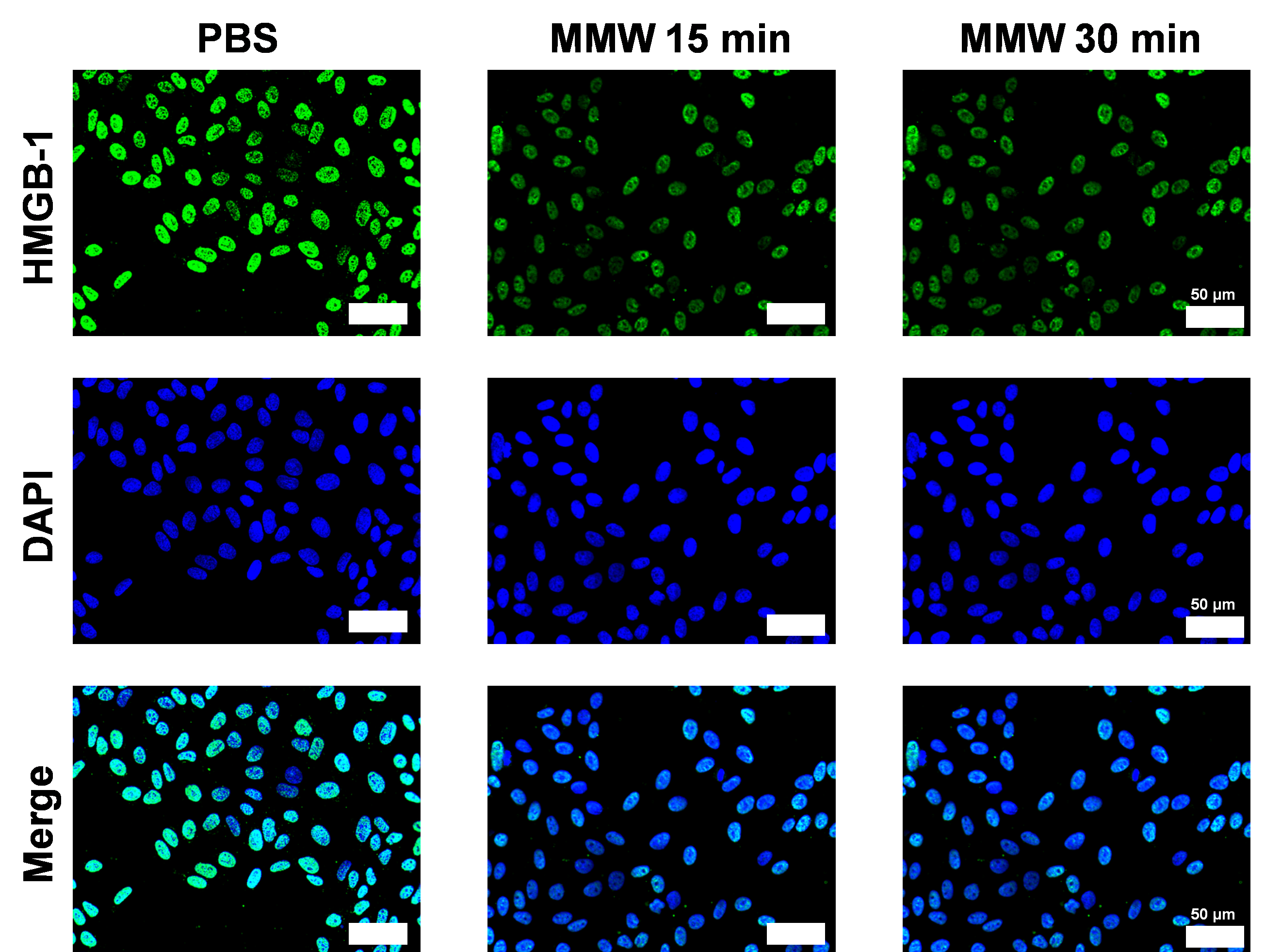


Fig. S15 The effects of PBS, MMW 15 min and MMW 30 min treatments on the changes of high mobility protein B1 (HMGB-1) levels within tumor nuclei were observed using confocal microscopy (Scale bar: 50 μm).


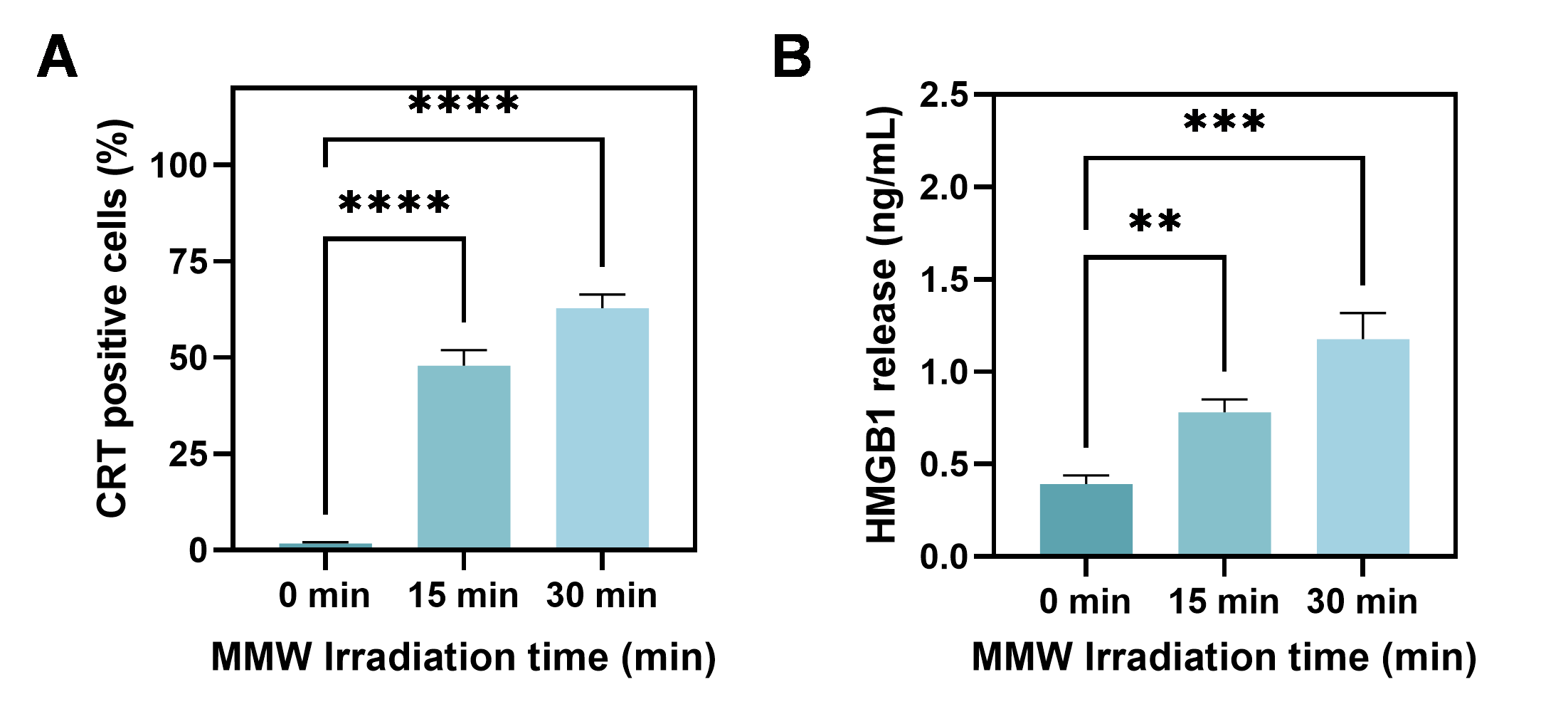


Fig. S16. (A) Percentage of CRT-positive cells determined by microscopic analysis. (B) Levels of HMGB-1 release measured by ELISA in cell culture supernatants. Statistically significance was set ***p* < 0.01, ****p* < 0.001, and *****p* < 0.0001.


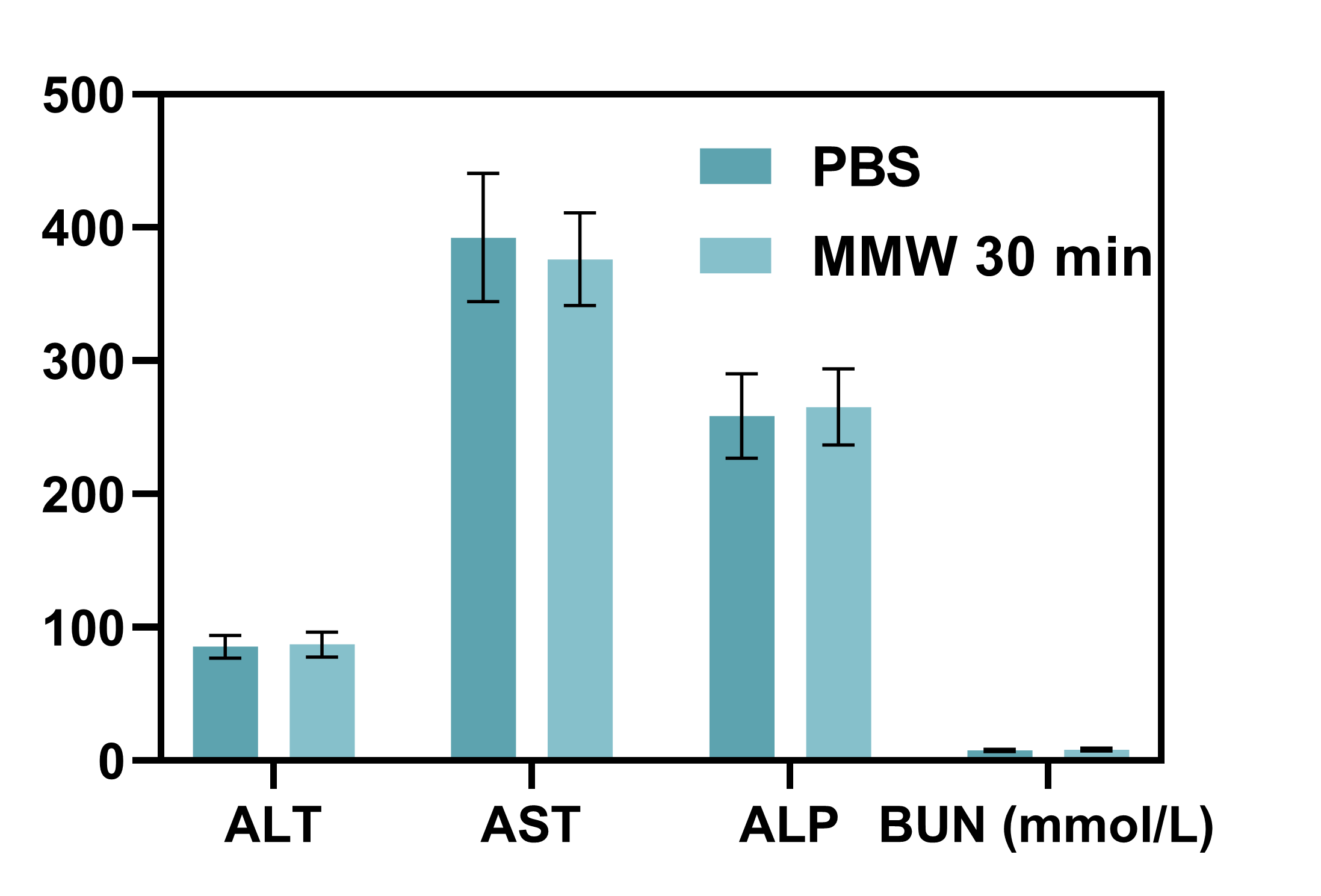


Fig. S17 Effect of MMW irradiation on biochemical indices of serum liver and kidney functions in mice. The horizontal coordinates represent the concentration levels (mmol/L) of alanine aminotransferase (ALT), aspartate aminotransferase (AST), alkaline phosphatase (ALP), and blood urea nitrogen (BUN), respectively, reflecting the status of liver and kidney functions.


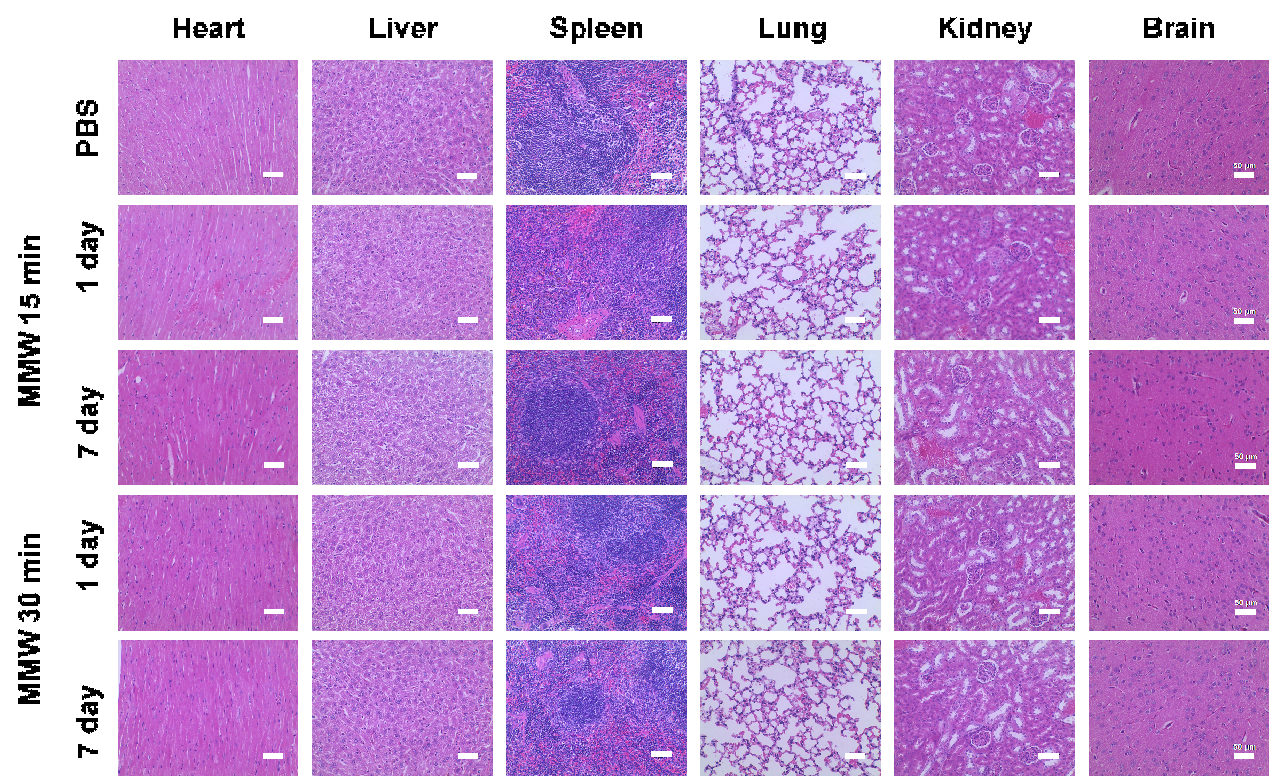


Fig. S18 H&E-staining results of various tissue sections of mice at 1 and 7 days after 15 and 30 min MMW irradiation (Scale bar: 50 μm).


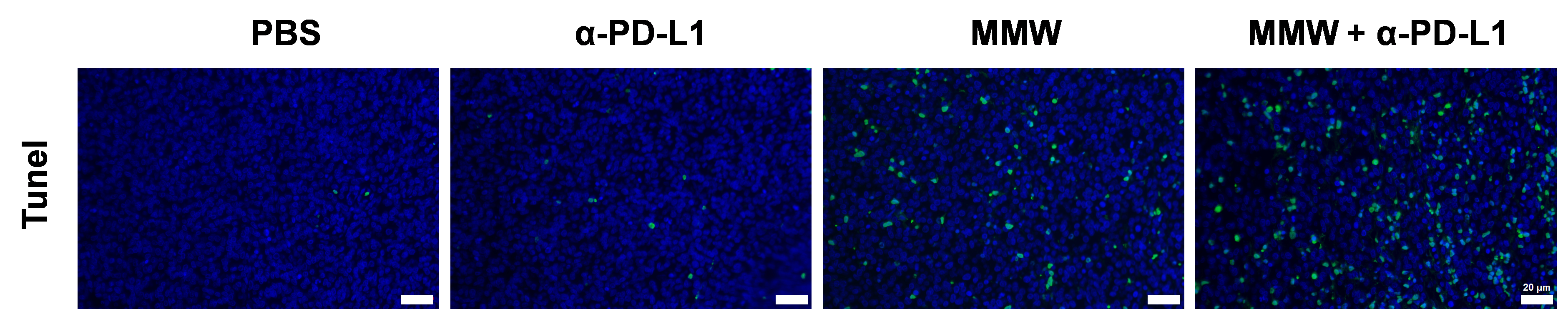


Fig. S19 Tunel-stained images of 4T1 unilateral tumors after treatment with PBS, α-PD-L1, MMW, and co-treatment with MMW and α-PD-L1, from left to right (Scale bar: 20 μm).


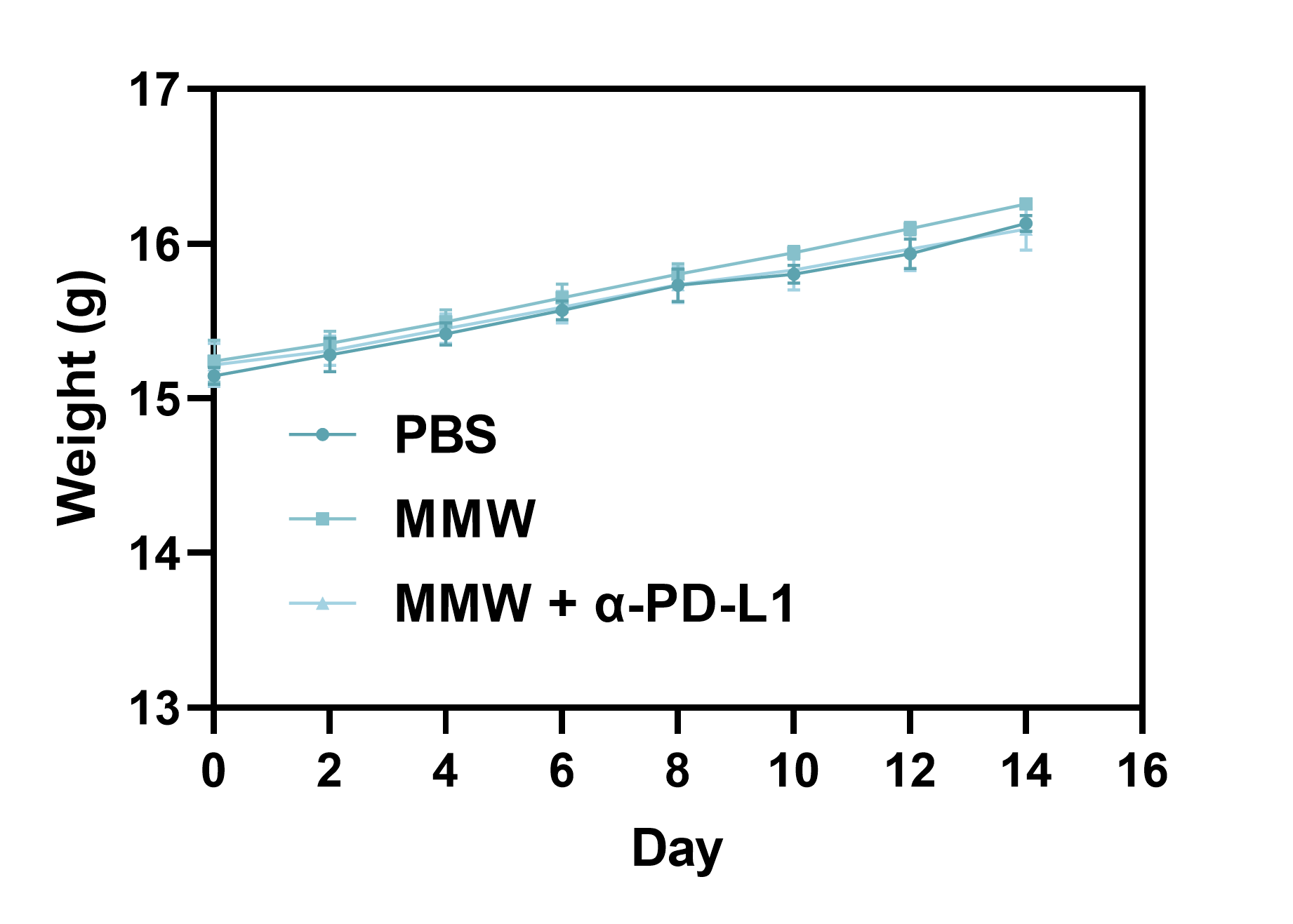


Fig. S20 Body weight changes of 4T1 unilateral tumors in mice after treatment with PBS, MMW, and MMW+α-PD-L1, respectively.


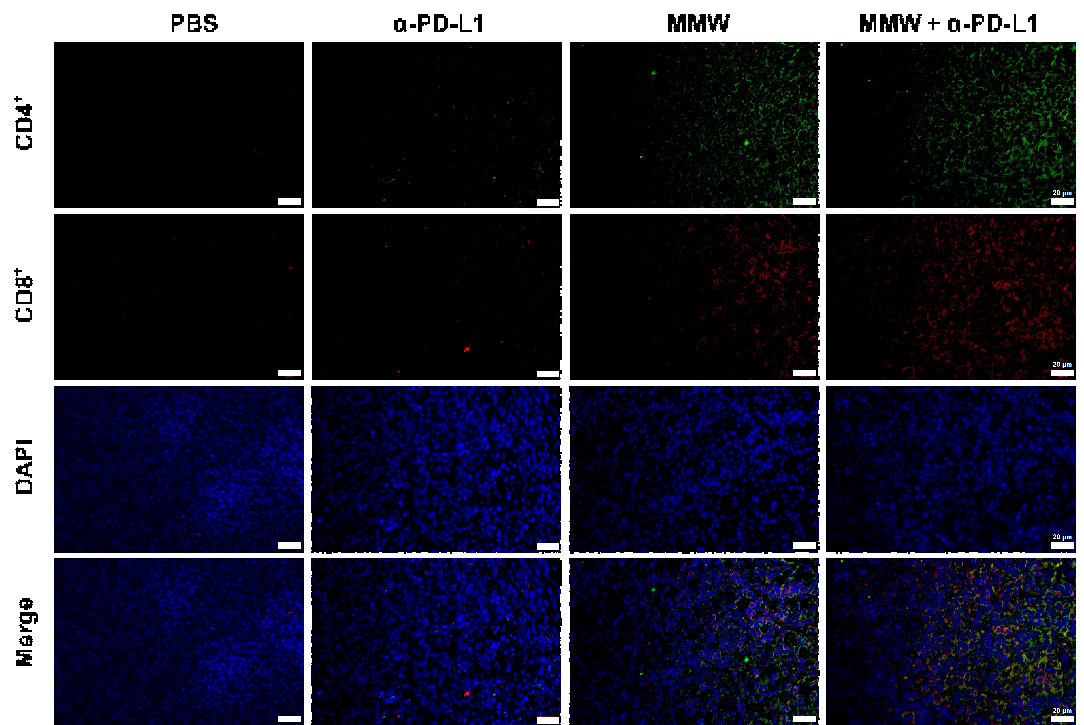


Fig. S21 Immunofluorescence staining results of CD4^+^, CD8^+^, DAPI and Merge in 4T1 tumor tissues of mice after 15 days of different treatments (Scale bar: 20 μm).


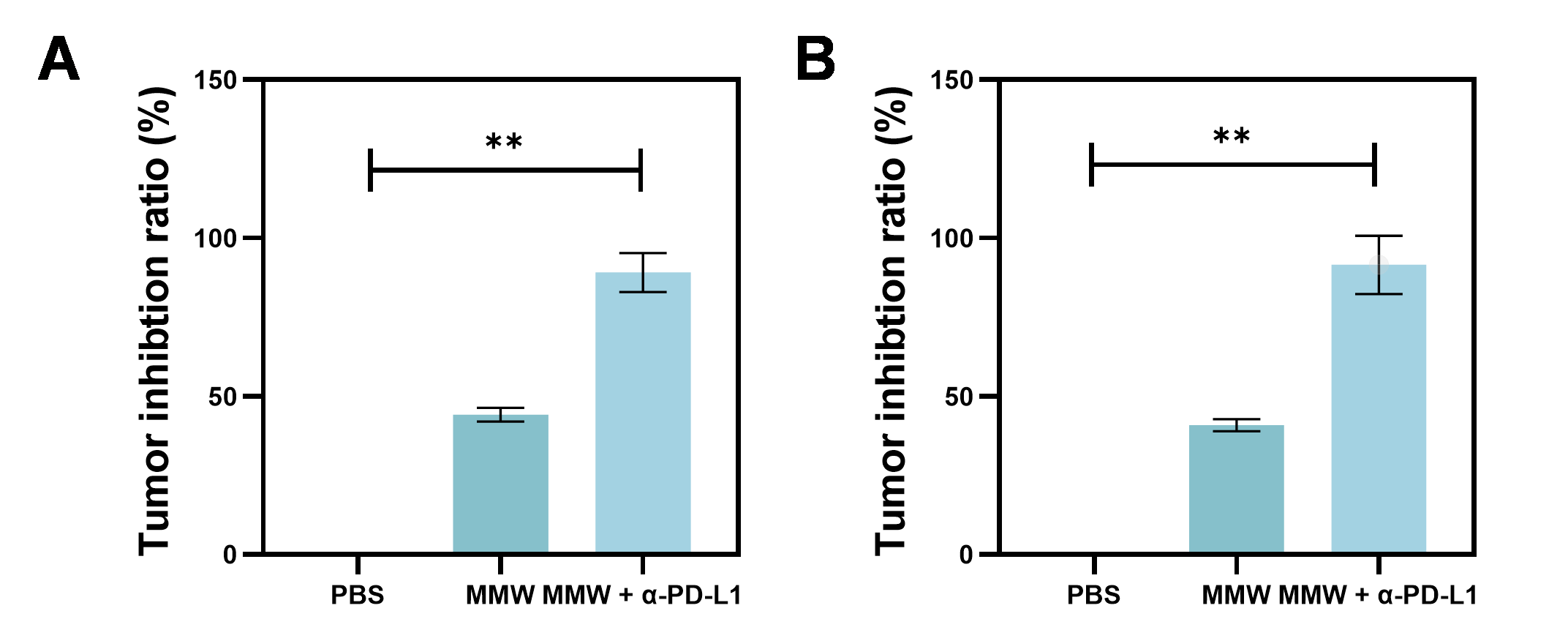


Fig. S22 (A) Primary 4T1 tumor, and (B) Distance 4T1 tumor suppression rates in mice (Primary 4T1 tumor treated with PBS, MMW, and MMW+α-PD-L1, respectively). Statistically significance was set ***p* < 0.01.


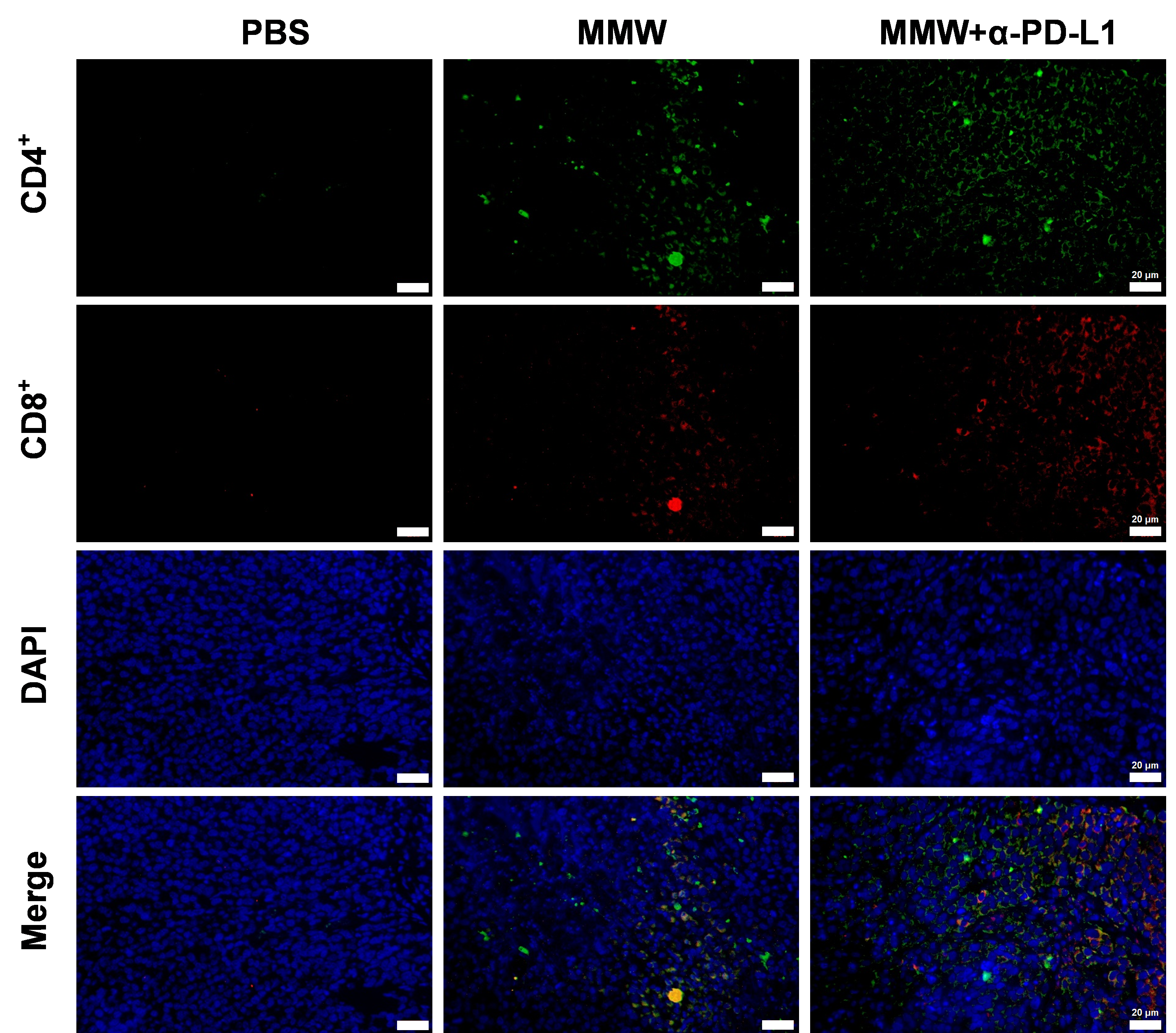


Fig. S23 Immunofluorescence staining results of CD4^+^, CD8^+^, DAPI and Merge in distant 4T1 tumor tissues of mice after 15 days of different treatments (Scale bar: 20 μm).


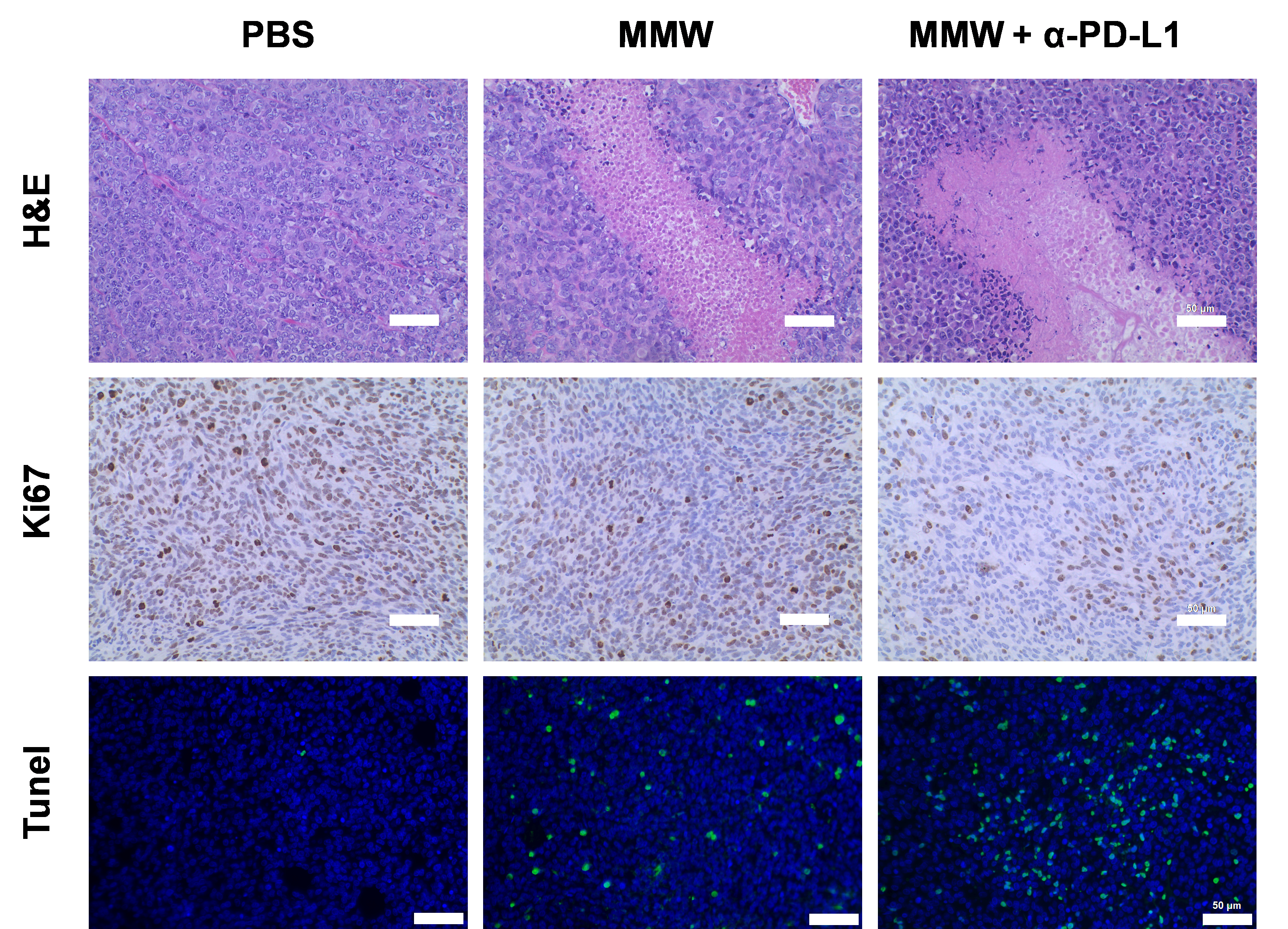


Fig. S24 H&E, Ki67, and Tunel staining results of distance tumors during primary tumor treatment (treated with PBS, MMW, and MMW+α-PD-L1, respectively) (Scale bar: 50 μm).


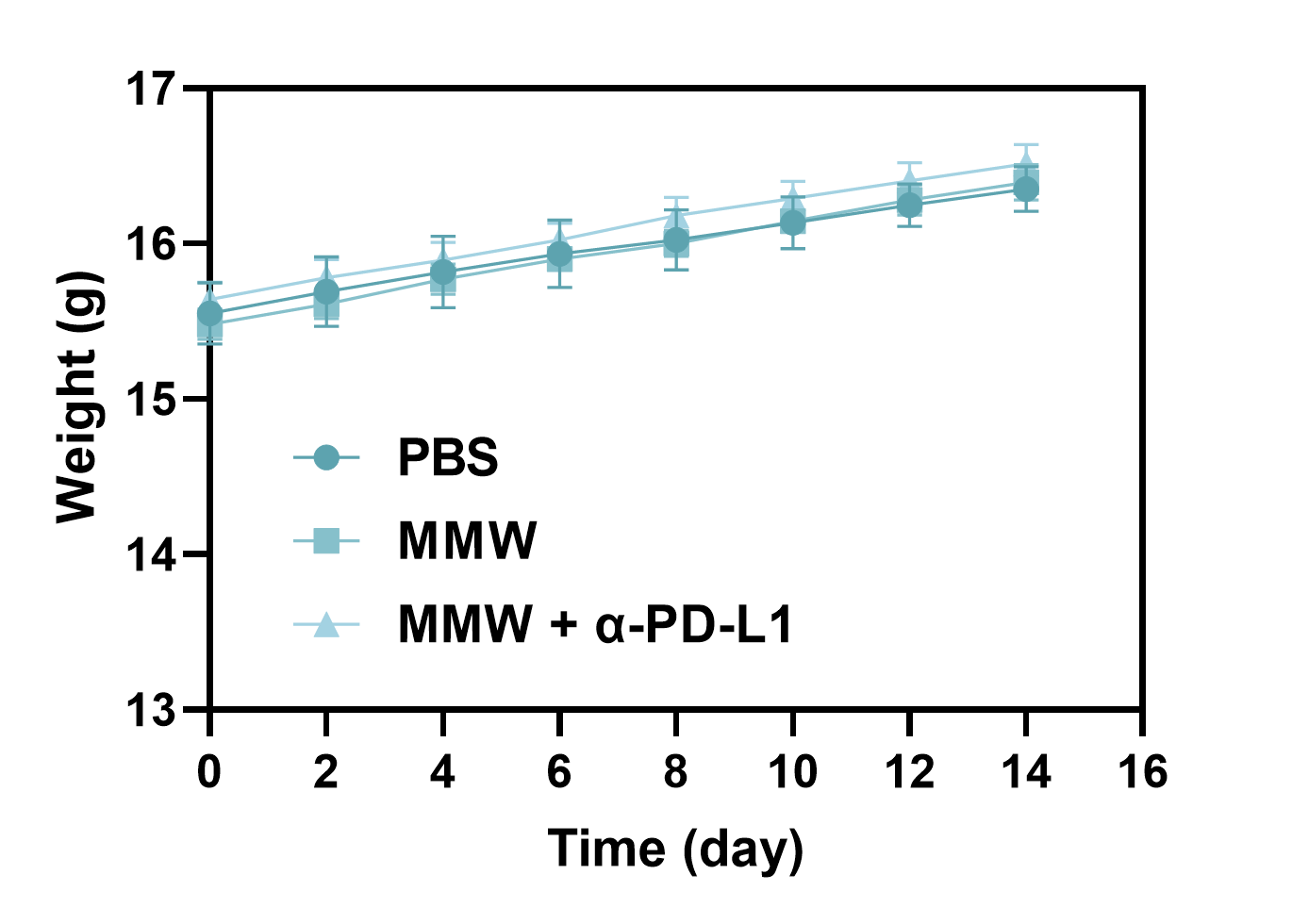


Fig. S25 Changes in body weight of mice during treatment of primary tumors in bilateral tumors (treated with PBS, MMW, and MMW+α-PD-L1, respectively).


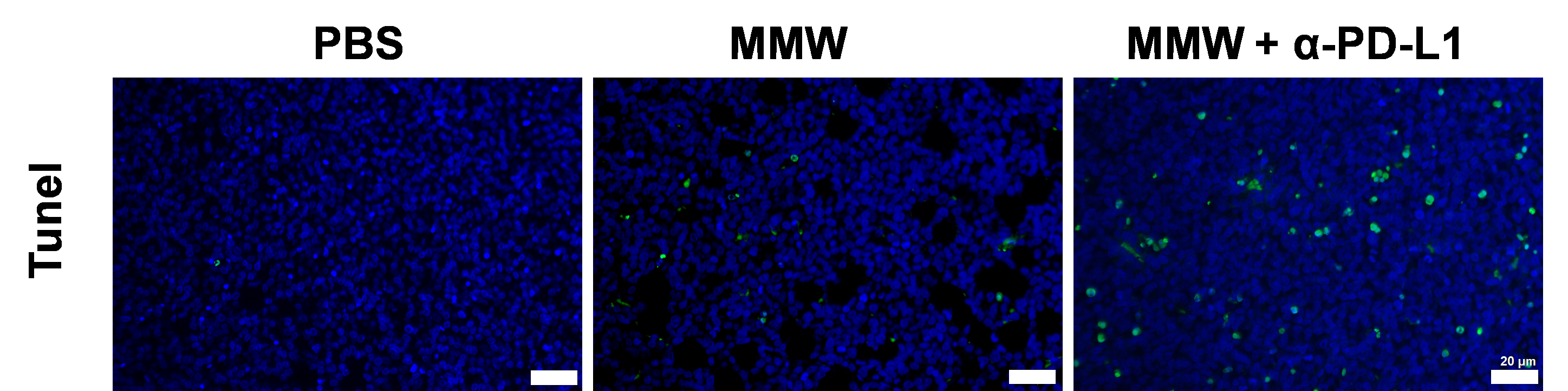


Fig. S26 Tunel-stained images of 4T1 anaplastic tumors treated with PBS, MMW, and MMW+α-PD-L1, respectively (Scale bar: 20 μm).


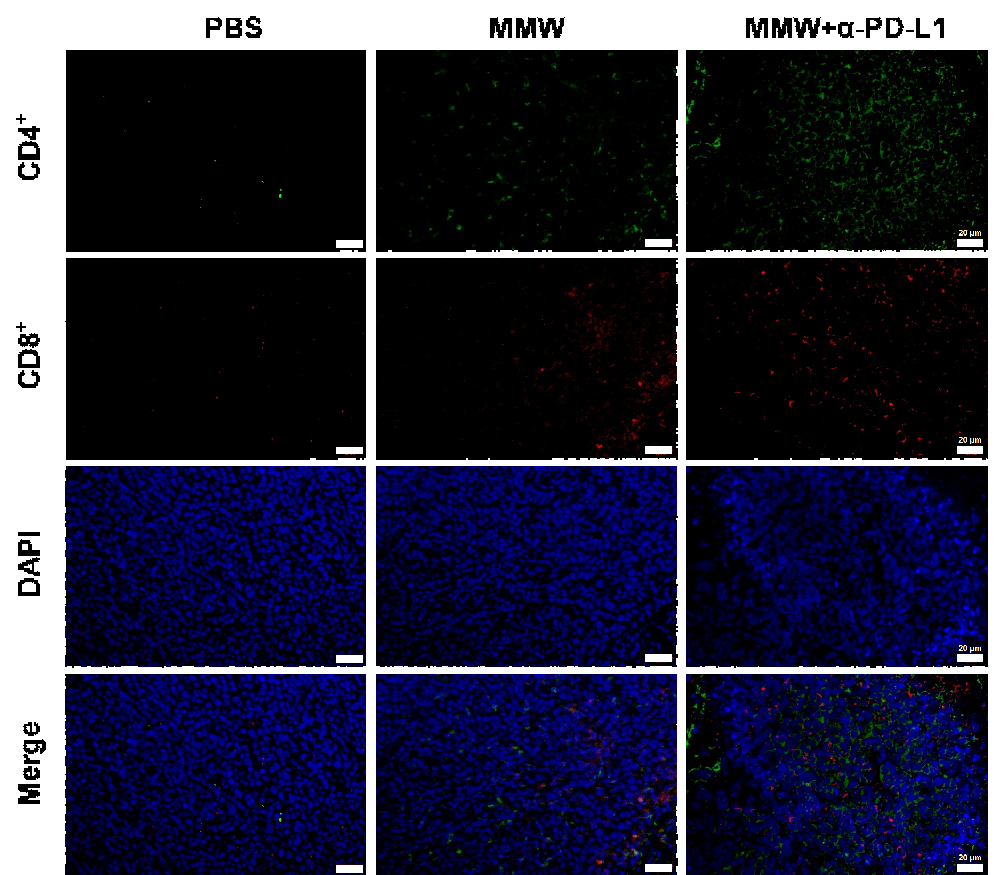


Fig. S27 Immunofluorescence staining results of CD4^+^, CD8^+^, DAPI and Merge in the tissues of 4T1 regenerative tumor of mice after 33 days of different treatments (Scale bar: 20 μm).


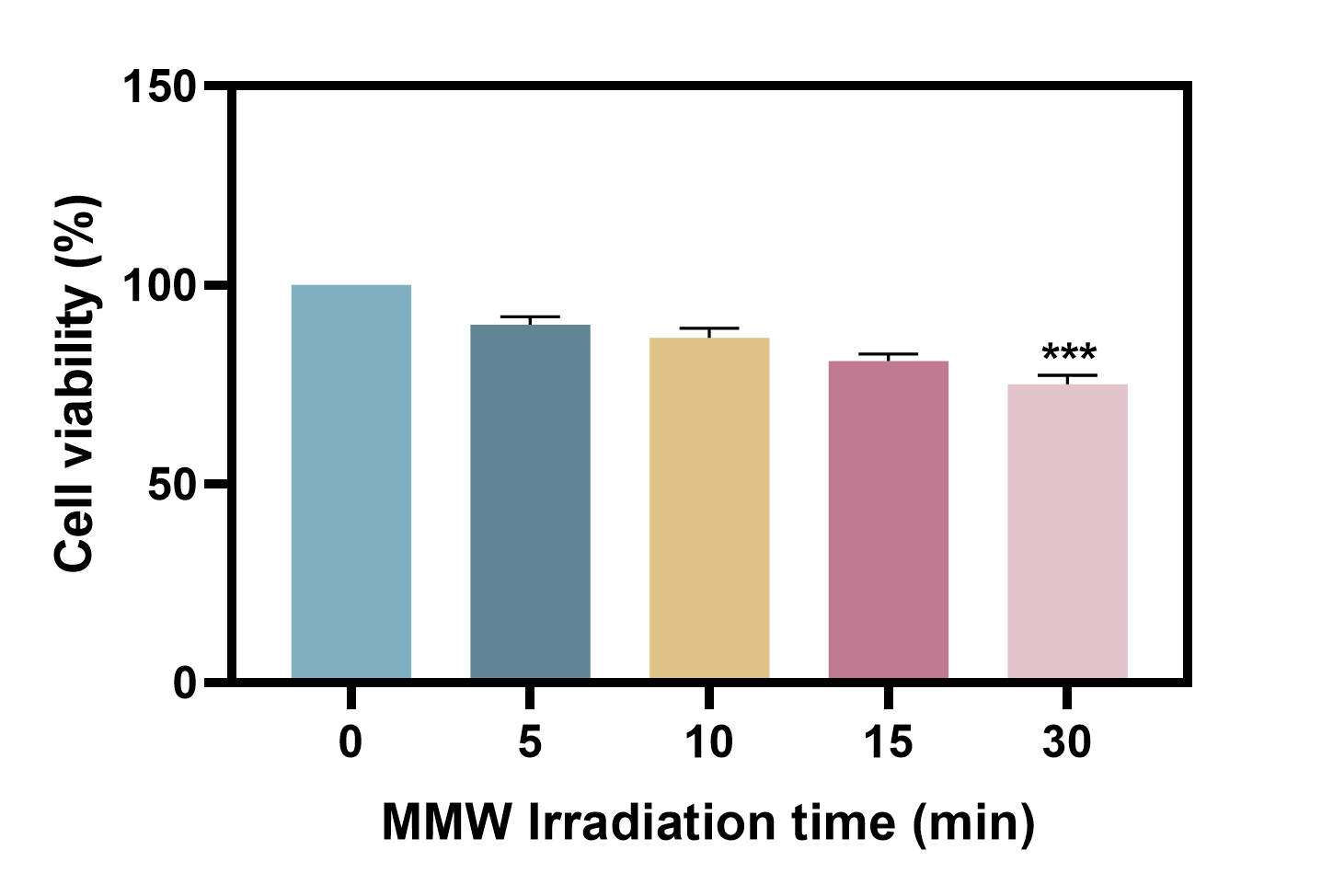


Fig. S28 Cell viability of CT26 cells after different times of MMW irradiation (0, 5, 10, 15, 30 min). ****p* < 0.001.


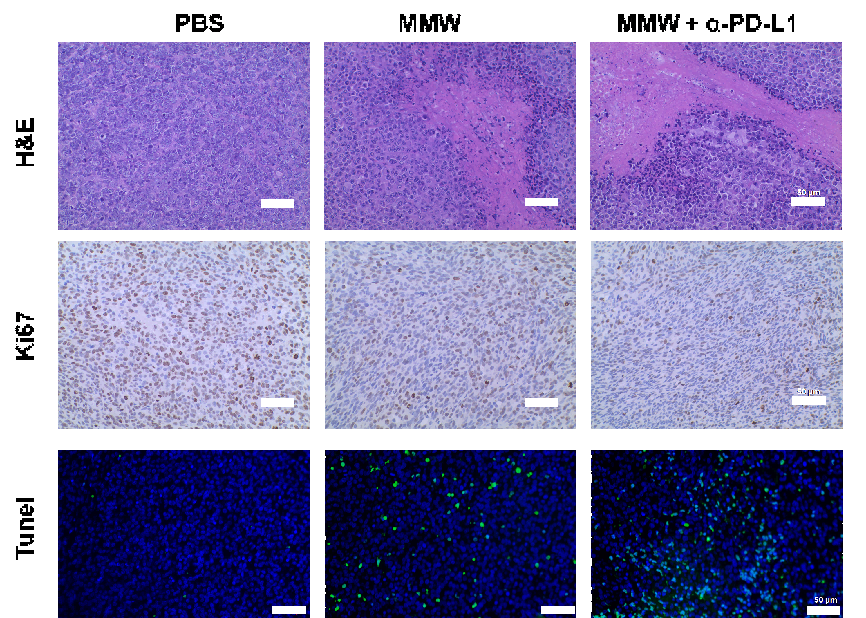


Fig. S29 H&E, Ki67, and Tunel staining results of CT26 tumors during treatment with PBS, MMW, and MMW+α-PD-L1, respectively (Scale bar: 50 μm).


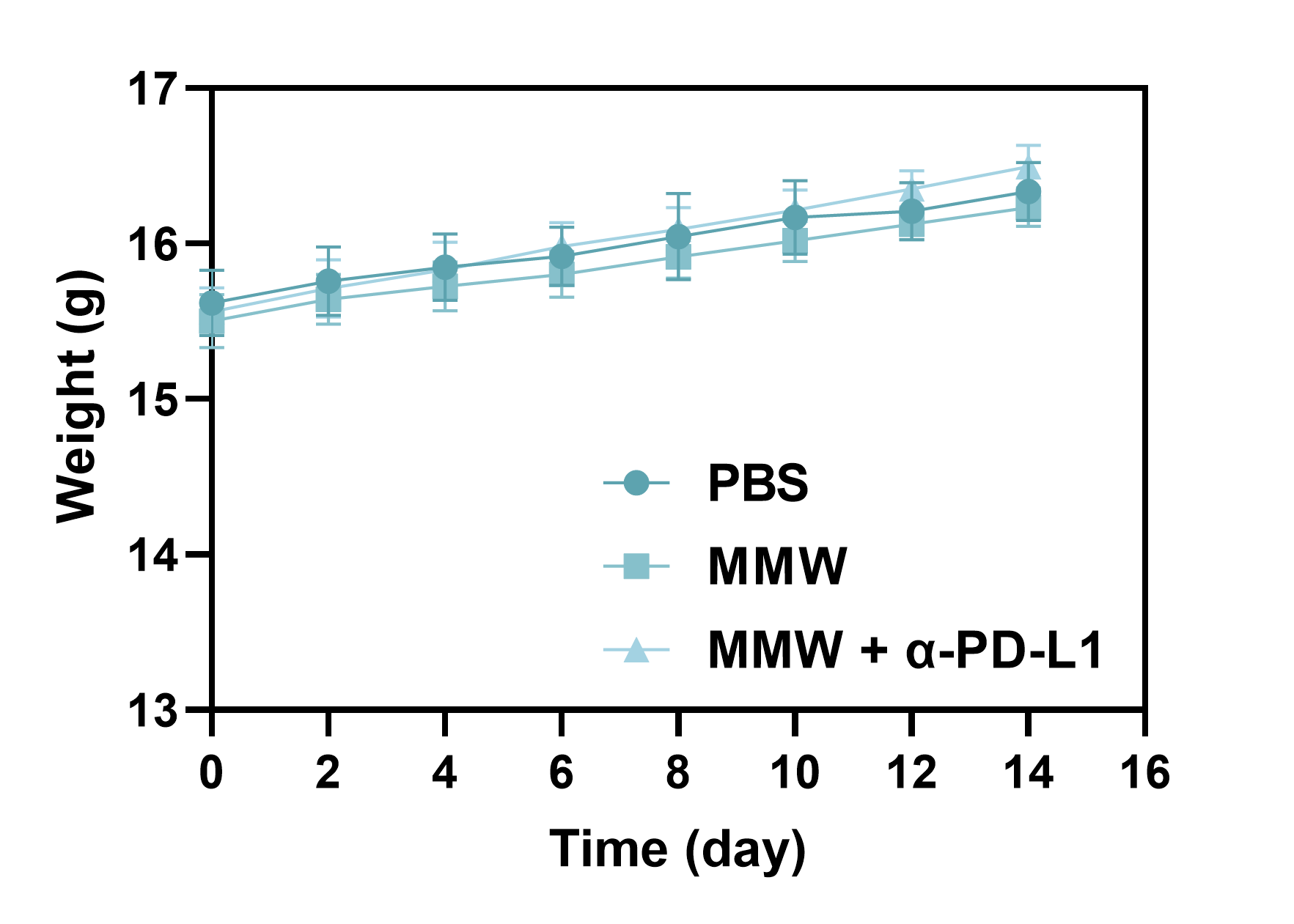


Fig. S30 Body weight changes in CT37 tumor-bearing mice after different treatments (PBS, MMW or MMW+α-PD-L1).


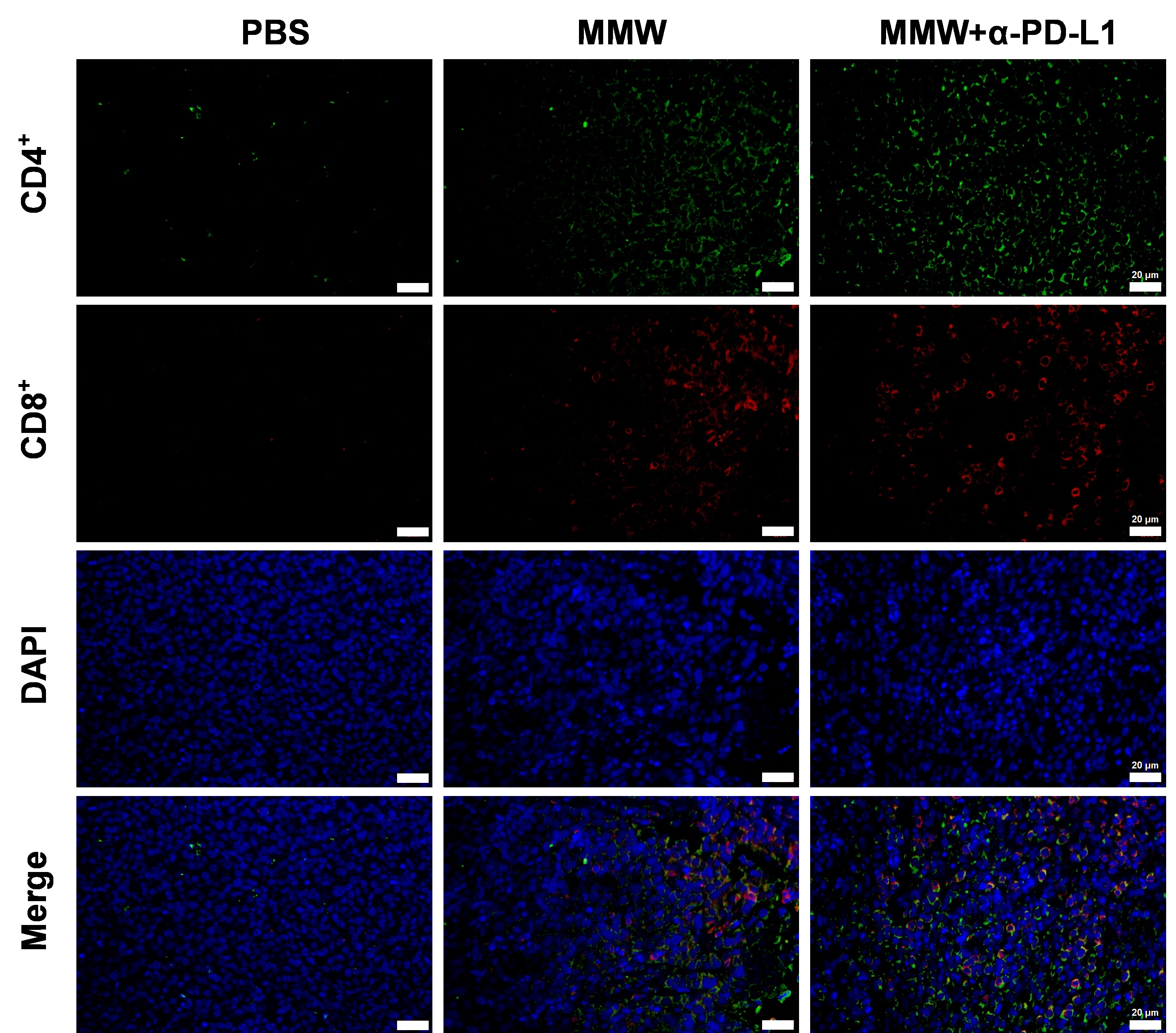


Fig. S31 Immunofluorescence staining results of CD4^+^, CD8^+^, DAPI and Merge in CT26 tumor tissues of mice after 15 days of different treatments (Scale bar: 20 μm).


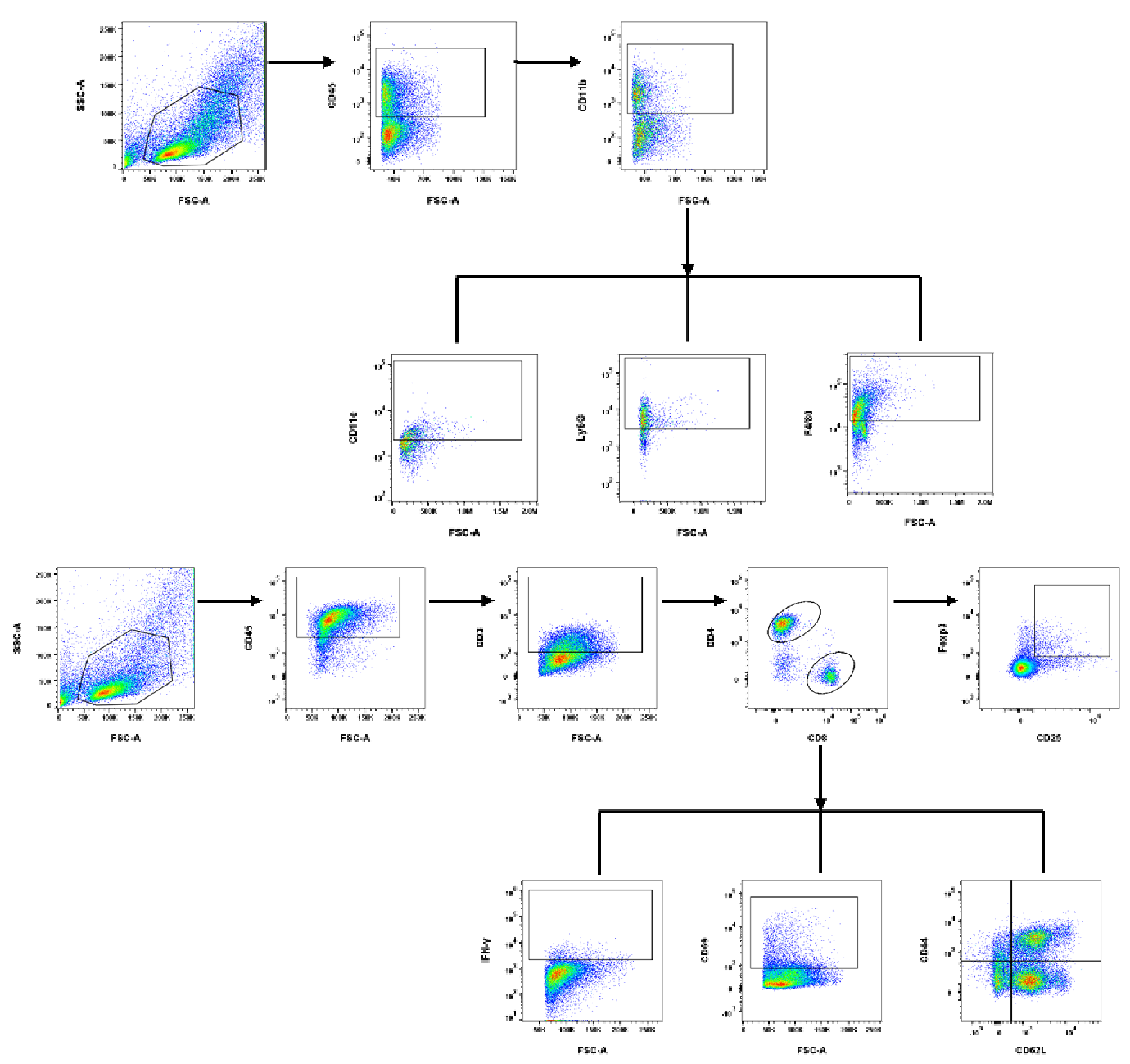


Fig. S32 Gating strategies for DCs (CD45^+^CD11b^+^CD11c^+^), neutrophiles (CD45^+^CD11b^+^Ly6G^+^), macrophages (CD45^+^CD11b^+^F4/80^+^), CD4^+^ T cells (CD45^+^CD3^+^CD4^+^), CD8^+^ T cells (CD45^+^CD3^+^CD8^+^), Treg (CD4^+^CD25^+^FoxP3^+^), activiated CD8^+^ T cells (CD8^+^IFN-β^+^ and CD8^+^CD69^+^), TCM (CD8^+^CD44^+^CD62L^+^), and TEM (CD8^+^CD44^+^CD62L^-^ ).


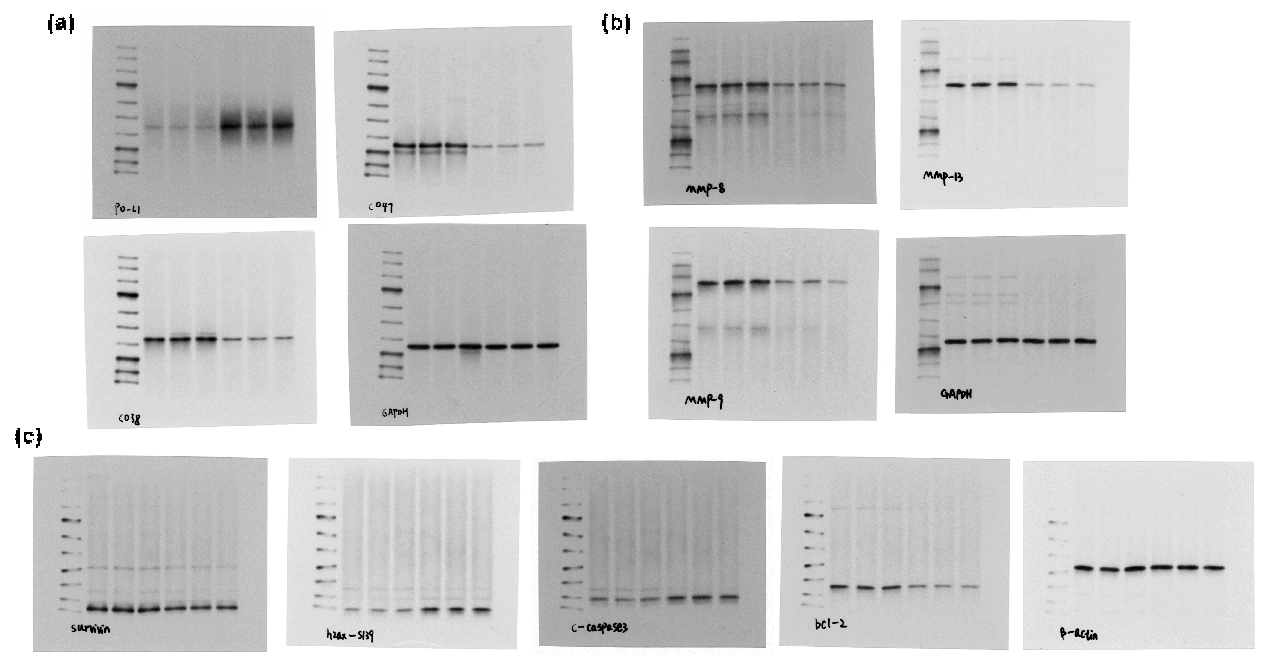


Fig. S33 (a) Western Blot protomembrane of Fig. 3F. (b) Western Blot protomembrane of Fig. S11. (c) Western Blot protomembrane of Fig. 5E.

**Materials and Methods**

**Millimeter wave (MMW) Exposure System and Thermal Monitoring**

The millimeter wave (MMW) device used in this study is a 35 GHz continuous wave solid-state source multifunctional therapeutic instrument (Beijing Oriental Conway Science and Technology Development Center, HT-601MMW), with a maximum output power density of 10 mW/cm^2^. Throughout all experiments, the irradiation parameters were strictly maintained at 35 GHz frequency and 10 mW/cm^2^ power density. The MMW beam was directed onto the sample using a 30-mm-diameter Gaussian beam-forming horn antenna. To ensure consistent and controlled MMW exposure, the output energy was continuously monitored in real-time using a calibrated power detection system (Virginia Diodes Inc. WR28PWM) throughout the irradiation process. During the experiment, the MMW source is closely attached to the test cell culture dish or animal tumor for testing (n=3).

Instead of direct tumor temperature measurement, the thermal response of the samples was assessed by monitoring the temperature change of water-filled containers during MMW irradiation. The infrared thermal imaging system (FLIR A655sc) is used to record the temperature change under the condition of strictly controlling the room temperature of 25℃, and provide the temporal and spatial temperature distribution.Thermal data were acquired at 1 frame per second and analyzed using proprietary software (FLIR ResearchIR Max 4.40.3).

**Culture of cell line and subsequent experimental treatment**

The murine breast cancer 4T1 cells, murine macrophage cell lines Raw 264.7 and J774.A1, human umbilical vein endothelial cells (HUVEC), as well as the human monocytic THP-1 cells were obtained from the American Type Culture Collection (ATCC, USA). The murine colon carcinoma CT26 cells were purchased from the Cell Bank of the Chinese Academy of Sciences (Shanghai, China).

4T1 cells were maintained in Dulbecco's Modified Eagle's Medium (DMEM, Gibco) supplemented with 10% fetal bovine serum (FBS, Gibco), 100 U/mL penicillin, and 100 μg/mL streptomycin (Invitrogen) at 37°C in a humidified atmosphere containing 5% CO_2_. Cells were passaged every 2-3 days using 0.25% trypsin-EDTA solution (Gibco) to detach cells when they reached 80-90% confluence.

CT26 cells were cultured in RPMI 1640 medium (Gibco) supplemented with 10% FBS, 100 U/mL penicillin, and 100 μg/mL streptomycin. The culture conditions were similar to 4T1 cells, with cells being passaged at 80-90% confluence using 0.25% trypsin-EDTA.

HUVEC cells were cultured in Endothelial Cell Growth Medium (EGM-2, Lonza) supplemented with 2% FBS, 0.1% vascular endothelial growth factor (VEGF, PeproTech), 0.1% recombinant human fibroblast growth factor (hFGF, PeproTech), 0.1% insulin-like growth factor (R3-IGF, Sigma-Aldrich), 0.1% ascorbic acid (Sigma-Aldrich), and 0.04% hydrocortisone (Sigma-Aldrich). Cells were maintained at 37°C with 5% CO_2_ and passaged at 90-95% confluence using 0.25% trypsin-EDTA solution, with passage number limited to P6.

Raw 264.7 and J774.A1 murine macrophage cell lines were cultured in DMEM (Gibco) supplemented with 10% FBS, 100 U/mL penicillin, and 100 μg/mL streptomycin. Cells were passaged every 2-3 days by scraping adherent cells when they reached 70-80% confluence.

THP-1 human monocytic cells were maintained in RPMI 1640 medium supplemented with 10% FBS, 100 U/mL penicillin, 100 μg/mL streptomycin, 0.05 mM 2-mercaptoethanol (Gibco), and 1 mM sodium pyruvate (Gibco). THP-1 cells were kept in suspension and passaged every 3-4 days by diluting the cell suspension to maintain a density between 2×10^5^ and 8×10^5^ cells/mL.

All cell lines were maintained in a humidified incubator at 37°C with 5% CO_2_.

MMW exposure was administered only once per sample, specifically on the day indicated in the experimental flowchart (see Main Text), with the irradiation duration varying depending on the experimental group assignment. 4T1, CT26, HUVEC, and macrophage/monocyte cell lines (Raw 264.7, J774.A1, and THP-1) were subjected to MMW irradiation (35 GHz, 10 mW/cm^2^) at room temperature (25°C) under humidified conditions with 5% CO_2_. Specifically, irradiation durations were: 0, 15, 30, and 45 minutes for 4T1, CT26, and macrophage/monocyte cells; 0, 5, 10, 15, 20, and 30 minutes for HUVEC. Cells were seeded in 35-mm dishes and allowed to attach overnight before MMW exposure.

**Assessment of Cell Viability**

Cell viability was assessed by MTT (3-(4,5-dimethylthiazol-2-yl)-2,5-diphenyltetrazolium bromide) (Sigma-Aldrich) method. Briefly, Raw264.7, J774.A1, THP-1 and HUVEC cells were inoculated into 96-well plates. For HUVEC, cells were exposed to MMW for 0, 5, 10, 15, 20, and 30 min (n=3); Raw264.7, J774.A1 and THP-1 were exposed for 0, 15, 30 and 45 min (n=3); 4T1 and CT26 cells were exposed for 0, 5, 10, 15 and 30 min (n=3). After separate treatments, MTT solution was added to each well and then incubated at 37°C for 4 hours. Thyroxine crystals were then dissolved with dimethyl sulfoxide (Sigma-Aldrich) and absorbance was measured at 570 nm using a microplate reader. Cell survival was calculated as a percentage of control for each cell line and treatment time.

To directly evaluate the immediate viability of cells following MMW irradiation, a Live/Dead Cell Viability/Cytotoxicity Assay Kit was employed for staining and observation. For observation under a laser scanning confocal microscope, cells were seeded directly into 24-well plates containing pre-placed coverslips. At the time of staining, the old culture medium was aspirated, and the cells were gently washed twice with pre-warmed PBS. Subsequently, the staining working solution was prepared according to the manufacturer's instructions: 2 μL of a 2 mM Calcein-AM stock solution and 8 μL of a 1.5 mM propidium iodide (PI) stock solution were added to 1 mL of serum-free medium (SFM) and mixed thoroughly. Then, 500 μL of the staining working solution was added to each well, ensuring complete coverage of the cells. The culture plate was incubated at 37°C in a cell culture incubator for 30 minutes in the dark. After incubation, the staining solution was removed, and the cells were gently rinsed twice with pre-warmed PBS to eliminate residual dye. The coverslips were carefully removed from the wells, inverted, and mounted onto glass slides with a drop of anti-fade mounting medium. Observation and image acquisition were performed immediately using a laser scanning confocal microscope. The green fluorescence from Calcein-AM (excitation/emission wavelengths: ~495 nm/~515 nm) was used to label live cells exhibiting esterase activity, while the red fluorescence from PI (excitation/emission wavelengths: ~535 nm/~617 nm) was used to label dead cells with compromised plasma membranes. Images were captured from multiple random fields of view (n=3).

4T1 or CT26 cells were seeded at a density of approximately 3×10^5^ cells per well in 6-well plates (or at 3×10^4^ cells per well in 24-well plates containing coverslips for confocal microscopy) and pre-cultured for 24 hours in DMEM supplemented with 10% FBS to allow for cell attachment and optimal growth. Subsequently, cells were subjected to MMW irradiation at different time points (0, 15, 30 minutes) with a power density of 10 mW/cm^2^, according to the experimental group assignments. Following irradiation, cells were continued in culture under the same conditions for 24 hours.For flow cytometric analysis, cells from each group were collected. Briefly, after two gentle washes with PBS, cells were detached using an appropriate amount of trypsin-EDTA solution, followed by neutralization with DMEM containing 10% FBS. The cell suspension was centrifuged at 1000 rpm for 5 minutes, and the pellet was resuspended in PBS to an appropriate concentration. Staining was performed using an Annexin V-FITC/PI Apoptosis Detection Kit. In brief, 100 μL of cell suspension was incubated with 5 μL of Annexin V-Fluorescein Isothiocyanate (Annexin V-FITC) and 5 μL of PI staining solution for 15 minutes in the dark. Subsequently, 400 μL of binding buffer was added to dilute the sample, which was then immediately analyzed by flow cytometry. By adjusting the instrument parameters, four cell populations were distinguished: viable cells (FITC⁻/PI⁻), early apoptotic cells (FITC⁺/PI⁻), late apoptotic cells (FITC⁺/PI⁺), and necrotic cells (FITC⁻/PI⁺). The percentage of early apoptotic cells was calculated for each group and compared to the control group to assess the impact of MMW irradiation on the apoptosis process (n=3).

**Animal model construction and subsequent experimental treatment**

Female BALB/c nude mice (6-8 weeks old) were purchased from Beijing Dynamic River Laboratory Animal Technology Co. All animal experiments were conducted in accordance with the Guidelines for the Care and Use of Laboratory Animals and were approved by the Institutional Animal Care and Use Committee (IACUC).

Unilateral 4T1 breast cancer model: BALB/C mice were divided into three groups in the single MMW treatment experiments of PBS (Gibco), 15 min and 30 min (n=6 mice/group). BALB/c nude mice were injected subcutaneously with 1 × 10^6^ 4T1 cells on the right side. On the 7th day of tumor inoculation, the mice in the corresponding groups were irradiated with local MMW for 15 or 30 minutes respectively. BALB/C mice were divided into four groups in the MMW+α-PD-L1 combination treatment experiments of PBS, α-PD-L1, MMW and MMW+α-PD-L1 (n=6 mice/group). BALB/c nude mice were injected subcutaneously with 1 × 10^6^ 4T1 cells on the right side. On the 7th day of tumor inoculation, mice in the corresponding groups were irradiated with local MMW for 30 minutes. On the 1st and 5th day after MMW irradiation, mice were intraperitoneally injected with anti-PD-L1 antibody (BioXCell, clone 10F.9G2, 200 μg per mouse) for a total of 2 times. Tumor volume was measured every 2 days with calipers using the formula (length×width^2^)/2. Tumor size was measured by two independent investigators who were unaware of the treatment group, and each measurement was averaged in triplicate (n=3). Upon reaching the experimental endpoint, mice were euthanized.

Bilateral 4T1 breast cancer model: BALB/C mice were divided into three group: PBS, MMW and MMW+α-PD-L1 (n=6 mice/group). 1×10^6^ 4T1 cells were injected subcutaneously into the right side of BALB/c nude mice. On the 4th day after the first inoculation, 1×10^6^ 4T1 cells were injected subcutaneously into the left side of BALB/c nude mice. On the 7th day of the first inoculation, mice in the responding groups were irradiated with local MMW for 30 minutes. On the 1st and 5th day after MMW irradiation, mice were intraperitoneally injected with anti-PD-L1 antibody for a total of 2 times. The tumor volume on each side was measured and calculated as described above.

4T1 tumor recurrence model: BALB/C mice were divided into three groups: PBS, MMW, and MMW+α-PD-L1 (n=6 mice/group). BALB/c nude mice were injected subcutaneously with 1×10^6^ 4T1 cells on the right side. On the seventh day of inoculation, mice in the corresponding group were irradiated with local millimeter waves for 30 minutes. On the first and fifth days after millimeter wave irradiation, anti-PD-L1 antibodies were injected into the abdominal cavity of mice for a total of 2 times. The tumor volume on each side was measured and calculated as described above. Tumor volume was measured every 2 days with calipers using the formula (length×width^2^)/2. Tumor size was measured by two independent investigators who were unaware of the treatment group, and each measurement was averaged in triplicate (n=3). On day 14 after the first implantation, the "primary" 4T1 tumors were surgically excised. The wound was closed with surgical clips or sutures. 5th day after surgery, mice were re-inoculated with 1×10^6^ 4T1 cells subcutaneously on the contralateral side. Tumor recurrence and growth were monitored as described above.

CT26 colon cancer model: BALB/C mice were divided into three groups: PBS, MMW, and MMW+α-PD-L1 (n=6 mice/group). BALB/c mice were subcutaneously inoculated with 5 × 10^5^ CT26 cells on the right side. On the 7th day of tumor inoculation, mice in the corresponding groups were irradiated with local MMW for 30 minutes. On the 1st and 5th day after MMW irradiation, mice were intraperitoneally injected with an anti-PD-L1 antibody for a total of 2 injections. Tumor volume was measured every 2 days and calculated as (length × width^2^)/2. Tumor size was measured by two independent investigators who were unaware of the treatment group, and each measurement was averaged in triplicate (n=3). Mice were euthanized when the experimental endpoint was reached.

Each group of mice was treated according to the experimental design, and body weight was recorded every 1 day as an indicator of overall health and treatment tolerance.

**Tumor Measurement**

Tumor size is measured every 2 days using calipers (n=3), and tumor volume is calculated using the formula: Tumor volume (mm^3^) = (length×width^2^)/2. Measurements begin 7 days after tumor inoculation and continue until the endpoint of the experiment, or when the tumor volume reaches 1500 mm^3^, at which time the mice will be euthanized according to the guidelines of the research institution. The tumor growth inhibition rate (IR) was calculated as: IR (%) = [1 - (mean tumor weight of treatment group / mean tumor weight of control group)]×100%. In the bilateral 4T1 model, tumor volumes were measured and reported separately for both sides. In the tumor recurrence model, the volumes of the primary and recurrent tumors were recorded separately. Body weight was recorded every 1 day as an indicator of overall health status and treatment tolerance. Mice with >20% weight loss or significantly impaired body condition are removed from the study and euthanized humanely.

**Histological Analysis**

At the endpoint of the experiment, tumor tissue was removed from the mouse body and weighed for histological analysis. After fixing the tumor tissue, make paraffin sections and perform routine hematoxylin-eosin (H&E) staining (Sigma-Aldrich) to observe the morphological changes of the tumor tissue. At the same time, immunohistochemical staining was performed on the slices to detect the expression level of proliferation-related marker Ki67 and to evaluate the proliferation status of tumor cells. In addition, Terminal Deoxynucleotidyl Transferase mediated dUTP Nick-End Labeling (TUNEL) fluorescence staining (Roche) was used to detect the apoptosis level of tumor cells.

The specific steps are as follows:

Making paraffin sections: fixing fresh tumor tissue in a 4% paraformaldehyde solution (Sigma-Aldrich) for 24 hours, routine dehydration, transparency, and paraffin embedding, using a semi-automatic slicing mechanism to prepare 4 μM thick paraffin sections.

H&E staining: routine dewaxing and re-staining, staining with hematoxylin solution (Sigma-Aldrich) for 15 minutes, staining with 1% eosin solution (Sigma-Aldrich) for 3 minutes, step-by-step dehydration, transparency, and sealing.

Immunohistochemical staining: Follow the instructions of the reagent kit to perform pre-treatment steps such as antigen repair, endogenous peroxidase blockade, and non-immune blockade. Add primary antibody (anti-Ki67 monoclonal antibody, dilution ratio 1:200, Abcam) and incubate overnight at 4℃. The next day, add biotinylated secondary antibody (Jackson ImmunoResearch) and incubate at room temperature for 30 minutes. DAB color solution (Vector Laboratories) is used for color development, followed by re-dyeing with hematoxylin, followed by stepwise dehydration, transparency, and film sealing. Positive cells are brownish-yellow granules.

TUNEL staining: Follow the instructions of the reagent kit for operation. After pre-treatment of the slices, add the TUNEL mixture and incubate at 37℃ in the dark for 1 hour. After restaining with DAPI (Sigma-Aldrich), the cell nucleus was sealed and observed. The apoptotic cell nucleus shows red fluorescence, while the living cell nucleus shows blue fluorescence.

The above staining results were observed under an optical microscope and photographed for retention. Count Ki67 and TUNEL positive cells and calculate the positivity rate. For each tumor sample (n = 6 mice per group), 5 randomly selected fields per slide were analyzed (n = 5).

**Flow Cytometry Analysis**

1. **Tumor-Conditioned Media Stimulated Dendritic Cell Analysis by Flow Cytometry**

Cell source and preparation: Bone marrow-derived dendritic cells (BMDCs) were generated by culturing mouse bone marrow cells in the presence of GM-CSF (PeproTech) and interleukin-4 (IL-4, PeproTech) for 7 days. The BMDCs were then treated with tumor-conditioned media (collected from 4T1 tumor cell culture) for 24 hours to mimic the effects of the tumor microenvironment.

Flow cytometry analysis: The BMDC samples were stained with fluorescent-conjugated antibodies targeting CD45, CD11b, MHC II, CD80, and CD86, and analyzed by flow cytometry. The proportions of mature dendritic cells (CD45^+^CD11b^+^MHC II^+^CD80^+^CD86^+^) were quantified and compared between different treatment groups. Tumor tissues of mice in each group were analyzed through three independent experiments (n=3).

1. **Analysis of Tumor-Draining Lymph Node Immune Cells by Flow Cytometry**

Cell source and preparation: the tumor, tumor drainage lymph nodes, and spleen in mice were taken out and prepared into a single-cell suspension.

Surface labeling and endogenous labeling: stained cells surface labeling with fluorescent monoclonal antibodies, such as CD45, CD11b, MHC II, CD80, CD86, CD3, CD4, CD8, CD49b, F4/80, CD206, etc. And endogenous markers, such as transcription factor FoxP3 and cytokine IFN-γ.

Flow cytometer analysis: BD FACSCanto II flow cytometer was used to analyze the labeled cells, and the Gating strategies were shown in Fig. 39S. The main cell populations analyzed include:

Dendritic cells (DCS): CD45^+^CD11b^+^CD11c^+^

Neutrophils: CD45^+^CD11b^+^Ly6g^+^

Macrophages: CD45^+^CD11b^+^F4/80^+^

CD4^+^ T cells: CD45^+^CD3^+^CD4^+^

CD8^+^ T cells: CD45^+^CD3^+^CD8^+^

Regulatory T cells (Tregs): CD4^+^CD25^+^FOXP3^+^

Activated CD8^+^ T cells: CD8^+^IFN-γ^+^ and CD8^+^CD69^+^

Central memory T cells (TCM): CD8^+^CD44^+^CD62L^+^

Effector memory T cells (TEM): CD8^+^CD44^+^CD62L^-^

M1 macrophage: CD45^+^CD11b^+^F4/80^+^CD86^+^

M2 macrophage: CD45^+^CD11b^+^F4/80^+^CD206^+^

Natural killer cells (NK cells): CD45^+^CD3^-^CD49b^+^

Analysis index: Compare the proportion of different immune cell subsets in different treatment groups (such as control group, MMW irradiation group, MMW combined with α-PD-L1 group, etc.), and evaluate the role of MMW irradiation combined with immune checkpoint inhibitor in activating anti-tumor immune response. At the same time, the contents of cytokines in tumor microenvironment were detected.

The specific antibodies (with clone numbers) used above include:

CD45 (Clone 30-F11)

CD11b (Clone M1/70)

CD11c (Clone N418)

MHC II (I-A/I-E) (Clone M5/114.15.2)

CD80 (Clone 16-10A1)

CD86 (Clone GL-1)

CD3 (Clone 17A2)

CD4 (Clone GK1.5 or RM4-5)

CD8a (Clone 53-6.7)

CD49b (Pan-NK cells) (Clone DX5)

F4/80 (Clone BM8)

CD206 (MMR) (Clone C068C2)

Ly6G (Clone 1A8)

CD25 (Clone PC61)

FoxP3 (Clone FJK-16s)(Intracellular)

IFN-γ (Clone XMG1.2)(Intracellular)

CD69 (Clone H1.2F3)

CD44 (Clone IM7)

CD62L (Clone MEL-14)

All antibody manufacturers were BioLegend.

Data collection and statistical analysis: collect the fluorescence signal data obtained by flow cytometry, and analyze the proportion of different cell groups by using professional software FlowJo (BD Biosciences). The tumor, tumor drainage lymph nodes, and spleen tissues of mice in each group were analyzed through three independent experiments (n=3).

**Enzyme-Linked Immunosorbent Assay (ELISA)**

The levels of cytokines (IFN-γ, TNF-α, IL-12, IL-6, IL-10, IL-12p70) in cell culture supernatant or tumor tissue lysate were determined by commercial ELISA kit (eBioscience or R&D Systems), and the operation was strictly carried out according to the kit instructions. All ELISA experiments were performed in triplicate and repeated in three independent experiments (n=3).

The specific steps are as follows: firstly, the captured antibody is dissolved with coating buffer (BioLegend) and coated on the bottom of a 96-well plate, and the coating is realized by overnight incubation at 4℃. The next day, the nonspecific binding site was blocked with a blocking solution (usually PBS buffer containing protein) and incubated at room temperature for 1-2 hours. Add the sample to be tested and the standard series, and incubate at room temperature to combine the antigen with the capture antibody. Unbound substances were washed off with PBST (PBS buffer containing Tween-20, Sigma-Aldrich).

A biotinylated detection antibody combined with another antigenic determinant of the target protein was added and incubated at room temperature. Then the enzyme marker streptavidin-HRP (Jackson ImmunoResearch) was added and incubated at room temperature to bind it to biotin. After washing to remove unbound substances, a suitable substrate is added to produce a detectable chromogenic reaction. The terminator stops the reaction, and the absorbance value (OD value) is determined at a specific wavelength of the microplate reader.

Finally, the data are corrected according to the standard curve, and the cytokine concentration is in direct proportion to the OD value so that the content of various cytokines in the sample can be quantitatively analyzed. The whole experiment was carried out in strict accordance with the standard operating specifications to ensure the reliability of the experimental data.

**Western Blotting**

Tumor tissues were lysed in Radio Immunoprecipitation Assay Lysis buffer (RIPA, Thermo Fisher Scientific)containing protease and phosphatase inhibitors, and protein concentrations were determined using the Bicinchoninic Acid Assay (BCA) Protein Assay Kit (Thermo Fisher Scientific). Equal amounts of protein (30 μg) were separated by SDS-PAGE (12% gel) and transferred onto PolyVinylideneFluoride (PVDF, Millipore) membranes. After blocking with 5% non-fat milk (Bio-Rad) or bovine serum albumin (BSA, Jackson ImmunoResearch) in TBS-T (Bio-Rad) for 2 hours at room temperature, membranes were incubated with primary antibodies against target proteins (PD-L1, CD47, CD38, cleaved Caspase-3, Bcl-2, Survivin, h2a.x-s139, MMP-9, MMP-8, MMP-13.etc, Abcam) overnight at 4°C, followed by incubation with HRP-conjugated secondary antibodies (Jackson ImmunoResearch) for 2 hours at room temperature. Protein bands were visualized using an enhanced chemiluminescence (ECL, Thermo Fisher Scientific) detection system and images were acquired using a ChemiDoc imaging system (Bio-Rad). Densitometric analysis was performed using ImageJ software (NIH, National Institutes of Health).

For loading controls, membranes were stripped and reprobed with antibodies against housekeeping proteins such as β-actin or GAPDH.

Protein expression levels were quantified by densitometry, normalized to the loading controls, and expressed as fold change relative to the control group. Data are presented as mean ± SEM from at least three independent experiments. Western blot was performed on tumor lysates from mice in each group, and the experiment was repeated three times independently (n=3).

**Molecular Dynamics Simulations**

The initial structures of the target proteins, including CD47 (PDB ID: 4CMM), CD38 (PDB ID: 1YH3), and transforming growth factor-β (TGF-β, PDB ID: 1TGK) were retrieved from the RCSB (PDB). Molecular dynamics (MD) simulations were conducted using the GROMACS software package (version 2022.4) to investigate the conformational changes, hydrogen bonding, and secondary structure alterations induced by MMW irradiation. Each simulation was performed in five runs (n=5).For each protein system, two sets of simulations were performed: one with an applied electric field mimicking MMW irradiation and the other without an electric field as a control. The protein molecules were solvated in a cubic box with periodic boundary conditions, using the TIP3P water model. An appropriate number of sodium (NA^+^) and chloride (CL^-^) counterions were added to neutralize the system's charge.

Energy minimization was carried out using the steepest descent algorithm for 5000 steps with a maximum force of 100.0 kJ/mol/nm to remove steric clashes and unfavorable geometries. Subsequently, the systems underwent equilibration under constant temperature (298.15 K) and pressure (1 bar) conditions for 100 ps. The temperature was controlled using the velocity-rescale thermostat with a coupling constant of 0.2 ps, while the pressure was maintained using the Berendsen barostat with a coupling constant of 0.5 ps and an isotropic compressibility of 4.5 × 10^-5^ bar^-1^.

we used *E(t) = A·****u*** *cos (ωt + ϕ)* to describe the terahertz electric field, where ***u*** is the unit vector to describe the direction of the electric field, *A* is the electric field strength set to 1.6 V/nm, *ω* is the angular frequency *(ω = 2πν)*, and *ϕ* is the initial phase. The magnetic field component was omitted because GROMACS does not directly support the incorporation of magnetic fields in simulations. Additionally, for MMW irradiation, the electric field strength is typically orders of magnitude higher than the magnetic field strength, making the electric field contribution the dominant factor affecting the system's dynamics. Therefore, only the electric field component was considered in these simulations as a reasonable approximation^83, 84^. The production runs were performed for 20 ns with a time step of 2 fs, employing the leapfrog integrator algorithm. Periodic boundary conditions were applied, and long-range electrostatic interactions were calculated using the Particle Mesh Ewald (PME) method with a real-space cutoff of 1.0 nm. The van der Waals interactions were truncated at 1.0 nm, and the long-range dispersion corrections were applied for energy and pressure.

The trajectories were analyzed using various GROMACS analysis tools, such as VMD (version 1.9.3) to investigate the conformational changes, hydrogen bonding patterns, and secondary structure alterations induced by MMW irradiation. ‘rms’ and ‘rmsf’ commands used to study the root-mean-square deviation (RMSD) and root-mean-square fluctuation (RMSF) of protein as a whole and specified residues. Use VMD to perform Beta analysis on the B-factor file generated by RMSF to generate the B-factor diagram. The Ramachandran diagram of protein is described by the ‘rama’ command. The distance between the centers of mass is completed by the ‘distance’ command. Hydrogen bonding analysis was performed using a geometric criterion, where a donor-acceptor distance of less than 0.35 nm and a donor-hydrogen-acceptor angle greater than 120 degrees were considered as hydrogen bonds. The secondary structure assignments were made using the DSSP (Dictionary of Secondary Structure of Proteins) algorithm implemented in GROMACS.

The results of the MD simulations, including the conformational changes, hydrogen bonding patterns, and secondary structure alterations, were analyzed and interpreted in the context of the potential effects of MMW irradiation on the structural dynamics and stability of the target proteins.

**Metabolomics and Proteomics Analysis**

For metabolomics analysis, tumor tissues were snap-frozen in liquid nitrogen and stored at -80°C until analysis. Samples were extracted using a methanol (Fisher Scientific) -water mixture (4:1, v/v), and metabolite profiling was performed using liquid chromatography-mass spectrometry (LC-MS). An Agilent 1290 LC system coupled to an Agilent 6540 Q-TOF mass spectrometer was employed for data acquisition in both positive and negative ionization modes. Data were processed using dedicated software (XCMS, Scripps Research Institute) for peak detection, alignment, and statistical analysis. Differentially expressed metabolites between experimental groups were identified by volcano plot analysis and hierarchical clustering analysis. A total of 6 upregulated and 1 downregulated metabolites were detected in the 30-minute MMW irradiation group compared to the control PBS group. Metabolomic analysis was performed on tumor tissues of mice in each group, and each sample was analyzed in duplicate (n=3).

For proteomic analysis, tumor tissue samples were collected from both the 30-minute MMW-irradiated group and the untreated control group, with the following specific steps: Fresh tumor tissues were minced into 1-2 mm^3^ pieces, washed three times with pre-cooled PBS to remove impurities, and then digested into a single-cell suspension using a solution containing 2 mg/mL collagenase IV (37°C, 30 minutes) (Sigma-Aldrich). The suspension was filtered through a 70 μm cell sieve to remove undigested debris, followed by centrifugation (4°C, 300×g, 10 minutes) to collect the cell pellet. Trypan blue staining (Gibco) indicated a cell viability >95%, with the absolute cell count recorded as 5.2×10^6^ cells/g for the control group and 4.8×10^6^ cells/g for the MMW group, showing no significant difference between groups. The cell pellet was lysed in lysis buffer (100 mM Tris-HCl, 1% NP-40, 1% SDS, Sigma-Aldrich) on ice for 30 minutes, sonicated, and centrifuged to collect the supernatant. Protein concentration was determined using the BCA method. Equal amounts of protein (50 μg) were subjected to reduction, alkylation, and digestion with trypsin (enzyme:substrate ratio 1:50, 37°C, 16 hours, Promega), followed by LC-MS/MS analysis using an Eksigent 425 liquid chromatography system (C18 column, 5%-35% acetonitrile (Fisher Scientific) gradient over 120 minutes) and a Sciex 5600+ TripleTOF mass spectrometer. Protein identification and quantification were performed using MaxQuant (v2.0.3, Max Planck Institute of Biochemistry) matched against the UniProt database. Differentially expressed proteins were filtered using |log_2_ fold change| ≥ 0.1 and p < 0.05. The tumor tissues of mice in each group were analyzed by proteomics, and each sample was analyzed in duplicate (n=3).

**Immunofluorescence Staining and Confocal Microscopy**

For immunofluorescence staining, 4T1 murine breast cancer cells, RAW 264.7 murine macrophages, J774.A1 murine macrophages, and THP-1 human monocytic cells were cultured under appropriate conditions. Cells were seeded onto sterile glass coverslips in 6-well plates or 35 mm glass-bottom dishes and allowed to adhere overnight. For tumor tissue sections, excised tumor samples were fixed in 4% paraformaldehyde and embedded in paraffin before sectioning.

For staining, cells or tumor tissue sections were fixed with 4% paraformaldehyde for 15 minutes at room temperature, permeabilized with 0.1% Triton X-100 (Sigma-Aldrich) for 10 minutes, and blocked with 1% BSA in PBS for 30 minutes to reduce non-specific antibody binding.Samples were then incubated with primary antibodies against target proteins diluted in blocking buffer overnight at 4°C. The following primary antibodies were used: anti-calreticulin (Abcam) (1:200 dilution), anti-high mobility group box 1 (HMGB-1,1:500 dilution, Abcam), anti-CD206 (1:100 dilution, M2 macrophage marker, BioLegend), and anti-CD86 (1:200 dilution, M1 macrophage marker, BioLegend). After washing with PBS, samples were incubated with appropriate fluorescent-labeled secondary antibodies (1:500 dilutions) for 2 hours at room temperature in the dark.

For nuclear counterstaining, samples were incubated with 4',6-diamidino-2-phenylindole (DAPI, 1 μg/mL) for 10 minutes. Coverslips were mounted onto glass slides using an anti-fade mounting medium (Invitrogen ProLong Gold).

Fluorescence images were acquired using a confocal laser scanning microscope (Zeiss LSM880) equipped with 405 nm, 488 nm, 561 nm, 640 nm laser lines. Image acquisition settings were kept consistent for all samples within the same experiment. Images were processed and analyzed using ZEN software (Zeiss).

For quantitative analysis, 100 cells from three independent experiments (n=3) were randomly selected from each sample, and the mean fluorescence intensity (MFI) of the target proteins was measured. The MFI data were normalized to the control condition and presented as mean ± standard deviation (SD).

**Macrophage Differentiation**

For macrophage differentiation studies, the murine macrophage cell line Raw 264.7 was used (n=3). Cells were cultured in DMEM supplemented with 10% FBS, 100 U/mL penicillin, and 100 μg/mL streptomycin at 37°C in a humidified 5% CO_2_ incubator.

To induce polarization into M1 or M2 phenotypes, Raw 264.7 cells were seeded in 6-well plates at a density of 3×10^5^ cells/well. After overnight attachment, cells were treated with IL-4 (20 ng/mL) to prime them for polarization. Simultaneously, cells were exposed to MMW at 35 GHz for different time durations (0, 15, 30, and 45 minutes) using an MMW irradiation system with a power density of 10 mW/cm^2^.

Following MMW exposure, cells were further cultured for 48 hours in the presence of polarizing stimuli. For M1 polarization, cells were treated with 100 ng/mL lipopolysaccharide (LPS, Sigma-Aldrich) and 20 ng/mL interferon-gamma (IFN-γ, R&D Systems). For M2 polarization, cells were treated with 20 ng/mL IL-4 and 20 ng/mL interleukin-13 (IL-13, PeproTech). Untreated cells served as the control group.

After the 48-hour polarization period, cells were harvested for analysis of macrophage phenotypes by evaluating the expression of surface markers CD206 (M2 marker) and CD86 (M1 marker) using flow cytometry or confocal microscopy.

**ATP Release Assay**

The levels of extracellular ATP released from 4T1 cells were quantified using an ATP fluorometric assay kit (Promega, G8231) according to the manufacturer's instructions. Briefly, 4T1 cells were seeded in 96-well plates at a density of 1×10^4^ cells/well and allowed to adhere overnight. The next day, cells were exposed to MMW irradiation for varying durations of 0 (control), 15, and 30 minutes. After the respective irradiation times, the culture supernatants were collected and centrifuged at 3000 rpm for 5 minutes to remove any detached cells and debris.

The supernatant samples were then diluted 50-fold with the assay buffer provided in the kit. In a standard opaque 96-well plate, 50 μL of each diluted sample was mixed with 50 μL of the ATP reaction mixture, which contained luciferase and luciferin. The plate was incubated at room temperature for 10 minutes in the dark to allow the luminescent reaction to occur. The luminescence intensity, which is proportional to the ATP concentration, was measured using a microplate reader (BioTek Cytation 5) with an integration time of 0.5 seconds per well.

A standard curve was generated using known concentrations of ATP provided in the kit, and the sample ATP concentrations were calculated by interpolating the luminescence values against the standard curve. The data were expressed as mean ± standard deviation (SD) of triplicate samples (n=3).

**Assessment of Liver and Kidney Function**

Serum levels of alanine aminotransferase (ALT), aspartate aminotransferase (AST), alkaline phosphatase (ALP), blood urea nitrogen (BUN), and creatinine (CRE) in mice were measured using commercial assay kits (Nanjing Jiancheng Bioengineering Institute) to evaluate the effects of MMW irradiation on liver and kidney function (n=3).

Briefly, blood samples were collected from the mice via cardiac puncture under anesthesia at the end of the experiment. The blood samples were allowed to clot for 30 minutes at room temperature and then centrifuged at 3,000 rpm for 15 minutes to separate the serum. The serum was carefully transferred to fresh microcentrifuge tubes and stored at -80°C until analysis.

For the measurement of ALT, AST, and ALP levels, the respective assay kits (C009-2/C010-2/A059-2) were used according to the manufacturer's instructions. In brief, 200 μL of serum was mixed with the respective reagents provided in the kits, and the enzymatic reactions were monitored spectrophotometrically at 340 nm (ALT, AST) and 405 nm (ALP) using a microplate reader (SpectraMax M5). The enzyme activities were calculated from the absorbance values using standard curves prepared with known concentrations of the respective enzymes.

For the measurement of BUN and CRE levels, the respective assay kits (C013-2/C011-2) were used according to the manufacturer's instructions. In brief, 200 μL of serum was mixed with the respective reagents provided in the kits, and the colorimetric reactions were monitored spectrophotometrically at 540 nm (BUN) and 510 nm (CRE) using a microplate reader (SpectraMax M5). The concentrations of BUN and CRE were calculated from the absorbance values using standard curves prepared with known concentrations of urea and creatinine, respectively.

The results were expressed as mean ± standard error of the mean (SEM) for each group (n = 6 mice per group). Statistical analysis was performed using one-way analysis of variance (ANOVA) followed by Tukey's multiple comparison test. A p<0.05 was considered statistically significant.

**Statistical Analysis**

Data are presented as mean±standard deviation (SD) and were derived from at least three independent replicates (the specific n value is described below). All datasets were assessed for normality using the Shapiro-Wilk test prior to parametric testing. Statistical differences between two groups were analyzed using a two-tailed unpaired Student’s t-test (assuming normally distributed data and homogeneity of variance), while comparisons involving multiple groups were performed using one-way analysis of variance (ANOVA) followed by Tukey’s post hoc test for multiple comparison correction. Analyses were conducted using GraphPad Prism 9.0 (GraphPad Software, Inc., CA, USA).Statistically significance thresholds were defined as follows: **p* < 0.05 (significant), ***p* < 0.01 (moderately significant), and ****p* < 0.001 (highly significant)

Sample Size and Replication Summary:

All experiments were performed with appropriate replicates to ensure statistical reliability:

Cell culture experiments: All cell viability assays were performed with three independent experiments (n=3). Animal experiments: Six mice per treatment group were used for all in vivo tumor models, including unilateral 4T1, bilateral 4T1, 4T1 tumor recurrence, and CT26 colon cancer models. Tumor measurements were performed by two independent investigators with triplicate measurements averaged for each data point (n=3). Histological analysis: Six mice per group (n=6) were analyzed, with 5 randomly selected fields examined per slide (n=5 fields). Flow cytometry: Both tumor-conditioned media stimulated dendritic cell analysis and tumor-draining lymph node immune cell analysis were performed in three independent experiments (n=3). Molecular assays: ELISA experiments were performed in triplicate with three independent experiments (n=3). Western blotting was performed on tumor lysates from each treatment group with three independent experiments (n=3). Molecular dynamics simulations: Each protein system was simulated in five independent runs (n=5). Omics analyses: Metabolomics and proteomics analyses were performed on tumor tissues with three replicates per group (n=3), with each sample analyzed in duplicate. Immunofluorescence: Quantitative analysis was performed on 100 randomly selected cells from three independent experiments (n=3). Functional assays: Macrophage differentiation studies and ATP release assays were performed in triplicate with three independent experiments (n=3). Serum biochemistry: six mice per group (n = 6) were subjected to three (n = 3) assessments of liver and kidney function. MMW exposure system calibration and thermal monitoring were performed in triplicate (n=3).

**Gromacs Files Information**

1. Energy minimization file

define = -DFLEXIBLE

integrator = cg

nsteps = 5000

emtol = 100.0

emstep = 0.01

;

nstxout = 50

nstlog = 50

nstenergy = 50

;

pbc = xyz

cutoff-scheme = Verlet

coulombtype = PME

rcoulomb = 1.0

vdwtype = Cut-off

rvdw = 1.0

DispCorr = EnerPres

;

constraints = none

1. Restrictive dynamics file

define = -DPOSRES

integrator = md

dt = 0.001 ; ps

nsteps = 50000 ; 100ps

comm-grps = system

refcoord-scaling = com

energygrps =

;

nstxout = 0

nstvout = 0

nstfout = 0

nstlog = 500

nstenergy = 500

nstxout-compressed = 1000

compressed-x-grps = system

;

pbc = xyz

cutoff-scheme = Verlet

coulombtype = PME

rcoulomb = 1.0

vdwtype = cut-off

rvdw = 1.0

DispCorr = EnerPres

;

Tcoupl = V-rescale

tau_t = 0.2

tc_grps = system

ref_t = 298.15

;

Pcoupl = Berendsen

pcoupltype = isotropic

tau_p = 0.5

ref_p = 1.0

compressibility = 4.5e-5

;

constraints = hbonds

1. Long-term formal dynamic document

integrator = md

dt = 0.002 ;

nsteps = 25000000 ;

comm-grps = protein

comm-mode = angular

energygrps =

;

nstxout = 0

nstvout = 0

nstfout = 0

nstlog = 5000

nstenergy = 1000

nstxout-compressed = 1000

compressed-x-grps = system

;

pbc = xyz

cutoff-scheme = Verlet

coulombtype = PME

rcoulomb = 1.0

vdwtype = cut-off

rvdw = 1.0

DispCorr = EnerPres

;

Tcoupl = V-rescale

tau_t = 0.2 0.2

tc_grps = protein non-protein

ref_t = 298.15 298.15

;

Pcoupl = parrinello-rahman

pcoupltype = isotropic

tau_p = 2.0

ref_p = 1.0

compressibility = 4.5e-5

;

constraints = hbonds

;

electric-field-x =1.6 0.22 0 0

**Take the simulation of TGF-β protein as an example.**

gmx pdb2gmx -f 1TGK.pdb -o 1TGK.gro -p topol.top

gmx editconf -f 1TGK.gro -o 1TGK_box.gro -d 1.2 -bt cubic

gmx solvate -cp 1TGK_box.gro -o 1TGK_SOL.gro -p topol.top

gmx grompp -f em.mdp -c 1TGK_SOL.gro -p topol.top -o em.tpr -maxwarn 1

（gmx editconf -f complex.gro -o complex_box.gro -d 1.2 -bt cubic

gmx solvate -cp complex_box.gro -o complex_SOL.gro -p topol.top

gmx grompp -f em.mdp -c complex_SOL.gro -p topol.top -o em.tpr -maxwarn 1

）

gmx genion -s em.tpr -o system.gro -p topol.top -pname NA -nname CL -neutral

gmx grompp -f em.mdp -c system.gro -p topol.top -o em.tpr

gmx mdrun -v -deffnm em

gmx grompp -f pr.mdp -c em.gro -p topol.top -r em.gro -o pr.tpr

gmx mdrun -v -deffnm pr

gmx grompp -f md.mdp -c pr.gro -p topol.top -o md.tpr -maxwarn 2

(gmx grompp -f md.mdp -c pr.gro -p topol.top -o md.tpr -n index.ndx -maxwarn 2

)

gmx mdrun -v -deffnm md

**Trajectory analysis, removing the phenomenon of random link of trajectories.**

gmx trjconv -s md.tpr -f md.xtc -o md1.xtc -dt 500 -pbc mol

gmx trjconv -s md.tpr -f md.gro -o md1.gro -pbc mol

**Trajectory dewatering**

gmx trjconv -f md1.xtc -s md.tpr -o md_nowat.xtc

gmx trjconv -f md1.gro -s md.tpr -o md_nowat.gro

**Optimizing random links in VMD**

mol bondsrecalc all

mol reanalyze all

**Key CA atomic index in VMD**

set sel [atomselect top "resid 48 76 and name CA"]

$sel get index

**Check mirror distance**

gmx mindist -f md1.xtc -s md.tpr -pi

**RMSD**

gmx rms -s md. tpr -f md_nowat.xtc -o rmsd_protein.xvg

**Designated residue RMSD**

gmx make_ndx -f md.tpr -o index.ndx

r 35 37 100 101 102 103 104

q

gmx rms -s md.tpr -f md_nowat.xtc -o rmsd_key.xvg -n index.ndx

**RMSF**

gmx rmsf -s md.tpr -f md_nowat.xtc -o rmsf_protein.xvg -oq bfac.pdb -res -b 250

gmx rmsf -s md.tpr -f md_nowat.xtc -o rmsf_protein.xvg -oq bfac.pdb -res -b 250 -n index.ndx

**B-factor graph**

Bfac.pbd

**Ramachandran graph**

gmx rama -f md.xtc -s md.tpr

dit xvg_rama -f rama.xvg

qtgrace rama.xvg

**Minimum distance between atoms of each residue pair**

gmx mdmat -f md.xtc -s md.tpr

gmx xpm2ps -f dm.xpm -bx 15 -by 15 -rainbow blue

**Hydrogen bond**

gmx make_ndx -f md.tpr -o index.ndx

gmx hbond -f md1.xtc -s md.tpr -dist -ang -life -nhbdist -n index.ndx

**Measure the distance change between the center and geometric center of residual matrix**

gmx distance -s md.tpr -f md.xtc -select “com of resid 95 plus com of resid 107” -oall

gmx pairdist -s md.tpr -f md.xtc -ref “resid 101" -sel "resid 103”

gmx distance -s md.tpr -f md1.xtc -n index.ndx -select “cog of group “”1”” plus cog of group “”18””” -oall

index 517 or index 548 or index 1523 or index 1538 or index 1557 or index 1571 or index 1595
