# Supplementary figures and images for: Reviving Dormant Immunity: Millimeter Waves Reprogram the Immunosuppressive Microenvironment to Potentiate Immunotherapy without Obvious Side Effects
Source: Cyborg Bionic Syst. 2025 Dec 10;6:0468. doi: 10.34133/cbsystems.0468 (PMC12695134; doi:10.34133/cbsystems.0468)

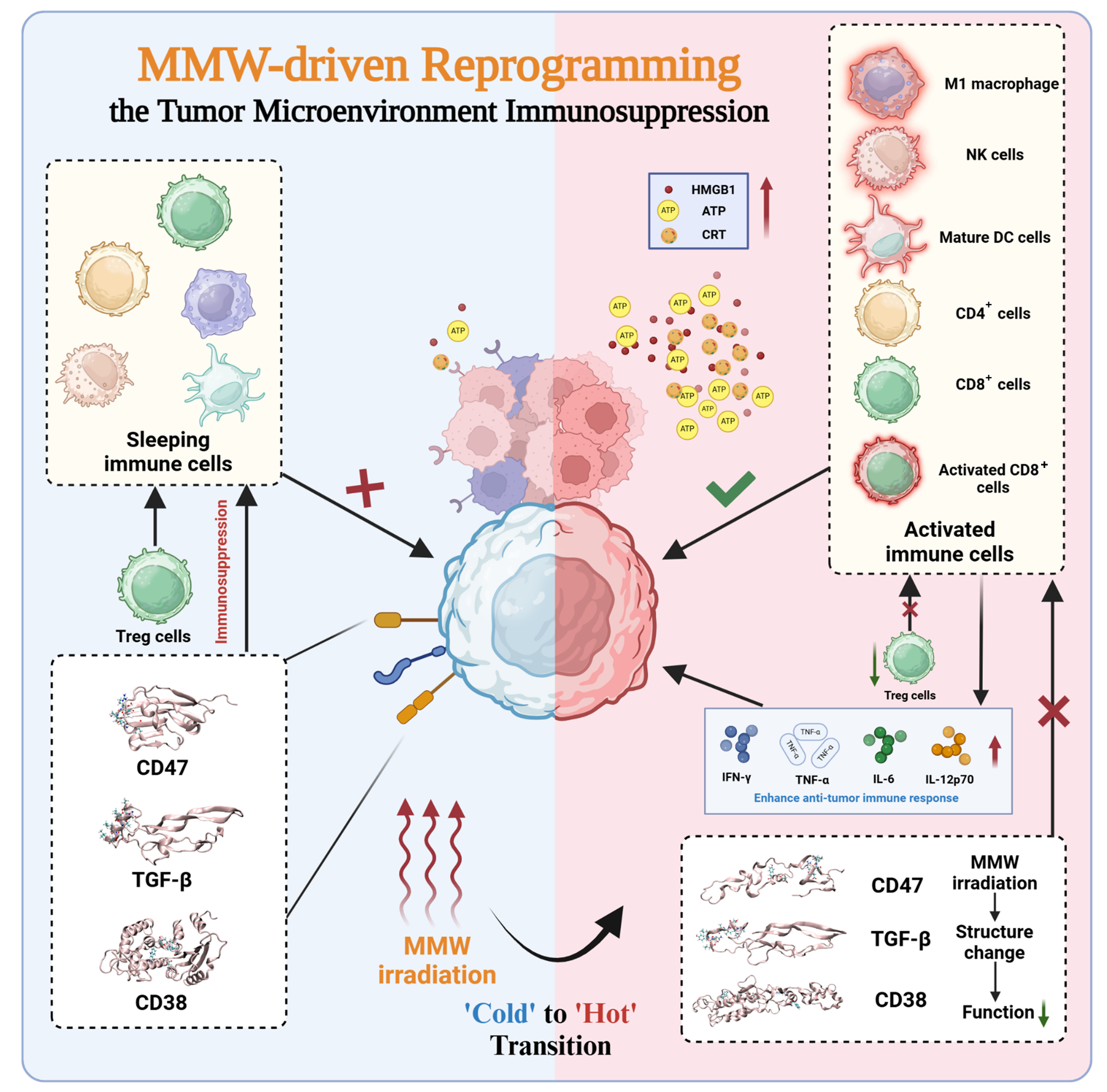

Supplement: Supplementary 1 — Graphical Abstract Supplementary Materials and Methods Gromacs Files Information Figs. S1 to S33 [file cbsystems.0468.f1.zip › Graphical Abstract.TIFF]
